# Supplementary material for: Identification of novel differentially methylated sites with potential as clinical predictors of impaired respiratory function and COPD
Source: eBioMedicine. 2019 Mar 29;43:576–86. doi: 10.1016/j.ebiom.2019.03.072 (PMC6557748; doi:10.1016/j.ebiom.2019.03.072)
Supplement: Supplementary file 1 — Supplementary material [file mmc1.docx]

**Supplementary Material**

**Identification of novel differentially methylated sites with potential as clinical predictors of impaired respiratory function and COPD.**

Table of Contents

[Supplementary Methods 1](#_Toc523491562)

[Population samples 1](#_Toc523491563)

[Ethics 1](#_Toc523491564)

[DNA extraction 1](#_Toc523491565)

[Genome‐wide methylation profiling 1](#_Toc523491566)

[Trait data 2](#_Toc523491567)

[Identification of differentially methylated positions 3](#_Toc523491568)

[Sensitivity analyses 3](#_Toc523491569)

[Probe annotation and epigenetic regulation of gene expression 3](#_Toc523491570)

[Prediction 4](#_Toc523491571)

[Case-control data 4](#_Toc523491572)

[Model selection 4](#_Toc523491573)

[Model evaluation 5](#_Toc523491574)

[Supplementary Tables 6](#_Toc523491575)

[Supplementary Figures 26](#_Toc523491576)

# Supplementary Methods

## Population samples

The Generation Scotland Scottish Family Health Study (GS:SFHS) consists of participants (≥18 years of age at recruitment) who have been densely profiled for clinical, lifestyle, and sociodemographic variables and for whom genome-wide genotype data is available.^1,2^

The Lothian Birth Cohort of 1936 (LBC1936) consists of participants (∼70 years of age at recruitment) with extensive phenotype data on psycho-social, lifestyle, genetic and health measures.^3,4^

## Ethics

All components of GS:SFHS received ethical approval from the NHS Tayside Committee on Medical Research Ethics (REC Reference Number: 05/S1401/89). GS:SFHS has also been granted Research Tissue Bank status by the Tayside Committee on Medical Research Ethics (REC Reference Number: 10/S1402/20), providing generic ethical approval for a wide range of uses within medical research. Ethical permission for the LBC1936 was obtained from the Multi-Centre Research Ethics Committee for Scotland (MREC/01/0/56) and the Lothian Research Ethics Committee (LREC/2003/2/29). Written informed consent was obtained from all participants in the GSSFHS and LBC1936.

## DNA extraction

Whole blood genomic DNA extraction in the GS:SFHS was performed using a Nucleon Kit (Tepnel Life Science) with the BACC3 protocol. DNA concentrations (ng/μl) were determined for all samples using the Picogreen method (Invitrogen). Eight out of every batch of 92 samples were electrophoresed on a 1% agarose gel to test for integrity of the DNA. Eight samples from each were also run on a NanoDrop (Thermo Scientific) to confirm DNA yield and to determine levels of protein and RNA contamination.^5^ A detailed protocol describing DNA measurement in the LBC1936 has been presented previously.^6^ Briefly, DNA was extracted from 1,004 whole blood samples in LBC1936. Samples were extracted at the Wellcome Trust Clinical Research Facility (WTCRF), Western General Hospital, Edinburgh, using standard methods.

## Genome‐wide methylation profiling

Blood DNA methylation data was acquired for 5,190 GS:SFHS participants.^7^ Whole blood genomic DNA (500 ng) samples were treated with sodium bisulphite using the EZ-96 DNA Methylation Kit (Zymo Research, Irvine, California), following the manufacturer’s instructions. DNA methylation was assessed using the Infinium MethylationEPIC BeadChip, in accordance with the manufacturer’s protocol. The arrays were scanned using a HiScan scanner (Illumina Inc., San Diego, California) and initial inspection of array quality was carried out using Genome Studio v2011.1.

Additional quality control (QC) measures were implemented in R.^8^ Raw intensity (.idat) files were read into R using the *minfi* package.^9^ The R package *shinyMethyl* was then used to plot the log median intensity of methylated versus unmethylated signal per array. ^10^ Outliers from this QC plot were visually identified and excluded from further analysis. Methylation beta-values were then entered into the ‘*pfilter’* function in the R package *wateRmelon* which was used to exclude poor performing samples and probes.^11^ Samples were excluded if ≥ 1% sites had a detection p-value of > 0.05. Probes were removed from the dataset if: (i) they had more than five samples with a beadcount of less than 3; or (ii) ≥ 0.5% samples had a detection p-value of > 0.05; or (iii) had failed in any sample. Finally, *shinyMethyl*’s sex prediction plot was used to exclude samples whose predicted sex did not match recorded sex. The data were then normalized using the ‘*dasen’* function from the R package *wateRmelon*.^11^ Background differences between Type I and Type II assays were adjusted by adding the offset between Type I and II intensities to Type I intensities. Between‐array quantile normalization was then performed for the methylated and unmethylated signal intensities separately. Prior to downstream analyses, probes on the X and Y chromosomes were removed^12,13^ and M‐values, defined as M=log2([M+100]/[U+100]), where M represents the methylated signal intensity and U represents the unmethylated signal intensity, were calculated for the normalized data ^14^. Finally, probes were removed from the dataset if they contained a single nucleotide polymorphism (SNP) in the final five bases from the 3’ end with a minor allele frequency of at least 1% in the European population from the 1000 Genomes Project^15^ or they were predicted to cross‐hybridize.^16,17^ Following QC, the dataset comprised M-values for 735,418 methylation loci measured in 5,190 individuals.

To assess intra-individual differences in cellular composition, estimated cell counts for B‐lymphocytes, granulocytes, monocytes, natural killer cells, CD4+ T‐lymphocytes and CD8+ T‐lymphocytes were generated using the ‘*estimateCellCounts’* function in *minfi*. This function implements Jaffe and Irizarry's^18^ modified version of Houseman's algorithm.^19^ As the GS:SFHS is a family-based study, a correction for relatedness was implemented. We calculated the genetic relationship matrix (GRM), which contains a measure of the genetic correlation among individuals using genome-wide genotyping data^20^ in the software package Genome‐wide Complex Trait Analysis (GCTA).^21^ The M-values were then residualised by adjusting for array processing batch, imputed cellular composition and the random effect of genetic relatedness using a linear mixed effects model in DISSECT.^22^

DNA methylation data were assessed in 1,004 whole blood samples from the LBC1936 using the Illumina HumanMethylation450 BeadChip (Illumina Inc., San Diego, CA). Full details of sample preparation and methylation typing have been reported previously.^4,6,23^ In brief, background correction was performed, and quality control was used to remove probes with a low detection rate (P>0.01 for >5% of samples), low quality (manual inspection), and low call rate (P < 0.01 for <95% of probes) and probes on the X and Y chromosomes. Samples where predicted sex, based on XY probes, did not match reported sex were removed. After these QC steps, 450,726 autosomal probes remained with 920 samples available for analysis. Before downstream analyses, M‐values were calculated and corrected for Houseman's imputed cellular composition, effects of array processing batch, sample plate, and hybridization date using a linear model. Residuals from this model were used in subsequent analyses.

## Trait data

Respiratory function was assessed by FEV_1_ and FVC. FEV_1_ and FVC were measured in litres using spirometry.^1^ Spirometry was performed three times without nose clips using the Ndd Easy One Spirometer (Model 2001) and Micro Medical Spirometer in the GS:SFHS and LBC1936 respectively. Only pre-bronchodilator spirometry measures were available. The maximum values of FVC and FEV_1_ were used in the analyses. Spirometric measurements fulfilled the European Respiratory Society (ERS)/American Thoracic Society (ATS) requirements.^24^

Participation was voluntary, so some participants may have opted not to undertake spirometry assessment. Furthermore, GS:SFHS participants were excluded from spirometry testing if they were <12 or > 24 weeks pregnant, had had a stroke or heart attack in the previous year, used an inhaler and did not have it with them, had had a collapsed lung, flu, severe cold, chest infection or any surgery in the previous month or had had a detached retina in previous 3 months.^25^ As missing values do not relate specifically to respiratory health, and as such should be missing at random and should not introduce bias in analyses.

Quality control of the phenotype data was undertaken to exclude participants with inaccurate spirometry or covariate data. In total, 4,193 GS:SFHS participants with DNA methylation data had spirometry data. Participants with missing height (n=8) and weight (n=5) records were removed. Participants with extreme outlier values (those with values more than three times the interquartile distances away from either the 75th or the 25th percentile values) for FEV_1_ (n=1), height (n=1) and weight (n=6) were excluded to eliminate participants not representative of normal variation within the population. A single participant who reported never smoking, but had smoked >0 pack years was removed leaving 4,171 records. Finally, the full GS:SFHS dataset was then divided into an incident COPD dataset (described below) for prediction analysis and a discovery EWAS dataset. To ensure the independence of the prediction data, participants and individuals closely related to participants (identity-by-state [IBS] > 0.05)^26^ in the prediction data were removed; leaving 3,781 GS:SFHS records for inclusion in the discovery sample for EWASs. In the LBC1936 replication data, 895 participants with DNA methylation data had spirometry data, none of whom had extreme outlier values.

The percent predicted FEV_1_ was calculated according to the previously described National Health and Nutrition Examination Survey (NHANES) III spirometric reference equations.^27^ Individuals with percent predicted FEV_1_ less than 80% and FEV_1_/FVC less than 0.7 (Global Initiative for Chronic Obstructive Lung Disease [GOLD] stages 2–4) were classified as COPD case subjects.^28^ Individuals with FEV_1_ greater than 80% predicted and FEV_1_/FVC greater than 0.7 were classified as control subjects. Individuals not falling in either category (GOLD stage 1; FEV_1_/FVC < 0.7, FEV_1_ ≥ 80% predicted) were excluded to minimize potential misclassification of COPD case subjects and control subjects.^24^

Linear regression was used to pre-correct FEV_1_, FVC and FEV_1_/FVC and COPD for age, age^2^, sex, height, height^2^, and two smoking variables: smoking status (current, former [quit < 12 months ago], former [quit ≥ 12 months ago] and never smoker) and pack-years of smoking (calculated by multiplying the number of packs [20 cigarettes] smoked per day by the number of years the participant has smoked). FVC was pre-corrected for the effect of weight in addition to the above variables. The residuals were transformed to ranks and then transformed to normally distributed z-scores,^29^ using the ‘*rntransform’* and ‘*ztransform’* functions in the *GenABEL* R package respectively.^30^ These transformed residuals were then used as the phenotype for epigenome-wide association studies (EWAS).

## Identification of differentially methylated positions

To reduce the impact of any technical biases which occurred during methylation profiling,^31^ we performed principal component analysis (PCA) on the genome-wide probe intensities using the ‘*prcomp’* function in the *stats* R package. The first 20 principal components (PCs), explaining 98% of the variance in genome-wide probe intensities, were stored. A linear regression model was then run with residualised (for array processing batch, estimated cell counts and genetic relatedness) M-values for each CpG site as the dependent variable and respiratory function traits (FEV_1_, FVC and FEV_1_/FVC) or COPD, as well as age, sex, smoking status (a categorical variable called “ever smoked” with the levels “current smoker”, “former smoker who quit < 12 months ago”, “former smoker who quit ≥ 12 months ago” and “never smoked”), pack-years and the 20 methylation PCs as the independent variables. Effect estimates are given as β coefficients with standard error (SE) drawn from the linear regression model.

The *limma* package in R was used to run the linear regression models over the 735,418 methylation loci included in the analysis.^32^ All statistical analyses were performed using M-values as they are more statistically robust.^14^ Analyses were performed using R version 3.4.4 (The R Foundation for Statistical Computing) and the Bioconductor package version 3.6. Correction for multiple testing was implemented using the genome-wide significance threshold of ~3.6×10^−8^.^33^

DNA methylation at nearby DMSs can be highly correlated.^34^ We used the *coMET* package to identify regional co-methylation patterns and regional results.^35^

## Sensitivity analyses

Smoking has a profound impact on respiratory function,^36^ COPD risk^5,39^ and DNA methylation.^37–40^ To date, studies have not corrected both the DNA methylation data and trait data for smoking history.^13,23,41–43^ To assess the impact of pre-correction of the traits for smoking history, we undertook sensitivity analyses, in which FEV_1_, FVC, FEV_1_/FVC, and COPD were not pre-corrected for smoking status and pack-years. Lung function remains steady before 40 years of age and starts declining after that,^44,45^ and COPD is predominantly diagnosed in adults aged over 40 years.^28^ Smoking is associated with impaired lung function,^45,46^ and is the major risk factor for COPD.^28,47^ Furthermore, we do not know if pre-bronchodilator restrictive spirometry patterns were reversible in this study. To assess the generalisability of the estimated effects in older adults, according to smoking status (ever smokers and non-smokers), smoking history, and restrictive spirometry. The data was truncated by age (> 40 years),^28^ smoking history (≥ 10 pack-years; smokers with a substantial smoking history)^48^ and non-restrictive spirometry (individuals with a FEV_1_/FVC ≥ 0.7 and FVC or FEV_1_ < 80% of predicted were excluded),^49,50^ and stratified smoking status (ever smokers and non-smokers). For these analyses, the trait data were transformed separately, and we conducted separate random-effects meta-analyses using *metafor* package in R for each trait-specific genome-wide significant differentially methylated site (DMS).^51^ To test the existence of heterogeneity we used the Cochrane's χ2 statistics with a significance level of 0.05. Inconsistency indexes (I^2^) were also calculated, and a value greater than 50% was considered an indicator of high inconsistency.^52^

## Probe annotation and epigenetic regulation of gene expression

All DNA methylation probes were assigned to enhancer regions, CpG regions (island, shore, shelf, or open sea), and gene-centric locations (TSS1500: 200-1500 nucleotides upstream of the transcriptional start site (TSS); TSS200: TSS to 200 nucleotides upstream of the TSS; 5′ untranslated region (UTR); 1st exon; gene body; 3′UTR; and intergenic region (IGR)) based on the IlluminaHumanMethylationEPICanno.ilm10b2.hg19 R package. For each trait, methylation probes were filtered at p<0.01, and probes that mapped to genes extracted. To perform functional enrichment tests of the mapped genes, we used Database for Annotation, Visualization and Integrated Discovery (DAVID; http://david.abcc.ncifcrf.gov/tools.jsp) bioinformatics resources database^53^ for biological processes and molecular function analysis and used the Ingenuity Pathway Analysis (IPA; h[ttp://www.ingenuity.com](http://www.ingenuity.com/)) software for canonical pathway enrichment analyses. Although DAVID can perform pathway enrichment tests, the IPA system provides a more comprehensive pathway resource based on manual collection and curation. Briefly, DAVID implements the hypergeometric test for the enrichment of Gene Ontology (GO) terms and UniProt keywords in the candidate genes. The IPA system implements Fisher's exact test to determine whether a canonical pathway is enriched with genes of interest. The Benjamini-Hochberg false discovery rate method was used to correct for multiple testing.^54^ Adjusted p-values of <0.05 were considered significant.

To generate hypotheses about the effects of the methylation changes observed in this study, we integrated our findings with publically available data from a genome-wide analysis of gene expression (GWAGE) in lung tissue samples from current and ex-smokers with (n=311) and without COPD (n=270).^55^ Using the hg19 genome build and default settings within the *Significance-based Modules Integrating the Transcriptome and Epigenome (*SMITE) R package we constructed a framework where each gene was associated with a promoter region (+/− 1000 bp from the transcription start site) and a gene body region (transcription start site  + 5000 bp to transcription end site).^56^ The EWAS regression coefficients and p-values for each of the respiratory function traits or COPD, and the GWAGE in COPD z-scores and p-values were then separately combined into the framework using Stouffer’s method, and p-values normalised using a rescaled logit transform. We set the relationship between expression and methylation to “bidirectional” in both gene regions to avoid biasing the results, and genes were scored based on a weighted significance value (0.4 for expression, 0.4 for promoter methylation, and 0.2 for body methylation).^57^ Gene scores were considered significant at p < 0.05. Trait-specific modules were then identified by inputting gene scores into a Reactome protein-protein interaction graph spin-glass algorithm.^58^ Significant gene modules (p < 0.05 and 10–500 genes) were subjected to KEGG pathway enrichment analysis within the SMITE R package.^56^ A gene module is a gene co-expression network to which the same set of transcription factors binds.^59^ We set the false discovery rate threshold for each term at 5%.

## Prediction

### Case-control data

GS:SFHS participant data were linked at an individual level to the national hospitalisation register (Scottish Morbidity Record, SMR01) records.^20^ We used the International Classification of Disease, Tenth Version (ICD-10) J40 to J44 codes to identify those hospital admissions due to COPD exacerbations.^48,60–71^ Incident cases were defined as any hospital admission in which the primary diagnosis was assigned an ICD code for COPD since recruitment to GS:SFHS. During follow-up, 81 of the 5,091 participants with DNA methylation data developed incident COPD. Incident cases were all 40 years or older. It is well known that the performance of classification models may be negatively impacted by imbalanced training data^72–75^, as the model, may overfit the majority class and underfit the minority class, yielding biased results^76,77^. Therefore, to obtain a well-balanced dataset for training of the models control subjects age ≥ 40 years were, therefore, selected at random from the participants with DNA methylation data in a 1:1 ratio to case participants. Control participants met the following conditions: (1) no self-report nor SMR01 record of COPD and (2) baseline FEV_1_ greater than 80% and predicted and FEV_1_/FVC greater than 0.7. Participants with missing height (N=1) and weight records (N=2) were excluded. In order to minimise bias caused by related individuals, closely related individuals (IBS > 0.05) were removed; leaving 149 records (71 COPD cases, 78 controls) in the prediction data set. The data were then separated into a training set of 95 participants (47 COPD cases, 48 controls) and a test set of 55 participants (24 COPD cases, 30 controls).

Hospitalisation register records were not available for LBC1936 participants. Case-control status was therefore defined based on spirometry data. The percent predicted FEV_1_ was calculated according to the previously described National Health and Nutrition Examination Survey (NHANES) III spirometric reference equations.^27^ Participants with predicted FEV_1_ less than 80% and FEV_1_/FVC less than 0.7 were classified as COPD case subjects.^28^ Individuals with FEV_1_ greater than 80% predicted and FEV_1_/FVC greater than 0.7 were classified as control subjects. In total, 89 of the 817 participants with DNA methylation data had prevalent COPD. Control subjects were selected at random from the participants with DNA methylation data in a 1:1 ratio to case participants.

We performed principal component analysis (PCA) on the genome-wide probe intensities in the training, test and replication data separately. Pre-correction of the genome-wide significant CpG sites for age, sex, ever smoked, pack-years and the 20 PCs was then conducted separately for the test, training and replication datasets.

### Model selection

For the training data, the reduced model, which included the clinical risk factors age, age^2^, sex, height, height^2^, current, former (quit ≥ 12 months ago) and never smoker (analysed as 0/1 dummy variables), and pack-years of smoking^78–80^ was constructed using unpenalised logistic regression. Former smokers who quit < 12 months ago were combined with current smokers due to insufficient number of records. The full model, which included DMSs and clinical risk factors was constructed using penalised logistic regression with an elastic net penalty. To improve model interpretability and to determine any significant improvement in prediction accuracy, the regression coefficients of the clinical variables in the reduced model were not penalised, meaning that they were retained in all models. Whereas, the regression coefficients of the DMSs were allowed to have different penalties. Elastic net regression is controlled by two tuning parameters, (i) alpha, which sets the degree of mixing between two types of regularised regression, namely ridge regression (alpha = 0) and Least Absolute Shrinkage and Selection Operator, (LASSO, alpha = 1), and (ii) lambda, defining the strength of regularisation. Tuning parameters for model selection were based on varying levels for alpha and lambda. The selection of the full models was conducted based on 10-fold cross-validation against the training data (Figure 1). The optimal model was selected based on the tuning parameters with a maximum mean area under the curve (AUC) across all folds. Final models were constructed using the complete training set and evaluated on the independent GS:SFHS test and LBC1936 replication datasets. The model selection procedure was conducted using the R package *caret*.

### Model evaluation

Comparison of the prognostic/diagnostic performance of the baseline clinical model and baseline clinical model incorporating differentially methylated sites was carried out using receiver operating characteristic (ROC) analysis. The area under the ROC curve (AUC) indicates the ability of a model to discriminate between individuals with COPD versus those who do not. A score of 0.5 indicates that a model has no discriminatory ability, while a score of 1 indicates that presences and absences are perfectly discriminated. We performed non-parametric ROC tests using a stratified bootstrap sampling method to estimate differences between AUC of ROC curves. Assessment of the incremental value of the DMSs to predict the COPD risk, when added to the model with established clinical predictors was carried out using the integrated discrimination improvement (IDI), and net reclassification improvement (NRI) measures. We used binary NRI, with the optimal, cutpoint (assuming equal importance for the sensitivity and specificity)^81^ for the reduced and full model to avoid overestimated reclassification from continuous NRI.^82^ Finally, we performed decision curve analysis to estimate the potential clinical usefulness of the models. All statistical analyses were two-sided, and p-values less than 0.05 were considered significant. The AUC of ROC curves were calculated and compared using the R packages *pROC*. All analyses were conducted in R version 3.5.0.

# Supplementary Tables

**Table S1.** Summary of epigenome-wide association study results for the three respiratory function traits and COPD in the discovery (all), older adult (> 40 years) and stratified smoking status datasets from the Generation Scotland: Scottish Family health study cohort.

| **Trait** | **FEV_1_** | **FVC** | **FEV_1_/FVC** | **COPD** |
| --- | --- | --- | --- | --- |
| **N_all_** | 3781 | | | 3193 |
| **λ_all_** | 1.09 | 1.08 | 1.04 | 1.12 |
| **DMS_all_** | 17 | 7 | 3 | 2 |
| **DMS_all-smoking_** | 9 | 2 | 3 | 0 |
| **N_>40_** | 2853 | | | 2352 |
| **λ_>40_** | 1.05 | 1.05 | 1.04 | 1.10 |
| **DMS_>40_** | 6 | 1 | 2 | 2 |
| **DMS_>40-smoking_** | 3 | 1 | 2 | 0 |
| **N_non-smokers_** | 1948 | | | 1699 |
| **λ_non-smokers_** | 1.06 | 1.06 | 1.01 | 1.11 |
| **DMS_non-smokers_** | 1 | 2 | 0 | 3 |
| **DMS_non-smokers-smoking_** | 0 | 0 | 0 | 0 |
| **N_non-smokers >40_** | 1436 | | | 1236 |
| **λ_non-smokers >40_** | 1.04 | 1.04 | 1.02 | 1.09 |
| **DMS_non-smokers >40_** | 0 | 0 | 0 | 3 |
| **DMS_non-smokers >40-smoking_** | 0 | 0 | 0 | 0 |
| **N_smokers_** | 1729 | | | 1412 |
| **λ_smokers_** | 1.03 | 1.01 | 1.02 | 1.09 |
| **DMS_smokers_** | 1 | 0 | 2 | 2 |
| **DMS_smokers-smoking_** | 1 | 0 | 2 | 0 |
| **N_smokers >40_** | 1340 | | | 1058 |
| **λ_smokers >40_** | 1.02 | 1.00 | 1.01 | 1.07 |
| **DMS_smokers >40_** | 2 | 0 | 1 | 0 |
| **DMS_smokers >40-smoking_** | 2 | 0 | 1 | 0 |
| **N_smokers >10py_** | 936 | | | 726 |
| **λ_smokers >10py_** | 1.00 | 0.98 | 0.99 | 1.03 |
| **DMSs_smokers >10py_** | 0 | 0 | 0 | 0 |
| **DMSs_smokers >10py-smoking_** | 0 | 0 | 0 | 0 |
| **N_smokers >10py >40_** | 829 | | | 634 |
| **λ_smokers >10py >40_** | 1.01 | 0.99 | 0.99 | 1.02 |
| **DMSs_smokers >10py >40_** | 0 | 0 | 0 | 0 |
| **DMSs_smokers >10py >40-smoking_** | 0 | 0 | 0 | 0 |
| **N_NRS_** | 3418 | | | 3092 |
| **λ_NRS_** | 1.02 | 1.06 | 1.02 | 1.09 |
| **DMSs_NRS_** | 1 | 3 | 1 | 0 |
| **DMSs_NRS-smoking_** | 1 | 0 | 1 | 0 |

**Key:** **N**, sample size; **all**, all data; **λ**, genomic inflation factor; **DMS**, genome-wide significant differentially-methylated CpG sites; **smoking**, number of DMSs that were also genome-wide significant in an EWAS for ever-smoked in the Generation Scotland: Scottish Family Health Survey (GS:SFHS) cohort; **>40**, data from participants aged 40 or greater, **non-smokers**, data from non-smokers; **smokers**, data from smokers, **>10py**, data from participants with a smoking history of 10 pack years or greater, **NRS,** data from participants with non-restrictive spirometry pattern.

### Table S2. Summary results for the genome-wide significant differentially methylated sites (DMSs) showing genome-wide significant association with at least one respiratory function trait or chronic obstructive pulmonary disease (COPD) across all traits in the Generation Scotland Scottish Family Health Study (GS:SFHS) discovery data. Results are ordered by chromosomal location.

| **CpG site** | **FEV_1_** | | **FVC** | | **FEV_1_/FVC** | | **COPD** | |
| --- | --- | --- | --- | --- | --- | --- | --- | --- |
|  | **β** | **p-value** | **β** | **p-value** | **β** | **p-value** | **β** | **p-value** |
| **cg13993467** | **-0.023** | **5.19E-10** | **-0.025** | **1.17E-11** | 0.001 | 8.66E-01 | 0.015 | 4.32E-03 |
| **cg16963852** | **0.015** | **2.88E-08** | 0.013 | 1.26E-06 | 0.003 | 2.77E-01 | -0.010 | 7.82E-03 |
| **cg26804423** | **-0.011** | **4.88E-11** | -0.008 | 1.74E-06 | -0.001 | 5.20E-01 | 0.003 | 1.61E-01 |
| **cg26080684** | **-0.010** | **9.75E-09** | -0.007 | 1.17E-04 | -0.001 | 4.28E-01 | 0.007 | 7.56E-03 |
| **cg01198738** | **0.013** | **5.12E-09** | **0.011** | **6.91E-07** | -0.002 | 4.10E-01 | -0.004 | 2.26E-01 |
| **cg03770138** | **0.010** | **2.34E-08** | 0.009 | 2.58E-06 | -0.001 | 6.60E-01 | -0.001 | 6.08E-01 |
| **cg18871648** | **0.014** | **4.30E-09** | **0.012** | **3.01E-07** | 0.000 | 9.08E-01 | -0.005 | 1.93E-01 |
| **cg10919522** | **0.014** | **2.14E-10** | 0.012 | 4.76E-08 | 0.001 | 5.84E-01 | -0.003 | 3.99E-01 |
| **cg09018739** | **-0.008** | **5.49E-10** | -0.006 | 6.76E-06 | -0.002 | 5.74E-02 | 0.005 | 6.91E-03 |
| **cg07687574** | **0.012** | **6.68E-11** | **0.010** | **6.17E-07** | 0.003 | 1.50E-01 | -0.007 | 1.27E-02 |
| **cg19748455** | **0.015** | **1.11E-09** | 0.011 | 3.47E-06 | 0.000 | 9.42E-01 | -0.008 | 1.94E-02 |
| **cg18181703** | **0.011** | **4.51E-09** | 0.008 | 9.07E-06 | 0.001 | 7.67E-01 | -0.012 | 1.99E-05 |
| **cg11047325** | **0.018** | **3.91E-11** | 0.014 | 1.28E-06 | 0.002 | 5.55E-01 | -0.016 | 1.21E-04 |
| **cg13343932** | **0.014** | **6.52E-10** | 0.010 | 1.24E-05 | 0.002 | 3.80E-01 | -0.013 | 5.58E-05 |
| **cg18608055** | **0.010** | **5.52E-09** | 0.006 | 6.42E-04 | 0.002 | 2.49E-01 | -0.005 | 2.99E-02 |
| **cg02370334** | **0.011** | **3.48E-08** | 0.008 | 2.83E-05 | 0.001 | 5.33E-01 | -0.007 | 1.80E-02 |
| **cg03187361** | **0.011** | **1.39E-08** | **0.011** | **5.43E-08** | 0.001 | 6.00E-01 | -0.005 | 1.11E-01 |
| **cg01620970** | **-0.011** | **1.16E-07** | **-0.012** | **4.11E-09** | -0.002 | 4.68E-01 | 0.011 | 4.30E-04 |
| **cg00213822** | -0.014 | 1.35E-05 | **-0.020** | **5.23E-11** | 0.007 | 2.04E-02 | 0.005 | 3.15E-01 |
| **cg15659943** | -0.008 | 1.31E-06 | **-0.009** | **1.64E-08** | 0.005 | 4.13E-03 | -0.001 | 7.71E-01 |
| **cg25465557** | -0.008 | 2.51E-04 | **-0.013** | **2.11E-10** | 0.006 | 2.46E-03 | 0.007 | 3.27E-02 |
| **cg18007249** | **-0.011** | **6.98E-08** | **-0.013** | **5.60E-10** | 0.001 | 7.59E-01 | 0.002 | 4.97E-01 |
| **cg13108341** | **-0.028** | **9.12E-07** | **-0.032** | **2.57E-08** | -0.001 | 8.47E-01 | 0.018 | 3.44E-02 |
| **cg00045592** | 0.001 | 7.95E-01 | -0.008 | 6.02E-04 | **0.014** | **1.22E-08** | -0.007 | 5.86E-02 |
| **cg05575921** | 0.022 | 1.11E-05 | 0.004 | 3.63E-01 | **0.029** | **3.48E-09** | -0.031 | 1.41E-05 |
| **cg03636183** | **0.014** | **2.36E-07** | 0.005 | 5.60E-02 | **0.016** | **5.10E-09** | -0.011 | 4.79E-03 |
| **cg09455379** | 0.008 | 3.54E-02 | 0.003 | 4.65E-01 | 0.009 | 9.75E-03 | **-0.030** | **3.28E-08** |
| **cg20453862** | 0.007 | 1.48E-01 | -0.001 | 8.53E-01 | 0.014 | 3.37E-03 | **-0.039** | **7.42E-09** |

### Key: β, regression coefficient; FEV_1_, Forced expiratory volume in one second; FVC, Forced vital capacity. Genome-wide significant (p <3.6×10^−8^) ^33^and suggestive associations (p<1.0×10^−6^)are highlighted in bold red and blue respectively.

**Table S3.** Gene functions and related diseases of identified differentially methylated sites (DMSs) associated with respiratory function traits or chronic obstructive pulmonary disease (COPD) in the Generation Scotland Scottish Family Health Study (GS:SFHS) discovery data. The gene information was obtained from GeneCards and systematic term (gene name with “DNA methylation”, “lung”, “COPD”, “asthma”, or “lung cancer”) search in PubMed.

| **Gene (DMSs)** | **Gene function and related diseases** |
| --- | --- |
| ***ABCA1***  **(**cg15659943**)** | *ABCA1* (ATP Binding Cassette Subfamily A Member 1) mediates the release of cholesterol from activated cholesterol–filled macrophages, initiating the formation of high-density lipoproteins (HDL) particles.^83^ Diseases associated with *ABCA1* include COPD,^84^ non-small cell lung cancer,**^85^** Tangier disease,^86^ hypoalphalipoproteinemia,^87^ atherosclerosis^83^, and Alzheimer’s disease.^88^ *ABCA1* plays an important role in respiratory function and disease,^89,90^ and *ABCA1* overexpression in mice lung tissue has been associated with airway inflammation.^91^ *ABCA1* knock out mice have abnormal lung morphology and physiology.^89^ It is also significantly down-regulated in the lung upon smoke exposure conditions.^92^ Genome-wide studies have consistently identified polymorphisms in *ABCA1* associated with HDL cholesterol.^93,94^ Differential methylation in *ABCA1* in lung tissue has been reported to be associated with pulmonary arterial hypertension.^95^ Epigenome-wide association studies (EWAS) have also reported that differential methylation in *ABCA1* is associated with type 2 diabetes^96^ and coronary artery disease.^97–99^. |
| ***AHNAK***  **(**cg25465557**)** | AHNAK (*AHNAK* nucleoprotein) is a scaffolding protein that regulates cytoskeletal structure formation, muscle regeneration, calcium homeostasis, and signalling. ^100–104^ Diseases associated with *AHNAK* include COPD^105^ and lung adenocarcinoma.^106,107^ A SNPs in *AHNAK* have been shown to associate with COPD^105^ and asthma.^108^ Differential methylation in *AHNAK* has been reported to be associated with lung cancer.^107,109^ |
| ***AHRR***  **(**cg05575921**)** | *AHRR* (Aryl-Hydrocarbon Receptor Repressor) is a tumour repressor and key regulator for metabolizing carcinogens from tobacco smoke.^110–112^. *AHRR* methylation in blood has been associated with smoking,^113–115^ respiratory function,^116^ COPD,^115^ lung cancer,^112,117^ occupational exposure to polycyclic aromatic hydrocarbons,^118^ proliferative diabetic retinopathy^119^ and cardiovascular disease.^120^ |
| ***C14orf43***  **(**cg18871648,  cg10919522**)** | *C14orf43* (a.k.a. *ELMSAN1*; ELM2 And Myb/SANT Domain Containing 1) is involved in chromatin binding.^121^ *C14orf43* has been associated with breast cancer.^122^ Differential methylation in *C14orf43* has been reported to be associated with smoking^123^ and type 2 diabetes.^124^ |
| ***CELSR1***  **(**cg03187361**)** | *CELSR1* (Cadherin EGF LAG Seven-Pass G-Type Receptor 1) is involved in the planar cell polarity pathway of lung branching morphogenesis, and mice with *CELSR1* mutations demonstrate abnormal lung development.^125^ Diseases associated with *CELSR1* include neural tube defects^126^ and [spina bifida](http://journals.plos.org/plosone/article?id=10.1371/journal.pone.0092207).^127^ SNPs in *CELSR1* have been shown to associate with COPD,^128^ and change in body mass index in COPD.^129^ Differential methylation in *CELSR1* has been reported to be associated with hepatocellular carcinoma.^130^ |
| ***CNTN4***  **(**cg13993467**)** | *CNTN4* (Contactin 4) is an axon-associated cell adhesion molecule that functions in neuronal network formation and plasticity.^131^ *CNTN4* may play a role in the formation of axon connections in the developing nervous system. Diseases associated with *CNTN4* include spinocerebellar ataxia type 16^132^ and chromosome 3pter-p25 deletion syndrome.^133^ Differential methylation in *CNTN4* has been reported to be associated with alcohol use,^134^ colorectal cancer^135^ and astrocytomas.^136^ |
| ***CPNE2***  **(**cg09018739**)** | *CPNE2* (Copine 2) is one of several genes that encode a calcium-dependent protein containing two N-terminal type II C2 domains and an integrin A domain-like sequence in the C-terminus.^121^ Little is known about the role of *CPNE2* in disease. Differential methylation in *CPNE2* has however been reported to be associated with osteoarthritis^137^ and obesity.^138^ |
| ***DNAH9***  **(**cg13108341**)** | DNAH9 (Dynein Axonemal Heavy Chain 9) is involved in respiratory cilia mobility and associated with abnormalities of pulmonary function.^139^ *DNAH9* is differentially expressed in the respiratory epithelium of patients with primary ciliary dyskinesia.^139^ A SNP in DNAH9 has been shown to interact with early-life environmental tobacco smoke.^140^ Differential methylation in *DNAH9* has also been reported in non-small-cell lung cancer.^141^ |
| ***F2RL3***  **(**cg03636183**)** | *F2RL3* (F2R Like Thrombin or Trypsin Receptor 3) encodes a member of the protease-activated receptor subfamily, part of the G-protein coupled receptor 1 family of proteins that plays roles in blood coagulation and inflammatory reactions.^121^ Differential methylation in *F2RL3* has been reported to be associated with smoking,^142,143^ COPD,^13^ lung cancer,^144,145^ occupational exposure to polycyclic aromatic hydrocarbons,^118^ IgG glycosylation^146^ and cardiovascular disease.^147,148^ |
| ***FKBP6***  **(**cg26080684**)** | *FKBP6* (FK506 Binding Protein 6) encodes a cis-trans peptidyl-prolyl isomerase that may function in immunoregulation and basic cellular processes involving protein folding and trafficking.^121^ Diseases associated with *FKBP6* include [Williams-Beuren syndrome](http://www.malacards.org/card/williams_beuren_syndrome)^149^ and [azoospermia](http://www.malacards.org/card/azoospermia).^150,151^ An epigenome-wide association study (EWAS) has reported that differential methylation in *FKBP6* is associated with cervical cancer.^152^ |
| ***GCNT2***  (cg20453862) | *GCNT2* (Glucosaminyl [N-Acetyl] Transferase 2 [I Blood Group]) encodes the enzyme responsible for formation of the blood group I antigen.^121^ Mutations in this gene have been associated with cataracts and the i blood group phenotype.^153^ Differential methylation in *GCNT2* has been reported in fetal and adult red blood cells,^154^ aldosterone-producing adenomas,^155^ and associated with lymph node metastasis in colorectal cancer.^156^ Functional studies have shown that ectopic expression of *GCNT2* enhances, while knockdown decreases lung metastasis in a mouse breast cancer model.^157^ |
| ***ICA1***  **(**cg26804423**)** | *ICA1* (Islet Cell Autoantigen 1) encode an islet cell auto antigen that is the target of auto antibodies in type I diabetes and Sjögren’s syndrome.^158^ Differential methylation in *ICA1* has been reported to be associated with type 2 diabetes^124^ and renal cell carcinoma.^159^ |
| ***JADE1***  **(**cg01620970**)** | *JADE1* (aka *PHF17*; Jade Family PHD Finger 1) is a negative regulator of Wnt signalling which has been linked to the pathogenesis and progression of COPD.^160^ Differentially methylated sites have not been previously reported in *JADE1*. |

**Table S3 (continued).** Gene functions and related diseases of identified differentially methylated sites (DMSs) associated with respiratory function traits or chronic obstructive pulmonary disease (COPD) in the Generation Scotland Scottish Family Health Study (GS:SFHS) discovery data. The gene information was obtained from GeneCards and systematic term (gene name with “DNA methylation”, “lung”, “COPD”, “asthma” or “lung cancer”) search in PubMed.

| **Gene (DMSs)** | **Gene function and related diseases** |
| --- | --- |
| ***JAK3***  **(**cg02370334**)** | *JAK3* (Janus Kinase 3) is a member of the Janus kinase (JAK) family of tyrosine kinases involved in cytokine receptor-mediated intracellular signal transduction.^121^ Mutations in *JAK3* have been linked to non-small cell lung cancer.^161,162^ JAK3 has also been shown to reduced airway inflammation in a murine model of allergic asthma.^163^ A disease associated with this gene is Jak3-deficient severe combined immunodeficiency.^164^ SNP in *JAK3* has been shown to associate with COPD.^165^ Differential methylation in *JAK3* has been reported to be associated with breast^166^ and bladder^167^ cancer, and [Yisui Shenxu Granule β-thalassemia therapy](https://www.sciencedirect.com/science/article/pii/S0378874108005084).^168^ |
| ***LOC100996291***  **(**cg19748455**)** | *LOC100996291* (LINC01993) is a long intergenic non-protein coding RNA.^121^ Little is known about the role of *LOC100996291* in disease. It has however been reported that differential methylation in *LOC100996291* is associated with Epstein-Barr-virus infected Burkitt lymphoma cell lines^169^ and Crohn’s disease.^170^ |
| ***OFCC1***  **(**cg00213822**)** | The function of OFCC1 (Orofacial Cleft 1 Candidate 1) is unclear, but it has been suggested that it is an interacting partner and methylation substrate of protein arginine methyl transferase 1.^171^ Diseases associated with *OFCC1* include orofacial cleft,^172^ and neuropsychiatric disorders: schizophrenia,^173^ obsessive-compulsive disorder,^174^ Tourette’s disorder^175^ and autism.^176^ Differentially methylated sites have not been previously reported in *OFCC1*. |
| ***PIK3R5***  **(**cg07687574**)** | *PIK3R5* (Phosphoinositide-3-Kinase Regulatory Subunit 5) plays important roles in cell growth, proliferation, differentiation, motility, survival and intracellular trafficking.^121^ A disease associated with *PIK3R5* is ataxia-oculomotor apraxia-3.^177^ Differential methylation in *PIK3R5* has been previously reported to be associated with non-small cell lung cancer^178^ and oral squamous cell carcinoma.^179^ |
| ***RALGDS***  **(**cg03770138**)** | *RALGDS* (Ral Guanine Nucleotide Dissociation Stimulator) is an effector of Ras-related GTPases.^121^ IL-6 can upregulate *RALGDS* in leukemic cells.^180^ *RALGDS* has also been shown to mediate transformed cells survival via the JNK/SAPK pathway and is essential for skin tumorigenesis in mice.^181^ Differential methylation in *RALGDS* has been reported to be associated with mouse liver tumorigenesis following phenobarbital treatment.^182^ |
| ***SBNO2***  **(**cg18608055**)** | *SBNO2* (Strawberry Notch Homolog 2) is a transcriptional co-regulator that plays a role in regulation of the pro-inflammatory cascade.^183^ Differential methylation in *SBNO2* has been previously reported to be associated with type 2 diabetes,^184^ Alzheimer’s^185^ and cardiovascular disease.^186^ |
| ***SF3B1***  **(**cg09455379**)** | *SF3B1* (Splicing Factor 3b Subunit 1) encodes an essential spliceosomal protein.^187^ Mutations in the SF3B1 gene have been linked to myelodysplastic syndrome^188^ as well as some other solid tumors.^189^ Furthermore, silencing of the splicing factor *SF3B1* has been shown to be effective in killing non-small cell lung cancer cells.^190^ |
| ***SIAH2***  **(**cg16963852**)** | *SIAH2* (Siah E3 Ubiquitin Protein Ligase 2) encodes a protein that is a member of the seven in absentia homolog (SIAH) family. The protein is an E3 ligase and is involved in ubiquitination and proteasome-mediated degradation of specific proteins. *SIAH2* upregulation mediates the ubiquitination of *NRF2*^191^ which has been previously associated with respiratory function^192^ and COPD.^193^ Expression of *SIAH2* is increased in lung cancer.^194^ Differentially methylated sites have not been previously reported in *SIAH2*. |
| ***SLAMF7***  **(**cg00045592**)** | *SLAMF7* (Signaling Lymphocytic Activation Molecule F7) a glycoprotein expressed on natural killer and multiple myeloma cells.^195^ Respiratory disease has been reported in multiple myeloma patients receiving elotuzumab, an immunostimulatory antibody that targets *SLAMF7*.^196^ Other diseases associated with *SLAMF7* include systemic lupus erythematosus^197^, IgG_4_-related disease^198^ and multiple myeloma.^199^ Differential methylation in *SLAMF7* has been reported to be associated with ageing^200^ and atherosclerosis.^201,202^ |
| ***SNTB1***  **(**cg01198738**)** | *SNTB1* (Syntrophin Beta 1) encodes a peripheral membrane protein found associated with dystrophin and dystrophin-related proteins.^121^ *SNTB1* underexpression has been associated with survival in lung adenocarcinoma.^203^ Diseases associated with *SNTB1* include Duchenne muscular dystrophy^204^ and myopia.^205^ A SNP near SNTB1 has been associated with HDL levels.^206^ EWAS have reported that differential methylation in *SNTB1* is associated with smoking^207^ and ovarian cancer.^208^ |
| ***SOCS3***  **(**cg18181703,  cg11047325,  cg13343932**)** | *SOCS3* (Suppressor Of Cytokine Signalling 3) encodes a suppressor of cytokine signalling.^121^ SOCS3 regulates the lung inflammatory response.^209^ Diseases associated with *SOCS3* include COPD,^210^ asthma,^211,212^ non-small cell lung^213^ and breast cancer.^214^ *SOCS3* gene expression is up-regulated in lung injury,^215^ and following curcumin treatment of lung squamous cell carcinoma cell line.^216^ SOCS3-knockout mice have more severe acute lung injury after lipopolysaccharide treatment.^215^ SNPs in this gene show genome-wide association with infantile asthma^217^ and creatinine kinase levels.^218^ EWAS have reported that differential methylation in *SOCS3* is associated with non-small cell lung cancer,^219^ hearing loss,^220^ metabolic syndrome,^221^ BMI,^222^ type 2 diabetes,^8,102,137^ metabolic diseases,^138^ and endometrial^223^ and prostate cancer.^224^ |
| ***UACA***  **(**cg18007249**)** | *UACA* (Uveal Autoantigen With Coiled-Coil Domains And Ankyrin Repeats) plays an important role in the regulation of stress-induced apoptosis.^121^ Diseases associated with *UACA* include graves' disease^225^ and non-small cell lung carcinoma.^226^ SNPs in this gene have been associated with respiratory function.^217^ An EWAS reported that differential methylation in *UACA* is associated with systemic lupus erythematosus.^227^ |

|  |  | **Epigenome-wide association studies (EWAS)** | | | | | | | | | **Meta-analysis** | | **DMSs_es_** |
| --- | --- | --- | --- | --- | --- | --- | --- | --- | --- | --- | --- | --- | --- |
|  |  |  |  |  |  | **β**  **(p-value)** |  |  |  |  | **β**  **(p-value)** | **Q**  **(p-value)** | **p-value** |
| **Trait** | **CpG site** | **All** | **>40** | **NS** | **NS>40** | **S** | **Sg40** | **S>10py** | **S>10py>40** | **NRS** |  |  |  |
| FEV_1_ | cg13993467 | -0.023 (5.19E-10) | -0.016 (6.85E-05) | -0.030 (4.49E-09) | -0.021 (1.41E-04) | -0.018 (5.50E-04) | -0.012 (2.56E-02) | -0.007 (3.59E-01) | -0.006 (4.90E-01) | -0.023 (7.07E-07) | -0.019 (9.81E-17) | 14.16 (7.77E-02) | 6.31E-01 |
|  | cg16963852 | 0.015 (2.88E-08) | 0.015 (1.62E-06) | 0.013 (6.28E-04) | 0.013 (1.80E-03) | 0.017 (1.91E-05) | 0.016 (4.26E-04) | 0.017 (6.38E-03) | 0.017 (8.63E-03) | 0.014 (2.07E-05) | 0.015 (7.66E-32) | 1.07 (9.98E-01) | 1.36E-07 |
|  | cg26804423 | -0.011 (4.88E-11) | -0.010 (2.91E-08) | -0.009 (1.25E-04) | -0.009 (6.70E-04) | -0.012 (5.82E-07) | -0.013 (7.70E-06) | -0.014 (1.92E-04) | -0.013 (1.05E-03) | -0.011 (9.10E-08) | -0.011 (1.62E-44) | 3.76 (8.78E-01) | 3.44E-02 |
|  | cg26080684 | -0.010 (9.75E-09) | -0.011 (1.20E-07) | -0.01 (5.33E-05) | -0.010 (4.63E-04) | -0.010 (9.45E-05) | -0.011 (1.05E-04) | -0.013 (1.62E-03) | -0.013 (1.68E-03) | -0.010 (4.35E-06) | -0.010 (8.90E-37) | 1.01 (9.98E-01) | **5.85E-09** |
|  | cg01198738 | 0.013 (5.12E-09) | 0.014 (3.53E-08) | 0.013 (2.56E-05) | 0.014 (4.24E-05) | 0.013 (1.02E-04) | 0.014 (2.02E-04) | 0.017 (8.13E-04) | 0.018 (8.66E-04) | 0.012 (1.49E-05) | 0.013 (1.94E-38) | 1.76 (9.87E-01) | 1.42E-03 |
|  | cg03770138 | 0.010 (2.34E-08) | 0.012 (9.19E-09) | 0.008 (1.40E-03) | 0.010 (4.63E-04) | 0.012 (1.02E-05) | 0.013 (3.94E-05) | 0.017 (6.20E-05) | 0.017 (2.60E-04) | 0.008 (5.08E-04) | 0.011 (9.99E-36) | 7.59 (4.74E-01) | **1.32E-15** |
|  | cg18871648 | 0.014 (4.30E-09) | 0.015 (9.41E-08) | 0.014 (1.36E-05) | 0.014 (3.26E-04) | 0.013 (2.72E-04) | 0.016 (2.00E-04) | 0.018 (2.73E-03) | 0.021 (9.58E-04) | 0.012 (8.45E-05) | 0.014 (9.23E-36) | 2.20 (9.74E-01) | **1.10E-59** |
|  | cg10919522 | 0.014 (2.14E-10) | 0.015 (1.55E-08) | 0.015 (5.26E-07) | 0.013 (2.24E-04) | 0.012 (3.21E-04) | 0.015 (1.04E-04) | 0.015 (6.45E-03) | 0.015 (8.73E-03) | 0.010 (3.15E-04) | 0.014 (1.02E-37) | 2.51 (9.61E-01) | **1.14E-47** |
|  | cg09018739 | -0.008 (5.49E-10) | -0.009 (1.74E-08) | -0.007 (4.76E-05) | -0.009 (8.89E-05) | -0.008 (1.46E-05) | -0.009 (9.78E-05) | -0.009 (4.93E-03) | -0.009 (8.33E-03) | -0.008 (5.65E-06) | -0.008 (2.79E-39) | 0.55 (1.00e+00) | **8.97E-14** |
|  | cg07687574 | 0.012 (6.68E-11) | 0.012 (2.31E-07) | 0.011 (1.12E-05) | 0.010 (1.81E-03) | 0.013 (2.76E-06) | 0.013 (4.44E-05) | 0.017 (1.71E-04) | 0.017 (4.70E-04) | 0.011 (6.89E-06) | 0.012 (2.79E-41) | 3.59 (8.92E-01) | **3.15E-27** |
|  | cg19748455 | 0.015 (1.11E-09) | 0.017 (1.98E-09) | 0.011 (1.80E-03) | 0.013 (5.84E-04) | 0.019 (5.18E-07) | 0.020 (2.27E-06) | 0.024 (5.94E-05) | 0.023 (2.46E-04) | 0.016 (5.06E-07) | 0.016 (2.39E-43) | 7.66 (4.68E-01) | 5.57E-04 |
|  | cg18181703 | 0.011 (4.51E-09) | 0.011 (7.27E-07) | 0.008 (1.35E-03) | 0.010 (1.50E-03) | 0.014 (2.53E-07) | 0.012 (1.16E-04) | 0.013 (2.55E-03) | 0.012 (9.97E-03) | 0.008 (1.33E-03) | 0.011 (6.24E-33) | 4.63 (7.97E-01) | **7.38E-14** |
|  | cg11047325 | 0.018 (3.91E-11) | 0.017 (1.55E-07) | 0.012 (1.34E-03) | 0.014 (2.58E-03) | 0.025 (2.15E-09) | 0.021 (1.60E-05) | 0.021 (1.67E-03) | 0.020 (3.87E-03) | 0.013 (2.27E-04) | 0.017 (4.22E-37) | 8.12 (4.22E-01) | **7.54E-22** |
|  | cg13343932 | 0.014 (6.52E-10) | 0.012 (1.97E-06) | 0.010 (1.47E-03) | 0.01 (4.13E-03) | 0.018 (4.57E-08) | 0.015 (9.26E-05) | 0.016 (2.96E-03) | 0.015 (9.77E-03) | 0.011 (1.40E-04) | 0.013 (2.17E-34) | 5.59 (6.93E-01) | **8.89E-25** |
|  | cg18608055 | 0.010 (5.52E-09) | 0.010 (7.27E-07) | 0.008 (5.86E-04) | 0.008 (1.93E-03) | 0.013 (8.59E-07) | 0.012 (7.01E-05) | 0.012 (4.03E-03) | 0.013 (3.02E-03) | 0.009 (2.11E-05) | 0.01 (4.85E-35) | 3.40 (9.07E-01) | 1.15E-04 |

**Table S5.** Summary of results from the discovery (all), older adult (> 40 years), smokers with a substantial smoking history, individuals with non-restrictive spirometry pattern, and stratified smoking status and overall meta-analyses for differentially methylated sites (DMSs) reaching genome-wide significance in the discovery data, ordered by chromosomal location.

**Key:** **Chr**., chromosome, **β**, regression coefficient, **DMSs_es_**_,_ genome-wide significant differentially methylated sites associated with ever smoked in the Generation Scotland: Scottish Family Health Survey (GS:SFHS) cohort are highlighted in bold; **FEV_1_**_;_ Forced expiratory volume in one second; **FVC**, forced vital capacity; **all**, all data; **>40**, data from participants aged 40 or greater; **NS**, data from non-smokers; **S**, data from smokers; **>10py**, data from participants with a smoking history of 10 pack years or greater,  **NRS,** data from participants with non-restrictive spirometry pattern, **Q**, the Cochran's Q test statistic used to assess heterogeneity of effect-size estimates from the individual datasets. Non-robust (heterogeneous) associations are highlighted in bold.

**Table S5 (continued).** Summary of results from the discovery (all), older adult (> 40 years), smokers with a substantial smoking history, individuals with non-restrictive spirometry pattern, and stratified smoking status and overall meta-analyses for differentially methylated sites (DMSs) reaching genome-wide significance in the discovery data, ordered by chromosomal location.

|  |  | **Epigenome-wide association studies (EWAS)** | | | | | | | | | **Meta-analysis** | | **DMSs_es_** |
| --- | --- | --- | --- | --- | --- | --- | --- | --- | --- | --- | --- | --- | --- |
|  |  |  |  |  |  | **β**  **(p-value)** |  |  |  |  | **β**  **(p-value)** | **Q**  **(p-value)** | **p-value** |
| **Trait** | **CpG site** | **All** | **>40** | **NS** | **NS>40** | **S** | **Sg40** | **S>10py** | **S>10py>40** | **NRS** |  |  |  |
| FEV_1_ | cg02370334 | 0.011 (3.48E-08) | 0.012 (5.25E-07) | 0.007 (9.37E-03) | 0.007 (2.33E-02) | 0.015 (1.21E-07) | 0.016 (9.14E-07) | 0.017 (2.48E-04) | 0.019 (1.47E-04) | 0.009 (1.79E-04) | 0.012 (2.79E-23) | 11.94 (1.54E-01) | 1.76E-01 |
|  | cg03187361 | 0.011 (1.39E-08) | 0.012 (5.70E-07) | 0.012 (2.90E-05) | 0.011 (5.88E-04) | 0.011 (2.91E-04) | 0.011 (1.83E-03) | 0.014 (5.03E-03) | 0.015 (3.79E-03) | 0.011 (3.62E-05) | 0.011 (7.82E-33) | 0.85 (9.99E-01) | 1.49E-05 |
| **FVC** | cg13993467 | -0.025 (1.17E-11) | -0.012 (1.65E-03) | -0.028 (6.45E-08) | -0.015 (6.53E-03) | -0.023 (9.90E-06) | -0.011 (5.68E-02) | -0.010 (1.90E-01) | -0.007 (3.92E-01) | -0.026 (4.24E-10) | -0.018 (2.56E-12) | 19.16 (1.40E-02) | 6.31E-01 |
|  | cg01620970 | -0.012 (4.11E-09) | -0.011 (1.41E-05) | -0.011 (9.66E-05) | -0.007 (3.27E-02) | -0.014 (1.59E-05) | -0.013 (2.07E-04) | -0.007 (1.20E-01) | -0.009 (7.64E-02) | -0.013 (9.46E-08) | -0.011 (1.61E-31) | 3.83 (8.72E-01) | **1.71E-08** |
|  | **cg00213822** | **-0.02 (5.23E-11)** | **-0.012 (2.03E-04)** | **-0.029 (9.09E-11)** | **-0.020 (2.66E-05)** | **-0.010 (2.98E-02)** | **-0.003 (4.98E-01)** | **-0.002 (7.67E-01)** | **0.000 (9.72E-01)** | **-0.021 (5.16E-09)** | **-0.014 (2.52E-05)** | **34.91 (2.78E-05)** | 4.74E-02 |
|  | cg15659943 | -0.009 (1.64E-08) | -0.011 (4.88E-09) | -0.011 (1.34E-06) | -0.013 (1.19E-06) | -0.008 (1.16E-03) | -0.010 (3.99E-04) | -0.011 (1.34E-03) | -0.011 (4.80E-03) | -0.008 (1.45E-05) | -0.010 (6.39E-39) | 3.88 (8.67E-01) | **9.96E-09** |
|  | cg25465557 | -0.013 (2.11E-10) | -0.005 (3.97E-02) | -0.017 (1.19E-08) | -0.005 (1.04E-01) | -0.010 (1.81E-03) | -0.004 (1.68E-01) | -0.007 (1.12E-01) | -0.006 (1.76E-01) | -0.014 (2.81E-09) | -0.009 (2.64E-08) | 23.82 (2.46E-03) | 2.78E-06 |
|  | cg18007249 | -0.013 (5.60E-10) | -0.007 (4.16E-03) | -0.014 (1.16E-06) | -0.005 (1.17E-01) | -0.012 (1.41E-04) | -0.008 (2.14E-02) | -0.009 (4.43E-02) | -0.008 (8.99E-02) | -0.010 (2.99E-05) | -0.010 (1.17E-18) | 8.57 (3.80E-01) | 8.49E-01 |
|  | **cg13108341** | **-0.032 (2.57E-08)** | **-0.002 (6.73E-01)** | **-0.034 (2.62E-05)** | **-0.001 (8.74E-01)** | **-0.030 (3.21E-04)** | **-0.006 (4.63E-01)** | **-0.013 (2.30E-01)** | **-0.005 (6.93E-01)** | **-0.034 (3.49E-07)** | **-0.018 (3.60E-04)** | **32.94 (6.30E-05)** | 7.91E-02 |
| **FEV_1_/ FVC** | cg00045592 | 0.014 (1.22E-08) | 0.015 (1.93E-07) | 0.006 (4.04E-02) | 0.005 (1.28E-01) | 0.020 (2.15E-06) | 0.022 (5.81E-06) | 0.018 (1.97E-03) | 0.019 (1.78E-03) | 0.014 (7.12E-07) | 0.014 (2.01E-12) | 20.80 (7.70E-03) | **7.91E-255** |
|  | **cg05575921** | **0.029 (3.48E-09)** | **0.032 (6.24E-09)** | **0.000 (9.41E-01)** | **0.001 (7.89E-01)** | **0.050 (1.47E-08)** | **0.052 (3.42E-08)** | **0.047 (3.02E-05)** | **0.046 (9.40E-05)** | **0.034 (1.62E-09)** | **0.031 (4.32E-06)** | **76.00 (3.12E-13)** | **0.00E+00** |
|  | **cg03636183** | **0.016 (5.10E-09)** | **0.018 (9.16E-09)** | **0.000 (9.73E-01)** | **0.003 (3.48E-01)** | **0.029 (2.69E-09)** | **0.030 (7.11E-08)** | **0.031 (1.07E-05)** | **0.031 (2.32E-05)** | **0.017 (6.13E-08)** | **0.018 (3.50E-06)** | **74.49 (6.24E-13)** | **0.00E+00** |
| COPD | cg09455379 | -0.030 (3.28E-08) | -0.032 (2.08E-07) | -0.021 (7.98E-03) | -0.023 (9.60E-03) | -0.038 (5.17E-07) | -0.044 (4.97E-07) | -0.053 (5.31E-07) | -0.056 (1.03E-06) | -0.027 (9.42E-07) | -0.034 (2.46E-23) | 14.82 (6.28E-02) | 4.16E-01 |
|  | cg20453862 | -0.039 (7.42E-09) | -0.048 (3.20E-09) | -0.055 (3.13E-08) | -0.058 (5.15E-07) | -0.031 (1.63E-03) | -0.042 (2.83E-04) | -0.019 (1.89E-01) | -0.024 (1.19E-01) | -0.033 (3.21E-06) | -0.040 (8.20E-29) | 11.04 (1.99E-01) | 3.18E-01 |

**Key:** **Chr**., chromosome, **β**, regression coefficient, **DMSs_es_**_,_ genome-wide significant differentially methylated sites associated with ever smoked in the Generation Scotland: Scottish Family Health Survey (GS:SFHS) cohort are highlighted in bold; **FEV_1_**_;_ Forced expiratory volume in one second; **FVC**, forced vital capacity; **all**, all data; **>40**, data from participants aged 40 or greater; **NS**, data from non-smokers; **S**, data from smokers; **>10py**, data from participants with a smoking history of 10 pack years or greater,  **NRS,** data from participants with non-restrictive spirometry pattern, **Q**, the Cochran's Q test statistic used to assess heterogeneity of effect-size estimates from the individual datasets. Non-robust (heterogeneous) associations are highlighted in bold.

**Table S6.** Summary of epigenome-wide association study results for the three respiratory function traits and COPD with and without pre-correction for smoking history in the discovery (all), older adult (> 40 years), smokers with a substantial smoking history, individuals with non-restrictive spirometry pattern, and stratified smoking status datasets from the discovery Generation Scotland: Scottish Family health study (GS:SFHS) cohort.

|  | **FEV_1_** | | **FVC** | | **FEV_1_/FVC** | | **COPD** | |
| --- | --- | --- | --- | --- | --- | --- | --- | --- |
| **Pre-correction** | **Yes** | **No** | **Yes** | **No** | **Yes** | **No** | **Yes** | **No** |
| **N_all_** | 3781 | | | | | | 3193 | |
| **λ_all_** | 1.09 | 1.08 | 1.08 | 1.08 | 1.04 | 1.04 | 1.12 | 1.11 |
| **DMSs_all_** | 17 | 16 | 7 | 8 | 3 | 3 | 2 | 1 |
| **DMSs_all-smoking_** | 9 | 9 | 2 | 3 | 3 | 3 | 0 | 0 |
| **N_>40_** | 2853 | | | | | | 2352 | |
| **λ_>40_** | 1.05 | 1.05 | 1.05 | 1.05 | 1.04 | 1.04 | 1.10 | 1.11 |
| **DMSs_>40_** | 6 | 5 | 1 | 2 | 2 | 2 | 2 | 2 |
| **DMSs_>40-smoking_** | 3 | 3 | 1 | 2 | 2 | 2 | 0 | 0 |
| **N_non-smokers_** | 1948 | | | | | | 1699 | |
| **λ_non-smokers_** | 1.06 | 1.06 | 1.06 | 1.07 | 1.01 | 1.01 | 1.11 | 1.11 |
| **DMSs_non-smokers_** | 1 | 1 | 2 | 2 | 0 | 0 | 3 | 3 |
| **DMSs_non-smokers-smoking_** | 0 | 0 | 0 | 0 | 0 | 0 | 0 | 0 |
| **N_non-smokers >40_** | 1436 | | | | | | 1236 | |
| **λ_non-smokers >40_** | 1.04 | 1.04 | 1.04 | 1.04 | 1.02 | 1.02 | 1.09 | 1.09 |
| **DMSs_non-smokers >40_** | 0 | 0 | 0 | 0 | 0 | 0 | 3 | 3 |
| **DMSs_non-smokers >40-smoking_** | 0 | 0 | 0 | 0 | 0 | 0 | 0 | 0 |
| **N_smokers_** | 1729 | | | | | | 1412 | |
| **λ_smokers_** | 1.03 | 1.03 | 1.01 | 1.01 | 1.02 | 1.02 | 1.09 | 1.07 |
| **DMSs_smokers_** | 1 | 1 | 0 | 0 | 2 | 1 | 2 | 1 |
| **DMSs_smokers-smoking_** | 1 | 1 | 0 | 0 | 2 | 1 | 0 | 0 |
| **N_smokers >40_** | 1340 | | | | | | 1058 | |
| **λ_smokers >40_** | 1.02 | 1.01 | 1.00 | 0.99 | 1.01 | 1.01 | 1.07 | 1.06 |
| **DMSs_smokers >40_** | 2 | 0 | 0 | 0 | 1 | 0 | 0 | 1 |
| **DMSs_smokers >40-smoking_** | 2 | 0 | 0 | 0 | 1 | 0 | 0 | 0 |
| **N_smokers >10py_** | 936 | | | | | | 726 | |
| **λ_smokers >10py_** | 1 | 1 | 0.98 | 0.98 | 0.99 | 0.99 | 1.03 | 1.05 |
| **DMSs_smokers >10py_** | 0 | 0 | 0 | 0 | 0 | 0 | 0 | 0 |
| **DMSs_smokers >10py-smoking_** | 0 | 0 | 0 | 0 | 0 | 0 | 0 | 0 |
| **N_smokers >10py >40_** | 829 | | | | | | 634 | |
| **λ_smokers >10py >40_** | 1.01 | 1.01 | 0.99 | 0.99 | 0.99 | 0.99 | 1.02 | 1.03 |
| **DMSs_smokers >10py >40_** | 0 | 0 | 0 | 0 | 0 | 0 | 0 | 0 |
| **DMSs_smokers >10py >40-smoking_** | 0 | 0 | 0 | 0 | 0 | 0 | 0 | 0 |
| **N_NRS_** | 3418 | | | | | | 3092 | |
| **λ_NRS_** | 1.02 | 1.02 | 1.06 | 1.06 | 1.02 | 1.02 | 1.09 | 1.09 |
| **DMSs_NRS_** | 1 | 1 | 3 | 3 | 1 | 1 | 0 | 1 |
| **DMSs_NRS-smoking_** | 1 | 1 | 0 | 0 | 1 | 1 | 0 | 0 |

**Key:** **N**, sample size; **all**, all data; **λ**, genomic inflation factor; **DMSs**, genome-wide significant differentially-methylated CpG sites; **smoking**, DMSs that were also genome-wide significant in an EWAS for ever-smoked in the GS:SFHS cohort; **>40**, data from participants aged 40 or greater, **non-smokers**, data from non-smokers; **smokers**, data from smokers, **>10py**, data from participants with a smoking history of 10 pack years or greater, **NRS,** data from participants with non-restrictive spirometry pattern.

**Table S7.** Summary of results from the discovery (all), older adult (> 40 years), smokers with a substantial smoking history, individuals with non-restrictive spirometry pattern, and stratified smoking status datasets and overall meta-analyses for differentially methylated sites (DMSs) reaching genome-wide significance in the discovery data when the respiratory function traits or chronic obstructive pulmonary disease (COPD) were not pre-corrected for smoking history, ordered by chromosomal location.

|  |  | **Epigenome-wide association studies (EWAS)** | | | | | | | | | **Meta-analysis** | | **DMSs_es_** |  |
| --- | --- | --- | --- | --- | --- | --- | --- | --- | --- | --- | --- | --- | --- | --- |
|  |  |  |  |  |  | **β**  **(p-value)** |  |  |  |  | **β**  **(p-value)** | **Q**  **(p-value)** | **p-value** |  |
| **Trait** | **CpG site** | **All** | **>40** | **NS** | **NS>40** | **S** | **Sg40** | **S>10py** | **S>10py>40** | **NRS** |  |  |  |  |
| FEV_1_ | cg13993467 | -0.023 (5.03E-10) | -0.016 (6.19E-05) | -0.030 (4.98E-09) | -0.021 (1.48E-04) | -0.019 (6.38E-04) | -0.012 (3.24E-02) | -0.007 (3.60E-01) | -0.005 (5.03E-01) | -0.023 (6.52E-07) | -0.019 (3.94E-17) | 13.84 (8.61E-02) | 6.31E-01 |  |
|  | cg16963852 | 0.015 (3.37E-08) | 0.015 (1.55E-06) | 0.012 (6.57E-04) | 0.013 (1.75E-03) | 0.017 (2.11E-05) | 0.017 (3.82E-04) | 0.017 (6.75E-03) | 0.017 (8.60E-03) | 0.014 (2.14E-05) | 0.015 (1.07E-31) | 1.33 (9.95E-01) | 1.36E-07 |  |
|  | cg26804423 | -0.011 (6.12E-11) | -0.011 (3.33E-08) | -0.008 (1.26E-04) | -0.009 (7.03E-04) | -0.013 (5.41E-07) | -0.013 (6.69E-06) | -0.014 (1.99E-04) | -0.013 (9.80E-04) | -0.011 (8.57E-08) | -0.011 (2.47E-44) | 4.37 (8.23E-01) | 3.44E-02 |  |
|  | cg26080684 | -0.01 (1.21E-08) | -0.011 (1.82E-07) | -0.01 (5.41E-05) | -0.010 (5.01E-04) | -0.010 (9.76E-05) | -0.012 (1.28E-04) | -0.013 (1.60E-03) | -0.013 (1.61E-03) | -0.01 (4.70E-06) | -0.011 (2.45E-36) | 1.11 (9.97E-01) | 5.85E-09 |  |
|  | cg01198738 | 0.013 (5.35E-09) | 0.014 (3.97E-08) | 0.013 (2.64E-05) | 0.014 (4.02E-05) | 0.013 (1.26E-04) | 0.014 (2.08E-04) | 0.017 (8.07E-04) | 0.018 (9.99E-04) | 0.012 (1.53E-05) | 0.014 (3.17E-38) | 1.73 (9.88E-01) | 1.42E-03 |  |
|  | cg03770138 | 0.010 (2.48E-08) | 0.012 (1.10E-08) | 0.008 (1.41E-03) | 0.010 (4.52E-04) | 0.012 (1.12E-05) | 0.013 (3.68E-05) | 0.017 (6.71E-05) | 0.017 (2.76E-04) | 0.008 (5.18E-04) | 0.011 (1.10E-34) | 7.95 (4.38E-01) | **1.32E-15** |  |
|  | cg18871648 | 0.015 (3.70E-09) | 0.015 (8.94E-08) | 0.014 (1.31E-05) | 0.014 (2.94E-04) | 0.014 (2.81E-04) | 0.016 (2.23E-04) | 0.018 (2.74E-03) | 0.021 (9.24E-04) | 0.012 (8.62E-05) | 0.015 (8.06E-36) | 2.33 (9.69E-01) | **1.10E-59** |  |
|  | cg10919522 | 0.015 (2.04E-10) | 0.015 (1.45E-08) | 0.015 (5.47E-07) | 0.013 (2.11E-04) | 0.013 (3.21E-04) | 0.016 (1.22E-04) | 0.015 (6.53E-03) | 0.015 (8.31E-03) | 0.019 (3.20E-04) | 0.014 (1.12E-37) | 2.68 (9.53E-01) | **1.14E-47** |  |
|  | cg09018739 | -0.008 (6.21E-10) | -0.009 (1.75E-08) | -0.007 (4.90E-05) | -0.008 (8.89E-05) | -0.009 (1.40E-05) | -0.009 (9.71E-05) | -0.009 (5.00E-03) | -0.009 (8.29E-03) | -0.008 (5.45E-06) | -0.008 (3.36E-39) | 0.76 (9.99E-01) | **8.97E-14** |  |
|  | cg07687574 | 0.013 (7.28E-11) | 0.012 (3.07E-07) | 0.011 (1.09E-05) | 0.010 (1.77E-03) | 0.014 (3.09E-06) | 0.014 (3.88E-05) | 0.017 (1.63E-04) | 0.017 (5.20E-04) | 0.011 (7.02E-06) | 0.012 (4.70E-41) | 3.89 (8.67E-01) | **3.15E-27** |  |
|  | cg19748455 | 0.015 (1.15E-09) | 0.017 (2.00E-09) | 0.011 (1.82E-03) | 0.013 (5.39E-04) | 0.019 (5.15E-07) | 0.021 (1.66E-06) | 0.024 (5.33E-05) | 0.024 (2.25E-04) | 0.016 (4.88E-07) | 0.016 (5.87E-39) | 8.51 (3.85E-01) | 5.57E-04 |  |
|  | cg18181703 | 0.011 (5.91E-09) | 0.011 (1.02E-06) | 0.008 (1.31E-03) | 0.010 (1.40E-03) | 0.014 (3.34E-07) | 0.013 (1.29E-04) | 0.013 (2.64E-03) | 0.012 (1.00E-02) | 0.008 (1.36E-03) | 0.011 (1.91E-32) | 4.97 (7.60E-01) | 7.38E-14 |  |
|  | cg11047325 | 0.019 (5.40E-11) | 0.017 (2.01E-07) | 0.012 (1.26E-03) | 0.014 (2.38E-03) | 0.025 (2.52E-09) | 0.021 (1.99E-05) | 0.021 (1.68E-03) | 0.020 (4.12E-03) | 0.013 (2.25E-04) | 0.017 (2.04E-34) | 8.69 (3.70E-01) | **7.54E-22** |  |

**Key:** **Chr**., chromosome, **β**, regression coefficient, **DMSs_es_**_,_ genome-wide significant differentially methylated sites associated with ever smoked in the Generation Scotland: Scottish Family Health Survey (GS:SFHS) cohort are highlighted in bold; **FEV_1_**_;_ Forced expiratory volume in one second; **FVC**, forced vital capacity; **all**, all data; **>40**, data from participants aged 40 or greater; **NS**, data from non-smokers; **S**, data from smokers; **>10py**, data from participants with a smoking history of 10 pack years or greater,  **NRS,** data from participants with non-restrictive spirometry pattern, **Q**, the Cochran's Q test statistic used to assess heterogeneity of effect-size estimates from the individual datasets. Non-robust (heterogeneous) associations are highlighted in bold.

|  |  | **Epigenome-wide association studies (EWAS)** | | | | | | | | | **Meta-analysis** | | **DMSs_es_** |
| --- | --- | --- | --- | --- | --- | --- | --- | --- | --- | --- | --- | --- | --- |
|  |  | **β**  **(p-value)** | | | | | | | | | **β**  **(p-value)** | **Q**  **(p-value)** | **p-value** |
| **Trait** | **CpG site** | **All** | **>40** | **NS** | **NS>40** | **S** | **Sg40** | **S>10py** | **S>10py>40** | **NRS** |  |  |  |
| FEV_1_ | cg13343932 | 0.014 (8.57E-10) | 0.012 (2.30E-06) | 0.010 (1.38E-03) | 0.010 (3.84E-03) | 0.018 (5.60E-08) | 0.015 (1.02E-04) | 0.016 (3.00E-03) | 0.014 (1.04E-02) | 0.011 (1.37E-04) | 0.013 (5.06E-34) | 5.99 (6.49E-01) | **8.89E-25** |
|  | cg18608055 | 0.010 (8.58E-09) | 0.001 (1.14E-06) | 0.008 (5.67E-04) | 0.008 (1.75E-03) | 0.013 (1.16E-06) | 0.012 (8.24E-05) | 0.012 (3.99E-03) | 0.013 (2.69E-03) | 0.009 (2.19E-05) | 0.010 (1.76E-34) | 3.74 (8.80E-01) | 1.15E-04 |
|  | cg03187361 | 0.012 (1.26E-08) | 0.012 (4.68E-07) | 0.012 (2.91E-05) | 0.011 (5.93E-04) | 0.011 (3.47E-04) | 0.011 (1.91E-03) | 0.014 (5.00E-03) | 0.015 (3.86E-03) | 0.011 (3.37E-05) | 0.012 (6.86E-33) | 0.85 (9.99E-01) | 1.49E-05 |
| **FVC** | cg13993467 | -0.025 (1.21E-11) | -0.012 (1.49E-03) | -0.028 (6.54E-08) | -0.015 (6.55E-03) | -0.023 (1.22E-05) | -0.011 (4.87E-02) | -0.009 (1.99E-01) | -0.006 (4.00E-01) | -0.026 (5.69E-10) | -0.018 (1.10E-12) | 18.54 (1.75E-02) | 6.31E-01 |
|  | cg10919522 | 0.012 (3.47E-08) | 0.012 (4.12E-06) | 0.016 (2.46E-07) | 0.011 (1.09E-03) | 0.008 (2.00E-02) | 0.013 (1.87E-03) | 0.007 (1.67E-01) | 0.008 (1.27E-01) | 0.007 (6.94E-03) | 0.011 (5.74E-23) | 7.18 (5.17E-01) | **1.14E-47** |
|  | cg01620970 | -0.012 (4.08E-09) | -0.011 (1.27E-05) | -0.011 (1.02E-04) | -0.007 (3.27E-02) | -0.014 (1.39E-05) | -0.014 (1.85E-04) | -0.007 (1.26E-01) | -0.009 (8.19E-02) | -0.013 (9.50E-08) | -0.011 (1.59E-31) | 4.18 (8.41E-01) | **1.71E-08** |
|  | **cg00213822** | **-0.021 (5.08E-11)** | **-0.012 (2.11E-04)** | **-0.029 (7.89E-11)** | **-0.020 (2.57E-05)** | **-0.010 (2.86E-02)** | **-0.003 (4.82E-01)** | **-0.002 (7.62E-01)** | **0.000 (9.55E-01)** | **-0.021 (6.05E-09)** | **-0.014 (2.20E-05)** | **34.34 (3.52E-05)** | 4.74E-02 |
|  | cg15659943 | -0.009 (1.18E-08) | -0.011 (3.59E-09) | -0.011 (1.07E-06) | -0.013 (1.02E-06) | -0.008 (7.73E-04) | -0.01 (2.67E-04) | -0.012 (1.27E-03) | -0.011 (4.56E-03) | -0.008 (1.91E-05) | -0.010 (1.29E-39) | 3.89 (8.67E-01) | **9.96E-09** |
|  | cg25465557 | -0.013 (2.54E-10) | -0.005 (3.93E-02) | -0.017 (1.22E-08) | -0.005 (1.06E-01) | -0.010 (1.64E-03) | -0.005 (1.49E-01) | -0.007 (1.17E-01) | -0.006 (1.79E-01) | -0.014 (2.96E-09) | -0.009 (1.76E-08) | 23.23 (3.09E-03) | 2.78E-06 |
|  | cg18007249 | -0.013 (5.11E-10) | -0.007 (4.01E-03) | -0.014 (1.06E-06) | -0.005 (1.18E-01) | -0.012 (1.44E-04) | -0.008 (1.92E-02) | -0.009 (4.33E-02) | -0.009 (8.93E-02) | -0.010 (3.57E-05) | -0.010 (1.28E-18) | 8.57 (3.80E-01) | 8.49E-01 |
|  | **cg13108341** | **-0.032 (2.54E-08)** | **-0.003 (6.59E-01)** | **-0.034 (2.46E-05)** | **-0.001 (8.72E-01)** | **-0.031 (2.46E-04)** | **-0.007 (3.96E-01)** | **-0.013 (2.34E-01)** | **-0.004 (7.18E-01)** | **-0.034 (3.58E-07)** | **-0.018 (3.09E-04)** | **32.73 (6.90E-05)** | 7.91E-02 |
| **FEV_1_/ FVC** | cg00045592 | 0.014 (2.14E-08) | 0.015 (3.87E-07) | 0.006 (4.01E-02) | 0.005 (1.28E-01) | 0.020 (3.00E-06) | 0.022 (8.46E-06) | 0.018 (2.28E-03) | 0.019 (1.96E-03) | 0.014 (7.98E-07) | 0.014 (2.60E-12) | 20.52 (8.53E-03) | **7.91E-255** |
|  | **cg05575921** | **0.028 (1.28E-08)** | **0.031 (2.39E-08)** | **0.000 (9.49E-01)** | **0.001 (7.94E-01)** | **0.049 (3.76E-08)** | **0.052 (6.96E-08)** | **0.047 (3.32E-05)** | **0.046 (1.15E-04)** | **0.034 (2.30E-09)** | **0.031 (4.92E-06)** | **73.85 (8.39E-13)** | **0.00E+00** |
|  | **cg03636183** | **0.016 (1.29E-08)** | **0.018 (1.92E-08)** | **0.000 (9.65E-01)** | **0.003 (3.39E-01)** | **0.030 (4.08E-09)** | **0.030 (8.59E-08)** | **0.031 (1.13E-05)** | **0.031 (2.42E-05)** | **0.017 (7.50E-08)** | **0.018 (3.88E-06)** | **74.01 (7.79E-13)** | **0.00E+00** |
| COPD | cg20453862 | -0.047 (9.17E-11) | -0.056 (9.62E-11) | -0.055 (3.10E-08) | -0.057 (6.14E-07) | -0.035 (9.01E-04) | -0.046 (2.88E-04) | -0.011 (4.24E-01) | -0.018 (2.64E-01) | -0.030 (9.67E-06) | -0.041 (4.51E-17) | 17.33 (2.69E-02) | 3.18E-01 |

**Table S7 (continued).** Summary of results from the discovery (all), older adult (> 40 years), smokers with a substantial smoking history, individuals with non-restrictive spirometry pattern, and stratified smoking status datasets and overall meta-analyses for differentially methylated sites (DMSs) reaching genome-wide significance in the discovery data when the respiratory function traits or chronic obstructive pulmonary disease (COPD) were not pre-corrected for smoking history, ordered by chromosomal location.

**Key:** **Chr**., chromosome, **β**, regression coefficient, **DMSs_es_**_,_ genome-wide significant differentially methylated sites associated with ever smoked in the Generation Scotland: Scottish Family Health Survey (GS:SFHS) cohort are highlighted in bold; **FEV_1_**_;_ Forced expiratory volume in one second; **FVC**, forced vital capacity; **all**, all data; **>40**, data from participants aged 40 or greater; **NS**, data from non-smokers; **S**, data from smokers; **>10py**, data from participants with a smoking history of 10 pack years or greater,  **NRS,** data from participants with non-restrictive spirometry pattern, **Q**, the Cochran's Q test statistic used to assess heterogeneity of effect-size estimates from the individual datasets. Non-robust (heterogeneous) associations are highlighted in bold.

**Table S8.** Summary of results from the discovery, older adults (> 40 years), smokers with a substantial smoking history, individuals with non-restrictive spirometry pattern, and stratified smoking status datasets and overall meta-analyses for differentially methylated sites (DMSs) reaching genome-wide significance in the discovery when forced vital capacity (FVC) and chronic obstructive pulmonary disease (COPD) was, or was not pre-correction for smoking history.

|  |  |  | **Epigenome-wide association studies (EWAS)** | | | | | | | | | **Meta-analysis** | |
| --- | --- | --- | --- | --- | --- | --- | --- | --- | --- | --- | --- | --- | --- |
|  |  |  | **β**  **(p-value)** | | | | | | | | | **β**  **(p-value)** | **Q**  **(p-value)** |
| **Trait** | **Pre-correction** | **CpG site**  **(DMSs_es_ p-value_)_** | **All** | **>40** | **NS** | **NS>40** | **S** | **Sg40** | **S>10py** | **S>10py>40** | **NRS** |  |  |
| FEV_1_ | **Yes** | cg02370334 (1.76E-01) | 0.011 (3.48E-08) | 0.012 (5.25E-07) | 0.007 (9.37E-03) | 0.007 (2.33E-02) | 0.015 (1.21E-07) | 0.016 (9.14E-07) | 0.017 (2.48E-04) | 0.019 (1.47E-04) | 0.009 (1.79E-04) | 0.012 (2.79E-23) | 11.94 (1.54E-01) |
|  | **No** |  | 0.011 (4.89E-08) | 0.012 (7.27E-07) | 0.007 (9.49E-03) | 0.007 (2.29E-02) | 0.016 (1.05E-07) | 0.017 (1.07E-06) | 0.017 (2.47E-04) | 0.019 (1.42E-04) | 0.009 (1.81E-04) | 0.012 (3.27E-21) | 12.86 (1.17E-01) |
| FVC | Yes | cg10919522 (**1.14E-47)** | 0.012 (4.76E-08) | 0.012 (5.67E-06) | 0.016 (3.50E-07) | 0.011 (1.21E-03) | 0.008 (2.57E-02) | 0.012 (2.68E-03) | 0.007 (1.74E-01) | 0.008 (1.32E-01) | 0.007 (5.78E-03) | 0.011 (6.15E-24) | 6.86 (5.52E-01) |
|  | **No** |  | 0.012 (3.47E-08) | 0.012 (4.12E-06) | 0.016 (2.46E-07) | 0.011 (1.09E-03) | 0.008 (2.00E-02) | 0.013 (1.87E-03) | 0.007 (1.67E-01) | 0.008 (1.27E-01) | 0.007 (6.94E-03) | 0.011 (5.74E-23) | 7.18 (5.17E-01) |
| COPD | **Yes** | cg09455379 (4.16E-01) | -0.030 (3.28E-08) | -0.032 (2.08E-07) | -0.021 (7.98E-03) | -0.023 (9.60E-03) | -0.038 (5.17E-07) | -0.044 (4.97E-07) | -0.053 (5.31E-07) | -0.056 (1.03E-06) | -0.027 (9.42E-07) | -0.034 (2.46E-23) | 14.82 (6.28E-02) |
|  | No |  | -0.030 (1.17E-07) | -0.034 (5.47E-07) | -0.021 (7.87E-03) | -0.023 (9.38E-03) | -0.040 (1.04E-06) | -0.046 (2.16E-06) | -0.050 (2.89E-06) | -0.057 (1.24E-06) | -0.026 (1.24E-06) | -0.034 (1.16E-21) | 15.00 (5.91E-02) |

**Key:** **Chr**., chromosome, **β**, regression coefficient, **DMSs_es_**_,_ genome-wide significant differentially methylated sites associated with ever smoked in the Generation Scotland: Scottish Family Health Survey (GS:SFHS) cohort are highlighted in bold; **FEV_1_**_;_ Forced expiratory volume in one second; **FVC**, forced vital capacity; **all**, all data; **>40**, data from participants aged 40 or greater; **NS**, data from non-smokers; **S**, data from smokers; **Q**, the Cochran's Q test statistic used to assess heterogeneity of effect-size estimates from the individual datasets. Genome-wide significant DMSs from the analyses with (**Yes**) and without (**No**) pre-correction of the trait data are highlighted in bold.

**Table S9.** Canonical pathways enriched for genes with differentially methylated CpG sites associated with forced expiratory volume in one second (FEV_1_), forced vital capacity (FVC), FEV_1_/ FVC ratio and chronic obstructive pulmonary disease (COPD) in the discovery (n= 3,781) dataset identified by Ingenuity Pathway Analysis (IPA) (P_BH_< 0.05).

|  | **P_BH_-value^*^** | | | |
| --- | --- | --- | --- | --- |
| **Trait** | **FEV_1_** | **FVC** | **FEV_1_/FVC** | **COPD** |
| **Ingenuity canonical pathways** |  |  |  |  |
| **Group: Metabolic pathway** |  |  |  |  |
| 4-aminobutyrate Degradation I | 0.540 | - | - | 0.046 |
| Histidine Degradation III | 0.223 | - | 0.518 | 0.041 |
| Superpathway of Inositol Phosphate Compounds | 0.336 | 0.169 | 0.325 | 0.046 |
| tRNA Splicing | 0.542 | 0.026 | 0.698 | 0.426 |
| **Group: Signaling pathway** |  |  |  |  |
| **Category: Apoptosis Signaling:** |  |  |  |  |
| 14-3-3-mediated Signaling | 0.058 | 0.026 | 0.037 | 0.138 |
| LPS-stimulated MAPK Signaling | 0.065 | 0.049 | 0.113 | 0.682 |
| **Category: Cancer** |  |  |  |  |
| Acute Myeloid Leukemia Signaling | 0.006 | 0.059 | 0.178 | 0.195 |
| Basal Cell Carcinoma Signaling | 0.108 | 0.147 | 0.143 | 0.033 |
| Breast Cancer Regulation by Stathmin1 | 0.025 | 0.020 | 0.010 | 0.107 |
| Chronic Myeloid Leukemia Signaling | 0.017 | 0.107 | 0.192 | 0.377 |
| Colorectal Cancer Metastasis Signaling | 0.065 | 0.002 | 0.019 | 0.074 |
| Endocannabinoid Cancer Inhibition Pathway | 0.038 | 0.008 | 0.015 | 0.104 |
| Endometrial Cancer Signaling | 0.188 | 0.045 | 0.251 | 0.546 |
| ERK/MAPK Signaling | 0.124 | 0.083 | 0.100 | 0.033 |
| Glioblastoma Multiforme Signaling | 0.015 | 0.021 | 0.363 | 0.195 |
| Glioma Signaling | 0.018 | 0.036 | 0.149 | 0.317 |
| HER-2 Signaling in Breast Cancer | 0.004 | 0.013 | 0.017 | 0.107 |
| Molecular Mechanisms of Cancer | 0.033 | 0.014 | 0.072 | 0.087 |
| Non-Small Cell Lung Cancer Signaling | 0.015 | 0.020 | 0.255 | 0.030 |
| Ovarian Cancer Signaling | 0.078 | 0.014 | 0.251 | 0.107 |
| Pancreatic Adenocarcinoma Signaling | 0.021 | 0.183 | 0.163 | 0.337 |
| PI3K/AKT Signaling | 0.035 | 0.224 | 0.085 | 1.000 |
| Prostate Cancer Signaling | 0.021 | 0.224 | 0.366 | 0.485 |
| PTEN Signaling | 0.029 | 0.005 | 0.163 | 0.195 |
| Role of Tissue Factor in Cancer | 0.144 | 0.046 | 0.030 | 0.348 |
| Small Cell Lung Cancer Signaling | 0.033 | 0.204 | 0.465 | 0.195 |
| Sonic Hedgehog Signaling | 0.130 | 0.130 | 0.035 | 0.136 |
| Wnt/β-catenin Signaling | 0.056 | 0.026 | 0.095 | 0.033 |

*P values were adjusted by the Benjamini-Hochberg method. ^54^ For each trait, methylation probes were filtered at p<0.01. There were no canonical pathways enriched for genes with methylated CpG sites associated with FVC. The underlined p-values reached significance threshold (p<0.05).

**Table S9 (continued).** Canonical pathways enriched for genes with differentially methylated CpG sites associated with forced expiratory volume in one second (FEV_1_), forced vital capacity (FVC), FEV_1_/ FVC ratio and chronic obstructive pulmonary disease (COPD) in the discovery (n= 3,781) dataset identified by Ingenuity Pathway Analysis (IPA) (P_BH_< 0.05).

|  | **P_BH_-value^*^** | | | |
| --- | --- | --- | --- | --- |
| **Trait** | **FEV_1_** | **FVC** | **FEV_1_/FVC** | **COPD** |
| **Ingenuity canonical pathways** |  |  |  |  |
| **Group: Signaling pathway** |  |  |  |  |
| **Category: Cardiovascular** |  |  |  |  |
| Adrenomedullin signaling pathway | 0.056 | 0.019 | 0.135 | 0.008 |
| Apelin Endothelial Signaling Pathway | 0.126 | 0.025 | 0.036 | 0.573 |
| Cardiac β-adrenergic Signaling | 0.063 | 0.005 | 0.017 | 0.079 |
| Cardiac Hypertrophy Signaling | 0.118 | 0.032 | 0.113 | 0.714 |
| Cellular Effects of Sildenafil (Viagra) | 0.131 | 0.019 | 0.087 | 0.033 |
| Endothelin-1 Signaling | 0.322 | 0.111 | 0.030 | 0.195 |
| eNOS Signaling | 0.188 | 0.013 | 0.558 | 0.166 |
| Nitric Oxide Signaling in the Cardiovascular System | 0.019 | 0.001 | 0.091 | 0.195 |
| nNOS Signaling in Skeletal Muscle Cells | 0.056 | 0.039 | 0.046 | 0.525 |
| P2Y Purigenic Receptor Signaling Pathway | 0.065 | 0.015 | 0.011 | 0.113 |
| Renin-Angiotensin Signaling | 0.113 | 0.014 | 0.046 | 0.280 |
| Role of NFAT in Cardiac Hypertrophy | 0.004 | 0.001 | 0.001 | 0.041 |
| Thrombin Signaling | 0.123 | 0.045 | 0.003 | 0.302 |
| **Category: Cell Cycle Regulation** |  |  |  |  |
| ATM Signaling | 0.028 | 0.404 | 0.079 | 0.752 |
| CDK5 Signaling | 0.074 | 0.089 | 0.039 | 0.535 |
| Cell Cycle: G1/S Checkpoint Regulation | 0.029 | 1.000 | 0.448 | 0.518 |
| Cyclins and Cell Cycle Regulation | 0.041 | 1.000 | 0.251 | 0.427 |

*P values were adjusted by the Benjamini-Hochberg method. ^54^ For each trait, methylation probes were filtered at p<0.01. There were no canonical pathways enriched for genes with methylated CpG sites associated with FVC. The underlined p-values reached significance threshold (p<0.05).

**Table S9 (continued).** Canonical pathways enriched for genes with differentially methylated CpG sites associated with forced expiratory volume in one second (FEV_1_), forced vital capacity (FVC), FEV_1_/ FVC ratio and chronic obstructive pulmonary disease (COPD) in the discovery (n= 3,781) dataset identified by Ingenuity Pathway Analysis (IPA) (P_BH_< 0.05).

|  | **P_BH_-value^*^** | | | |
| --- | --- | --- | --- | --- |
| **Trait** | **FEV_1_** | **FVC** | **FEV_1_/FVC** | **COPD** |
| **Ingenuity canonical pathways** |  |  |  |  |
| **Group: Signaling pathway** |  |  |  |  |
| **Category: Cellular Growth** |  |  |  |  |
| Corticotropin Releasing Hormone Signaling | 0.009 | 0.000 | 0.000 | 0.033 |
| CREB Signaling in Neurons | 0.005 | 0.000 | 0.001 | 0.033 |
| EGF Signaling | 0.055 | 0.014 | 0.071 | 0.138 |
| Epithelial Adherens Junction Signaling | 0.257 | 0.032 | 0.160 | 0.165 |
| FGF Signaling | 0.049 | 0.020 | 0.034 | 0.331 |
| Gap Junction Signaling | 0.089 | 0.001 | 0.046 | 0.007 |
| Germ Cell-Sertoli Cell Junction Signaling | 0.177 | 0.032 | 0.577 | 0.197 |
| HGF Signaling | 0.037 | 0.039 | 0.087 | 0.331 |
| Human Embryonic Stem Cell Pluripotency | 0.079 | 0.020 | 0.342 | 0.263 |
| IGF-1 Signaling | 0.025 | 0.006 | 0.085 | 0.055 |
| IL-7 Signaling Pathway | 0.028 | 0.169 | 0.159 | 0.069 |
| ILK Signaling | 0.095 | 0.104 | 0.189 | 0.030 |
| Melanocyte Development and Pigmentation Signaling | 0.145 | 0.026 | 0.133 | 0.033 |
| p70S6K Signaling | 0.058 | 0.164 | 0.015 | 0.557 |
| PEDF Signaling | 0.065 | 0.031 | 0.085 | 0.407 |
| Role of NANOG in Mammalian Embryonic Stem Cell Pluripotency | 0.192 | 0.043 | 0.290 | 0.512 |
| Sertoli Cell-Sertoli Cell Junction Signaling | 0.223 | 0.005 | 0.363 | 0.462 |
| VEGF Family Ligand-Receptor Interactions | 0.118 | 0.046 | 0.149 | 0.302 |

*P values were adjusted by the Benjamini-Hochberg method. ^54^ For each trait, methylation probes were filtered at p<0.01. There were no canonical pathways enriched for genes with methylated CpG sites associated with FVC. The underlined p-values reached significance threshold (p<0.05).

**Table S9 (continued).** Canonical pathways enriched for genes with differentially methylated CpG sites associated with forced expiratory volume in one second (FEV_1_), forced vital capacity (FVC), FEV_1_/ FVC ratio and chronic obstructive pulmonary disease (COPD) in the discovery (n= 3,781) dataset identified by Ingenuity Pathway Analysis (IPA) (P_BH_< 0.05).

|  | **P_BH_-value^*^** | | | |
| --- | --- | --- | --- | --- |
| **Trait** | **FEV_1_** | **FVC** | **FEV_1_/FVC** | **COPD** |
| **Ingenuity canonical pathways** |  |  |  |  |
| **Group: Signaling pathway** |  |  |  |  |
| **Category: Cellular Immune Response** |  |  |  |  |
| Antigen Presentation Pathway | 0.004 | 0.076 | 0.374 | 0.685 |
| Caveolar-mediated Endocytosis Signaling | 0.032 | 0.021 | 0.551 | 0.138 |
| CCR5 Signaling in Macrophages | 0.021 | 0.032 | 0.019 | 0.437 |
| CD28 Signaling in T Helper Cells | 0.065 | 0.032 | 0.325 | 0.362 |
| CXCR4 Signaling | 0.124 | 0.081 | 0.007 | 0.363 |
| Dendritic Cell Maturation | 0.028 | 0.131 | 0.376 | 0.561 |
| GP6 Signaling Pathway | 0.051 | 0.015 | 0.100 | 0.008 |
| IL-4 Signaling | 0.017 | 0.204 | 0.133 | 0.562 |
| IL-8 Signaling | 0.081 | 0.025 | 0.030 | 0.254 |
| Leukocyte Extravasation Signaling | 0.025 | 0.007 | 0.183 | 0.408 |
| Neuroinflammation Signaling Pathway | 0.140 | 0.130 | 0.189 | 0.045 |
| NF-κB Activation by Viruses | 0.087 | 0.046 | 0.149 | 0.682 |
| NF-κB Signaling | 0.138 | 0.049 | 0.113 | 0.475 |
| OX40 Signaling Pathway | 0.024 | 0.385 | 1.000 | 0.681 |
| PKCθ Signaling in T Lymphocytes | 0.017 | 0.043 | 0.041 | 0.320 |
| T Cell Exhaustion Signaling Pathway | 0.005 | 0.078 | 0.168 | 0.590 |
| Th1 and Th2 Activation Pathway | 0.041 | 0.608 | 0.326 | 0.427 |
| Th1 Pathway | 0.035 | 1.000 | 0.518 | 0.317 |
| Th2 Pathway | 0.031 | 0.385 | 0.157 | 0.409 |
| **Category: Cellular Stress and Injury** |  |  |  |  |
| Apelin Pancreas Signaling Pathway | 0.223 | 0.049 | 0.441 | 0.494 |
| UVB-Induced MAPK Signaling | 0.065 | 0.026 | 0.087 | 0.166 |

*P values were adjusted by the Benjamini-Hochberg method. ^54^ For each trait, methylation probes were filtered at p<0.01. There were no canonical pathways enriched for genes with methylated CpG sites associated with FVC. The underlined p-values reached significance threshold (p<0.05).

**Table S9 (continued).** Canonical pathways enriched for genes with differentially methylated CpG sites associated with forced expiratory volume in one second (FEV_1_), forced vital capacity (FVC), FEV_1_/ FVC ratio and chronic obstructive pulmonary disease (COPD) in the discovery (n= 3,781) dataset identified by Ingenuity Pathway Analysis (IPA) (P_BH_< 0.05).

|  | **P_BH_-value^*^** | | | |
| --- | --- | --- | --- | --- |
| **Trait** | **FEV_1_** | **FVC** | **FEV_1_/FVC** | **COPD** |
| **Ingenuity canonical pathways** |  |  |  |  |
| **Group: Signaling pathway** |  |  |  |  |
| **Category: Cytokine signalling** |  |  |  |  |
| Androgen Signaling | 0.055 | 0.078 | 0.008 | 0.755 |
| Hepatic Fibrosis / Hepatic Stellate Cell Activation | 0.520 | 0.179 | 1.000 | 0.033 |
| IL-1 Signaling | 0.118 | 0.106 | 0.030 | 0.755 |
| **Category:** Disease-Specific Pathways |  |  |  |  |
| Huntington's Disease Signaling | 0.109 | 0.026 | 0.240 | 0.363 |
| Leptin Signaling in Obesity | 0.213 | 0.015 | 0.187 | 0.091 |
| Role of Macrophages, Fibroblasts and Endothelial Cells in Rheumatoid Arthritis | 0.027 | 0.006 | 0.048 | 0.165 |
| Type II Diabetes Mellitus Signaling | 0.005 | 0.004 | 0.002 | 0.254 |
| **Category: Growth Factor Signaling** |  |  |  |  |
| Endocannabinoid Developing Neuron Pathway | 0.029 | 0.019 | 0.017 | 0.337 |
| ErbB Signaling | 0.079 | 0.026 | 0.008 | 0.356 |
| GDNF Family Ligand-Receptor Interactions | 0.268 | 0.031 | 0.325 | 0.113 |
| Neurotrophin/TRK Signaling | 0.087 | 0.011 | 0.159 | 0.222 |
| NGF Signaling | 0.078 | 0.026 | 0.223 | 0.426 |
| **Category: Humoral Immune Response** |  |  |  |  |
| B Cell Receptor Signaling | 0.009 | 0.142 | 0.262 | 0.498 |
| Fc Epsilon RI Signaling | 0.025 | 0.158 | 0.034 | 0.462 |
| FcγRIIB Signaling in B Lymphocytes | 0.017 | 0.040 | 0.101 | 0.363 |

*P values were adjusted by the Benjamini-Hochberg method. ^54^ For each trait, methylation probes were filtered at p<0.01. There were no canonical pathways enriched for genes with methylated CpG sites associated with FVC. The underlined p-values reached significance threshold (p<0.05).

**Table S9 (continued).** Canonical pathways enriched for genes with differentially methylated CpG sites associated with forced expiratory volume in one second (FEV_1_), forced vital capacity (FVC), FEV_1_/ FVC ratio and chronic obstructive pulmonary disease (COPD) in the discovery (n= 3,781) dataset identified by Ingenuity Pathway Analysis (IPA) (P_BH_< 0.05).

|  | **P_BH_-value^*^** | | | |
| --- | --- | --- | --- | --- |
| **Trait** | **FEV_1_** | **FVC** | **FEV_1_/FVC** | **COPD** |
| **Ingenuity canonical pathways** |  |  |  |  |
| **Group: Signaling pathway** |  |  |  |  |
| **Category: Intracellular and Second Messenger Signaling** |  |  |  |  |
| α-Adrenergic Signaling | 0.085 | 0.018 | 0.008 | 0.302 |
| Adipogenesis pathway | 0.109 | 0.093 | 0.161 | 0.033 |
| AMPK Signaling | 0.029 | 0.019 | 0.085 | 0.203 |
| Calcium Signaling | 0.114 | 0.019 | 0.007 | 0.030 |
| cAMP-mediated signaling | 0.185 | 0.016 | 1.000 | 0.107 |
| Dopamine-DARPP32 Feedback in cAMP Signaling | 0.031 | 0.020 | 0.007 | 0.033 |
| ERK5 Signaling | 0.514 | 0.504 | 0.030 | 0.337 |
| G Beta Gamma Signaling | 0.063 | 0.008 | 0.000 | 0.617 |
| G-Protein Coupled Receptor Signaling | 0.123 | 0.003 | 0.315 | 0.085 |
| Gα12/13 Signaling | 0.065 | 0.032 | 0.019 | 0.671 |
| Gαi Signaling | 0.138 | 0.026 | 0.229 | 0.607 |
| GABA Receptor Signaling | 0.060 | 0.018 | 0.002 | 0.008 |
| GPCR-Mediated Integration of Enteroendocrine Signaling Exemplified by an L Cell | 0.354 | 0.071 | 0.240 | 0.035 |
| GPCR-Mediated Nutrient Sensing in Enteroendocrine Cells | 0.014 | 0.006 | 0.008 | 0.033 |
| Gustation Pathway | 0.065 | 0.002 | 0.033 | 0.121 |
| Insulin Receptor Signaling | 0.009 | 0.004 | 0.076 | 0.292 |
| Phospholipase C Signaling | 0.138 | 0.168 | 0.005 | 0.219 |
| Protein Kinase A Signaling | 0.035 | 0.011 | 0.091 | 0.033 |
| Signaling by Rho Family GTPases | 0.223 | 0.078 | 0.112 | 0.037 |
| Tec Kinase Signaling | 0.035 | 0.095 | 0.046 | 0.577 |

*P values were adjusted by the Benjamini-Hochberg method. ^54^ For each trait, methylation probes were filtered at p<0.01. There were no canonical pathways enriched for genes with methylated CpG sites associated with FVC. The underlined p-values reached significance threshold (p<0.05).

**Table S9 (continued).** Canonical pathways enriched for genes with differentially methylated CpG sites associated with forced expiratory volume in one second (FEV_1_), forced vital capacity (FVC), FEV_1_/ FVC ratio and chronic obstructive pulmonary disease (COPD) in the discovery (n= 3,781) dataset identified by Ingenuity Pathway Analysis (IPA) (P_BH_< 0.05).

|  | **P_BH_-value^*^** | | | |
| --- | --- | --- | --- | --- |
| **Trait** | **FEV_1_** | **FVC** | **FEV_1_/FVC** | **COPD** |
| **Ingenuity canonical pathways** |  |  |  |  |
| **Group: Signaling pathway** |  |  |  |  |
| **Category: Neurotransmitters and Other Nervous System Signaling** |  |  |  |  |
| Agrin Interactions at Neuromuscular Junction | 0.483 | 0.266 | 0.047 | 0.165 |
| Melatonin Signaling | 0.076 | 0.036 | 0.104 | 0.617 |
| Netrin Signaling | 0.006 | 0.001 | 0.002 | 0.046 |
| Neuropathic Pain Signaling In Dorsal Horn Neurons | 0.021 | 0.002 | 0.046 | 0.000 |
| Opioid Signaling Pathway | 0.000 | 0.001 | 0.005 | 0.030 |
| Reelin Signaling in Neurons | 0.028 | 0.019 | 0.149 | 0.385 |
| Synaptic Long Term Depression | 0.022 | 0.026 | 0.002 | 0.048 |
| Synaptic Long Term Potentiation | 0.078 | 0.013 | 0.124 | 0.107 |
| Amyotrophic Lateral Sclerosis Signaling | 0.391 | 0.107 | 0.192 | 0.035 |
| Axonal Guidance Signaling | 0.056 | 0.000 | 0.002 | 0.000 |
| Cholecystokinin/Gastrin-mediated Signaling | 0.166 | 0.479 | 0.032 | 1.000 |
| Circadian Rhythm Signaling | 1.000 | 0.311 | 0.316 | 0.050 |
| Ephrin B Signaling | 0.223 | 0.537 | 0.005 | 0.701 |
| Ephrin Receptor Signaling | 0.596 | 0.298 | 0.046 | 0.337 |
| Glutamate Receptor Signaling | 0.553 | 0.078 | 0.131 | 0.013 |
| GNRH Signaling | 0.017 | 0.002 | 0.002 | 0.288 |

*P values were adjusted by the Benjamini-Hochberg method. ^54^ For each trait, methylation probes were filtered at p<0.01. There were no canonical pathways enriched for genes with methylated CpG sites associated with FVC. The underlined p-values reached significance threshold (p<0.05).

**Table S9 (continued).** Canonical pathways enriched for genes with differentially methylated CpG sites associated with forced expiratory volume in one second (FEV_1_), forced vital capacity (FVC), FEV_1_/ FVC ratio and chronic obstructive pulmonary disease (COPD) in the discovery (n= 3,781) dataset identified by Ingenuity Pathway Analysis (IPA) (P_BH_< 0.05).

|  | **P_BH_-value^*^** | | | |
| --- | --- | --- | --- | --- |
| **Trait** | **FEV_1_** | **FVC** | **FEV_1_/FVC** | **COPD** |
| **Ingenuity canonical pathways** |  |  |  |  |
| **Group: Signaling pathway** |  |  |  |  |
| **Category: Nuclear Receptor Signaling** |  |  |  |  |
| PXR/RXR Activation | 0.127 | 0.043 | 1.000 | 1.000 |
| RAR Activation | 0.095 | 0.020 | 0.010 | 0.185 |
| TR/RXR Activation | 0.065 | 0.013 | 0.577 | 0.219 |
| **Category: Organismal Growth and Development** |  |  |  |  |
| Actin Cytoskeleton Signaling | 0.192 | 0.350 | 0.149 | 0.047 |
| Inhibition of Matrix Metalloproteases | 0.705 | 0.037 | 0.706 | 0.685 |
| Paxillin Signaling | 0.031 | 0.014 | 0.135 | 0.113 |
| Regulation of the Epithelial-Mesenchymal Transition Pathway | 0.017 | 0.005 | 0.131 | 0.033 |
| Relaxin Signaling | 0.236 | 0.003 | 0.087 | 0.055 |
| Sperm Motility | 0.037 | 0.018 | 0.157 | 0.033 |
| **Category: Organismal Growth and Development** |  |  |  |  |
| Mechanisms of Viral Exit from Host Cells | 0.098 | 1.000 | 0.050 | 0.170 |

*P values were adjusted by the Benjamini-Hochberg method. ^54^ For each trait, methylation probes were filtered at p<0.01. There were no canonical pathways enriched for genes with methylated CpG sites associated with FVC. The underlined p-values reached significance threshold (p<0.05).

**Table S10.** Significant KEGG pathways (FDR < 0.05) associated forced expiratory volume in one second (FEV_1_), forced vital capacity (FVC), FEV_1_/FVC ratio and chronic obstructive pulmonary disease (COPD) co-methylation-expression network (p < 0.05) identified using the *Significance-based Modules Integrating the Transcriptome and Epigenome (*SMITE) R package^56^ including genes with differentially methylated sites associated with FEV_1_ in the discovery Generation Scotland: Scottish Family Health Survey (GS:SFHS) cohort data.

| **FEV_1_** | | | | | |
| --- | --- | --- | --- | --- | --- |
| **Module 1: Built around NTN1 and DCC genes.** | | | | | |
| **Genes (N=27):** NTN1, ABLIM1, DSCAM, ABLIM3, UNC5C, DCC, ABLIM2, UNC5A, UNC5B, NEO1, UNC5D, **SIAH2**, PITPNA, SIAH1, SLIT3, SLIT1, APPL1, AGAP2, RGMA, RGMB, ROBO1, NTN3, HFE2, DAPK1, NTN4, ROBO3, ROBO2 | | | | | |
| **Significant KEGG pathways:** | | | | | |
| **Category** | **Category name** | **Class** | **P-value** | **Genes** | |
|  |  |  |  | **Significant** | **Total** |
| hsa04360 | Axon guidance | Organismal Systems | 0.00000 | 16 | 128 |
| hsa05210 | Colorectal cancer | Human Diseases | 0.03654 | 2 | 62 |
| **Module 2: Built around STAT3 gene.** | | | | | |
| **Genes (N=35):** STAT3, TRIM24, JAK2, GH1, GH2, IL6, IL6R, PDGFRB, KIT, GHR, KITLG, CUX1, IL6ST, LEP, STAT1, STAT5A, STAT5B, POU5F1, FGFR1OP2, ZMYM2, LEPR, **SOCS3**, CISH, SH2B3, FER, PRL, PRLR, SH2B1, SOCS2, PTPRU, PIAS1, GRB10, PTPN2, GRAP, SOCS6 | | | | | |
| **Significant KEGG pathways:** | | | | | |
| **Category** | **Category name** | **Class** | **P-value** | **Genes** | |
|  |  |  |  | **Significant** | **Total** |
| hsa04060 | Cytokine-cytokine receptor interaction | Environmental Information Processing | 0.00000 | 13 | 263 |
| hsa04630 | Jak-STAT signaling pathway | Environmental Information Processing | 0.00000 | 19 | 155 |
| hsa04920 | Adipocytokine signaling pathway | Organismal Systems | 0.00002 | 5 | 68 |
| hsa05200 | Pathways in cancer | Human Diseases | 0.00003 | 9 | 322 |
| hsa04080 | Neuroactive ligand-receptor interaction | Environmental Information Processing | 0.00010 | 7 | 271 |
| hsa04640 | Hematopoietic cell lineage | Organismal Systems | 0.00026 | 4 | 88 |
| hsa05221 | Acute myeloid leukemia | Human Diseases | 0.00027 | 4 | 57 |
| hsa05160 | Hepatitis C | Human Diseases | 0.00268 | 4 | 133 |
| hsa04062 | Chemokine signaling pathway | Organismal Systems | 0.01123 | 4 | 186 |
| hsa05145 | Toxoplasmosis | Human Diseases | 0.01892 | 3 | 132 |
| hsa05140 | Leishmaniasis | Human Diseases | 0.02851 | 2 | 72 |
| hsa04930 | Type II diabetes mellitus | Human Diseases | 0.03515 | 2 | 48 |
| **FVC** | | | | | |
| **Module 1: Built around NTN1 gene.** | | | | | |
| **Genes (N=23):** NTN1, ABLIM1, DSCAM, ABLIM3, UNC5C, DCC, ABLIM2, UNC5A, UNC5B, NEO1, UNC5D, **SIAH2**, PITPNA, SIAH1, AGAP2, RGMB, RGMA, SLIT3, DAPK1, HFE2, NTN3, SLIT1, APPL1 | | | | | |
| **Significant KEGG pathways:** | | | | | |
| **Category** | **Category name** | **Class** | **P-value** | **Genes** | |
|  |  |  |  | **Significant** | **Total** |
| hsa04360 | Axon guidance | Organismal Systems | 0.00000 | 12 | 128 |
| hsa05210 | Colorectal cancer | Human Diseases | 0.02373 | 2 | 62 |
| **FEV_1_/FVC** | | | | | |
| **Module 1: Built around UNC5A gene.** | | | | | |
| **Genes (N=20):** UNC5A, UNC5C, NTN1, DCC, UNC5B, MAGED1, NTN4, UNC5D, SIAH1, PITPNA, **SIAH2**, SLIT3, SLIT1, DSCAM, APPL1, DAPK1, ABLIM3, ABLIM2, ABLIM1,AGAP2 | | | | | |
| **Category** | **Category name** | **Class** | **P-value** | **Genes** | |
|  |  |  |  | **Significant** | **Total** |
| hsa04360 | Axon guidance | Organismal Systems | 0.00000 | 12 | 128 |
| hsa05210 | Colorectal cancer | Human Diseases | 0.02236 | 2 | 62 |
| **COPD** | | | | | |
| **Module1: Built around DCC gene.** | | | | | |
| **Genes (N=20):** DCC, ABLIM1, DSCAM, ABLIM3, UNC5C, NTN1, ABLIM2, UNC5A, UNC5B, NTN4, UNC5D, **SIAH2**, PITPNA, SIAH1, APPL1, SLIT1, SLIT3, AGAP2, DAPK1, MAGED1 | | | | | |
| **Significant KEGG pathways:** | | | | | |
| **Category** | **Category name** | **Class** | **P-value** | **Genes** | |
|  |  |  |  | **Significant** | **Total** |
| hsa04360 | Axon guidance | Organismal Systems | 0.00000 | 12 | 128 |
| hsa05210 | Colorectal cancer | Human Diseases | 0.02236 | 2 | 62 |

Genome-wide significant differentially methylated sites associated with forced expiratory volume in one second (FEV_1_) in the discovery GS:SFHS cohort data are highlighted in bold.

**Table S11.** Characteristics of participants in the test and training data used to assess the predictive value of the differentially methylated sites (DMSs) in the prognosis of chronic obstructive pulmonary disease (COPD) in the independent prediction data (N=149) from the discovery Generation Scotland: Scottish Family Health Survey cohort.

|  | **Training data** | | **Test data** | |
| --- | --- | --- | --- | --- |
|  | **Cases**  **(n = 47)** | ***Controls**  **(n = 48)** | **Cases**  **(n = 24)** | ***Controls**  **(n = 30)** |
| **Characteristics** |  |  |  |  |
| **Age, years** | 59.93 ± 9.32 | 53.89 ± 8.14 | 64.34± 9.74 | 53.09 ± 10.23 |
| **Sex:** |  |  |  |  |
| – Male | 15 (31.9) | 17 (35.4) | 10 (41.7) | 11 (36.7) |
| – Female | 32 (68.1) | 31 (64.6) | 14 (58.3) | 19 (63.3) |
| **Height, cm** | 162.37 ± 9.58 | 167.24 ± 7.72 | 165.69 ± 10.05 | 166.54 ± 6.74 |
| **Smoking status:** |  |  |  |  |
| – Never | 4 (8.5) | 28 (58.3) | 2 (8.3) | 15 (50.0) |
| – Former (quit ≥ 12 months) | 14 (29.8) | 13 (27.1) | 10 (41.7) | 11 (36.7) |
| – Current | 26 (55.3) | 6 (12.5) | 9 (37.5) | 3 (10.0) |
| – Missing records | 3 (6.4) | 1 (2.1) | 3 (12.5) | 1 (3.3) |
| **Pack-year:** |  |  |  |  |
| – Former (quit ≥ 12 months) | 39.36 ± 31.09 | 13.54 ± 13.73 | 47.10 ± 63.25 | 10.09 ± 10.99 |
| – Current | 31.50 ± 22.36 | 19.83 ± 17.05 | 39.00 ± 37.54 | 10.33 ± 11.06 |

Figures shown are the mean ± standard deviation or n (%). *Controls were defined based on spirometry data. Former smokers (quit < 12 months) were combined with current smokers due to insufficient number of records in the prediction data.

**Table S12.** Characteristics of participants in the replication data (N=178) used to assess the predictive value of the differentially methylated sites (DMSs) identified in the discovery Generation Scotland: Scottish Family Health Survey cohort in the diagnosis of chronic obstructive pulmonary disease (COPD) in the Lothian Birth Cohort of 1936.

|  | **Replication data** | |
| --- | --- | --- |
|  | **Cases**  **(n = 89)** | **Controls**  **(n = 89)** |
| **Characteristics** |  |  |
| **Age, years** | 69.60 ± 0.80 | 69.65 ± 0.2 |
| **Sex:** |  |  |
| – Male | 44 (49.4) | 41 (46.1) |
| – Female | 45 (50.6) | 48 (53.9) |
| **Height, cm** | 165.7 ± 9.70 | 165.2 ± 9.38 |
| **Smoking status:** |  |  |
| – Never | 20 (22.5) | 52 (58.4) |
| – Former (quit ≥ 12 months) | 36 (40.4) | 29 (32.6) |
| – Current | 33 (37.1) | 8 (9.0) |
| **Pack-year:** |  |  |
| – Former (quit ≥ 12 months) | 45.91 ± 43.25 | 22.79 ± 30.76 |
| – Current | 45.64 ± 20.55 | 42.71 ± 14.97 |

Figures shown are the mean ± standard deviation or n (%). *Controls were defined based on spirometry data. Cases and controls were defined based on spirometry data. Former smokers (< 12 months) were combined with current smokers due to insufficient number of records in the replication data.

# Supplementary Figures

###
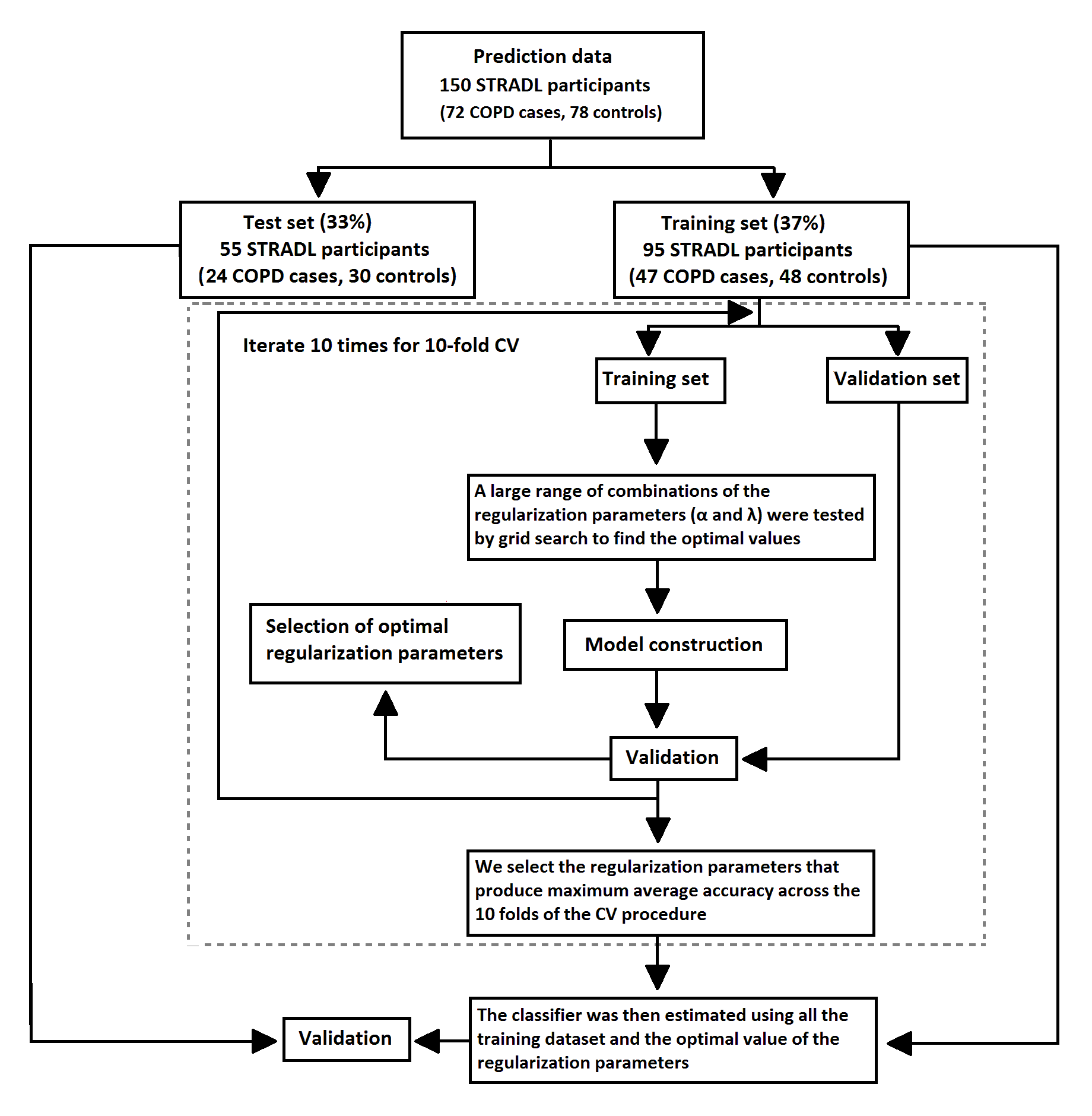


**Figure S1.** Outline of the risk prediction model construction and validation in the discovery Generation Scotland: Scottish Family Health Study cohort.

**
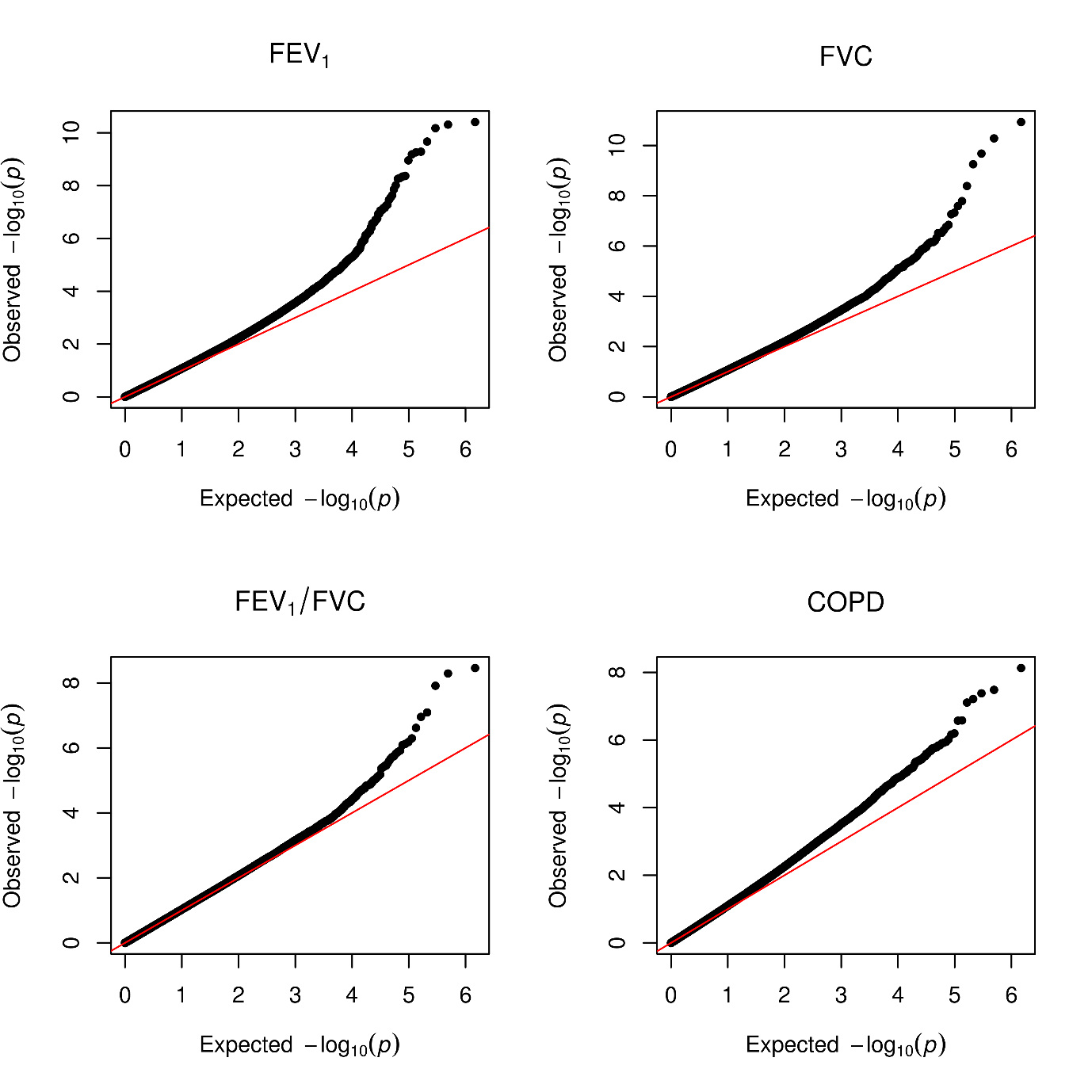
 Figure S2.** Quantile-quantile (Q-Q) plots in –log_10_ scale for the three respiratory function traits (FEV_1_, FVC, and FEV_1_/FVC) and COPD. The red line represents the concordance of observed and expected values in the discovery Generation Scotland: Scottish Family Health Survey (GS:SFHS) cohort data. The inflation factors (λ) for FEV_1_, FVC, FEV_1_/FVC, and COPD were 1.09, 1.08, 1.04 and 1.12 respectively. λ was calculated as a ratio of the median of the observed chi-square statistics for association over the expected median (0.4549) of the chi-square distribution with 1 df.


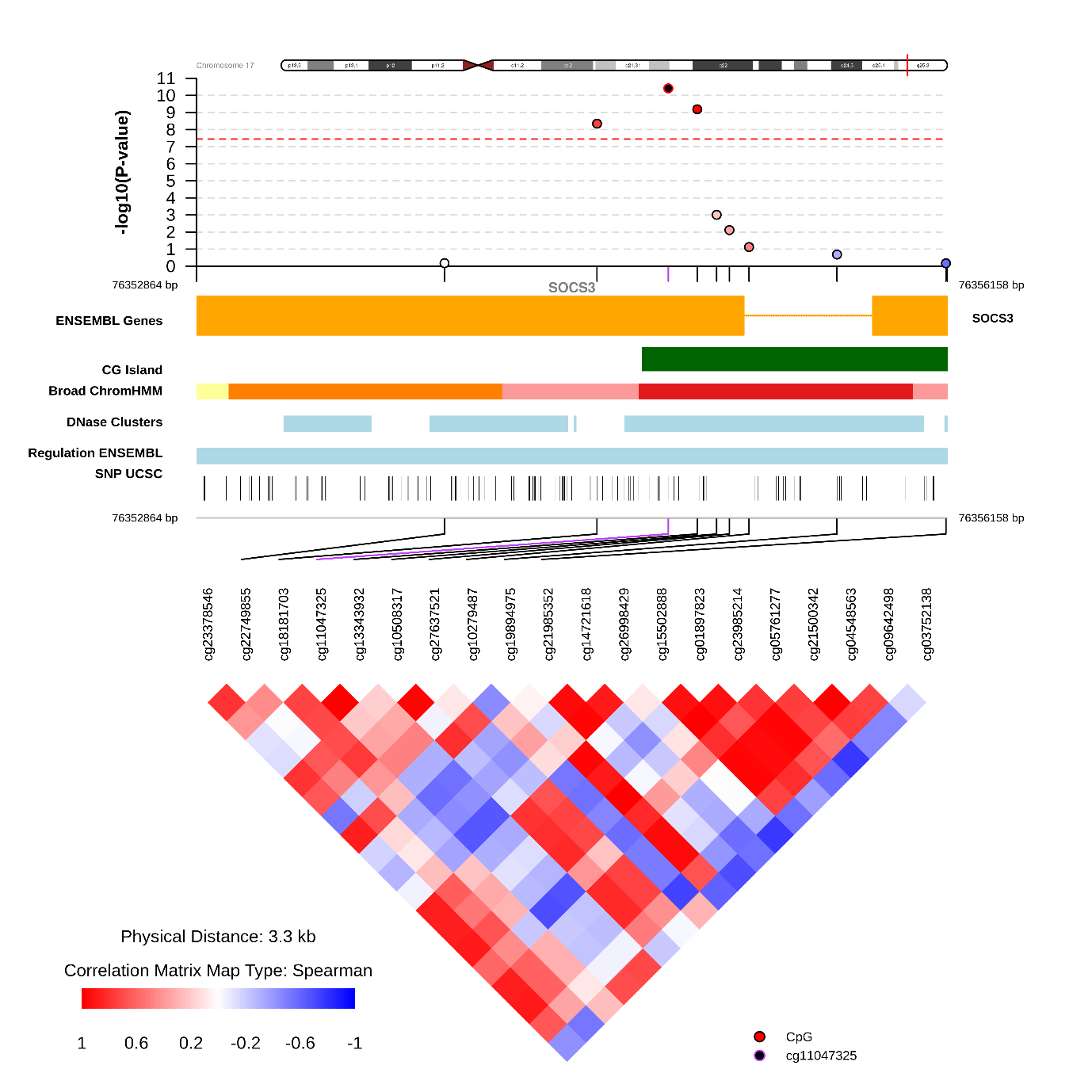


**Figure S3.** Regional association plot and genomic annotation surrounding the index cg11047325 CpG site in the chromosomal region 17q25.3 (chr17:76,352,864-76,356,158, hg19) encompassing the SOCS3 (suppressor of cytokine signaling 3 (SOCS3) gene. *Y*-axis: -log_10_(*P*-values) for the association between methylation and FEV_1_; *x*-axis: chromosomal position. Each dot represents an evaluated CpG; the color-coding reflects the degree of correlation of methylation values at all other CpGs in the figure with that of the index site (red: positive correlation, blue: negative correlation) in the discovery Generation Scotland: Scottish Family Health Survey (GS:SFHS) cohort data (N=3,781). The index probe cg11047325 is represented as a black dot.We display genes symbols (Ensembl), CpG islands (UCSC), Broad ChromHMM domains (UCSC), DNaseI clusters (UCSC), Ensembl regulation tracks, and *cis*-meQTL-SNPs (UCSC).


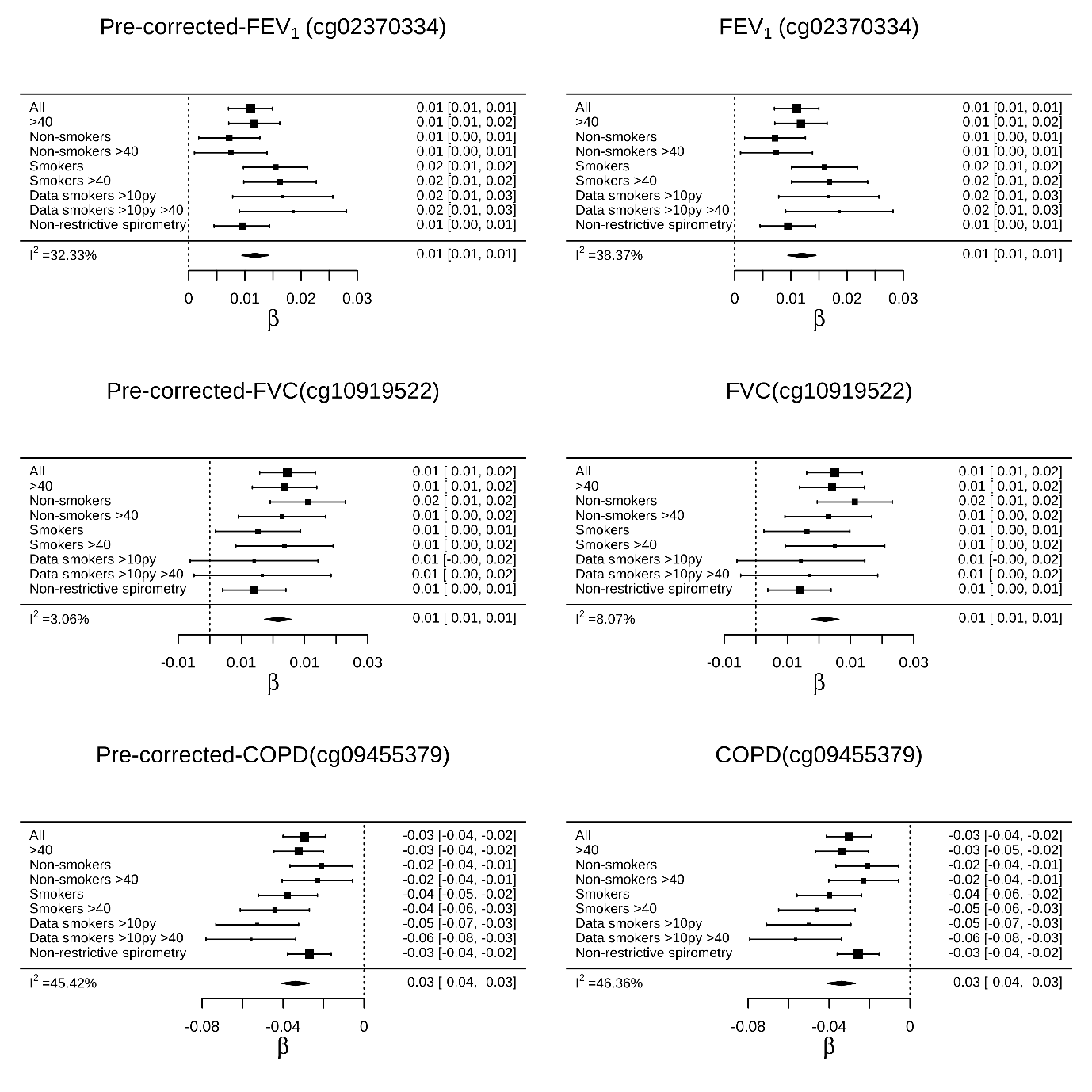
**Figure S5.** Forest plot and meta-analysis comparing the differentially methylated sites associated with FEV_1_, FVC and COPD that were pre-corrected or non-pre-corrected for smoking history that showed marginally higher heterogeneity in older adult (> 40 years), smokers with a substantial smoking history, individuals with non-restrictive spirometry pattern, and stratified smoking status datasets from the discovery Generation Scotland: Scottish Family Health Survey (GS:SFHS) cohort when trait data was not pre-corrected for smoking history. The sizes of the forest plot squares are proportional to the amount of information each dataset contains.

**
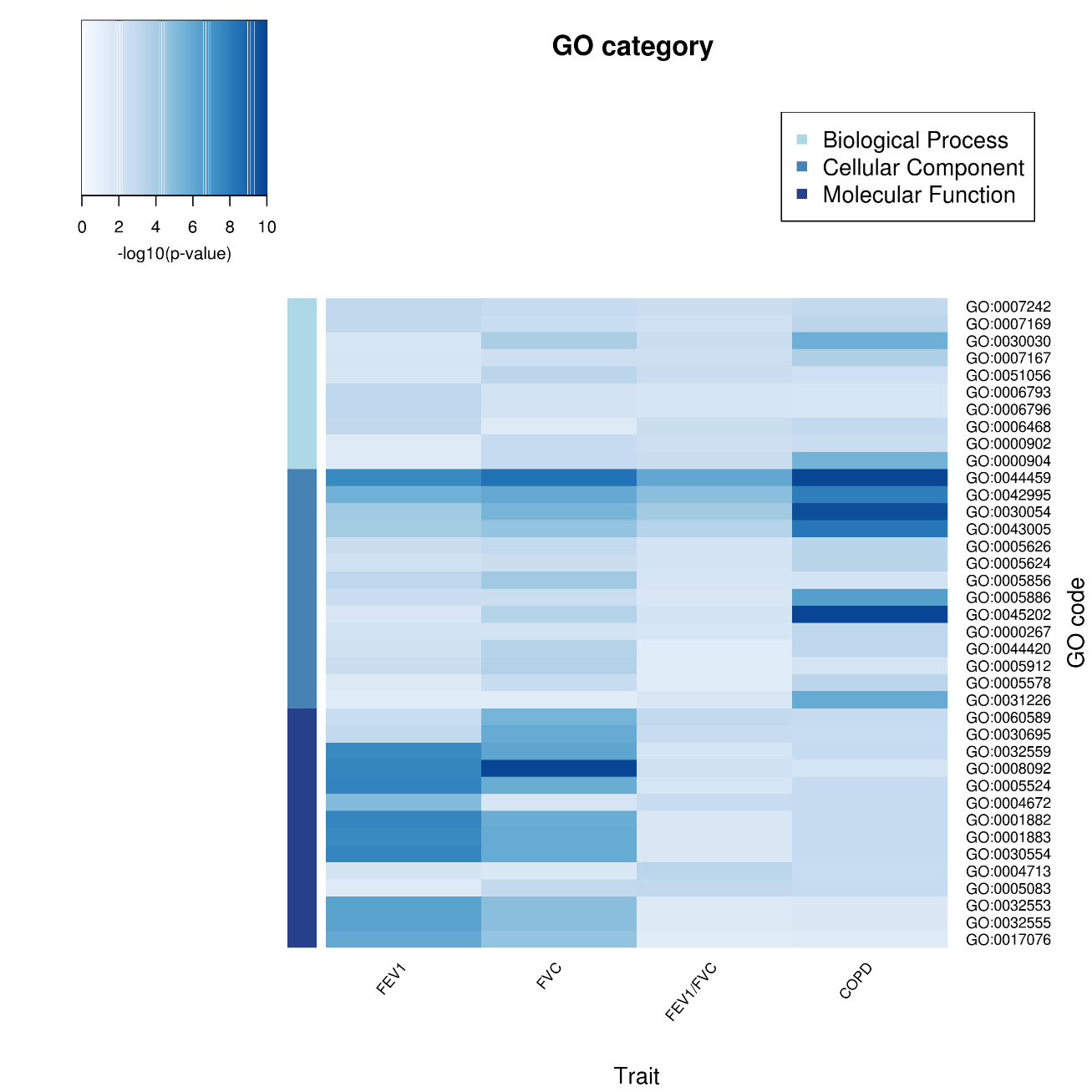
Figure S6.** Gene Ontology (GO) terms enriched for genes with differentially methylated methylated CpG sites associated with forced expiratory volume in one second (FEV_1_), forced vital capacity (FVC) and chronic obstructive pulmonary disease (COPD) in the discovery n= 3,781) dataset by Database for Annotation, Visualization and Integrated Discovery (DAVID). The threshold for enrichment significance was set as Benjamini-Hochberg^54^ adjusted p-value <0.05. P values were adjusted by the Benjamini-Hochberg method.^54^ For each trait, methylation probes were filtered at p<0.01. There were no GO terms enriched for genes with methylated CpG sites associated with FEV_1_/FVC ratio. **Biological Process:** GO:0007242:intracellular signaling cascade; GO:0007169:transmembrane receptor protein tyrosine kinase signaling pathway; GO:0030030:cell projection organization; GO:0007167:enzyme linked receptor protein signaling pathway; GO:0051056:regulation of small GTPase mediated signal transduction; GO:0006793:phosphorus metabolic process; GO:0006796:phosphate metabolic process; GO:0006468:protein amino acid phosphorylation; GO:0000902:cell morphogenesis; GO:0000904:cell morphogenesis involved in differentiation. **Cellular Component:** GO:0044459:plasma membrane part; GO:0042995:cell projection; GO:0030054:cell junction; GO:0043005:neuron projection; GO:0005626:insoluble fraction; GO:0005624:membrane fraction; GO:0005856:cytoskeleton; GO:0005886:plasma membrane; GO:0045202:synapse; GO:0000267:cell fraction; GO:0044420:extracellular matrix part; GO:0005912:adherens junction; GO:0005578:proteinaceous extracellular matrix; GO:0031226:intrinsic to plasma membrane. **Molecular Function:** GO:0060589:nucleoside-triphosphatase regulator activity; GO:0030695:GTPase regulator activity; GO:0032559:adenyl ribonucleotide binding; GO:0008092:cytoskeletal protein binding; GO:0005524:ATP binding; GO:0004672:protein kinase activity; GO:0001882:nucleoside binding; GO:0001883:purine nucleoside binding; GO:0030554:adenyl nucleotide binding; GO:0004713:protein tyrosine kinase activity; GO:0005083:small GTPase regulator activity; GO:0032553:ribonucleotide binding; GO:0032555:purine ribonucleotide binding; GO:0017076:purine nucleotide binding.

**Cellular Component**

GO:0005737:cytoplasm; GO:0005829:cytosol; GO:0030054:cell junction; GO:0016020:membrane; GO:0005856:cytoskeleton; GO:0015629:actin cytoskeleton; GO:0048471:perinuclear region of cytoplasm; GO:0030018:Z disc; GO:0070062:extracellular exosome; GO:0016459:myosin complex; GO:0014069:postsynaptic density; GO:0005913:cell-cell adherens junction; GO:0017053:transcriptional repressor complex; GO:0030425:dendrite; GO:0005938:cell cortex; GO:0030673:axolemma; GO:0031982:vesicle; GO:0005925:focal adhesion; GO:0043197:dendritic spine; GO:0005794:Golgi apparatus; GO:0045211:postsynaptic membrane; GO:0045202:synapse; GO:0000790:nuclear chromatin.

**
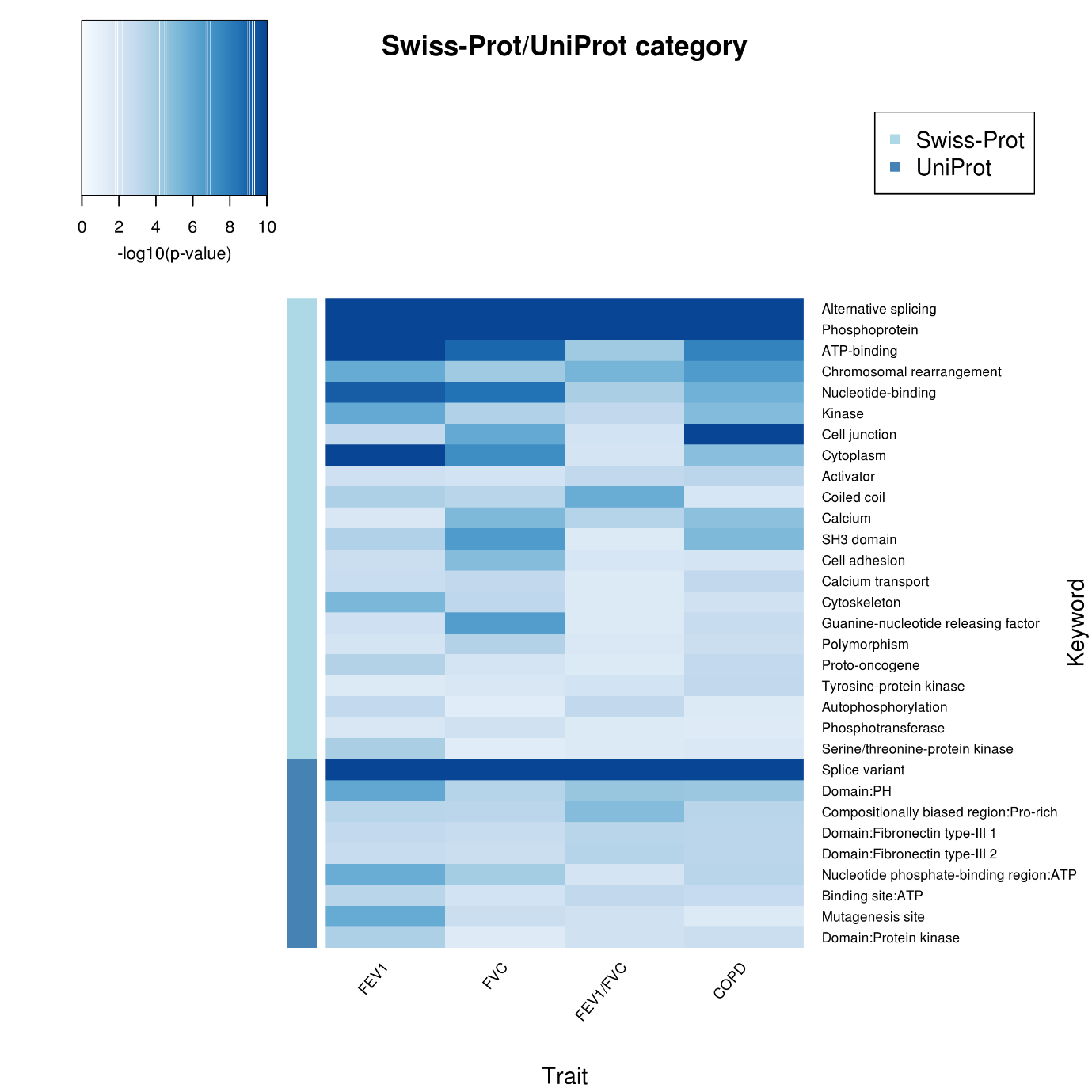
Figure S7.** Swiss-Prot/UniProt keywords enriched for genes with differentially methylated CpG sites associated with forced expiratory volume in one second (FEV_1_), forced vital capacity (FVC), FEV_1_/FVC ratio and chronic obstructive pulmonary disease (COPD) in the discovery (n= 3,781) dataset by Database for Annotation, Visualization and Integrated Discovery (DAVID). The threshold for enrichment significance was set as Benjamini-Hochberg^54^ adjusted p-value <0.05. P values were adjusted by the Benjamini-Hochberg method.^54^ For each trait, methylation probes were filtered at p<0.01.

**Cellular Component**

GO:0005737:cytoplasm; GO:0005829:cytosol; GO:0030054:cell junction; GO:0016020:membrane; GO:0005856:cytoskeleton; GO:0015629:actin cytoskeleton; GO:0048471:perinuclear region of cytoplasm; GO:0030018:Z disc; GO:0070062:extracellular exosome; GO:0016459:myosin complex; GO:0014069:postsynaptic density; GO:0005913:cell-cell adherens junction; GO:0017053:transcriptional repressor complex; GO:0030425:dendrite; GO:0005938:cell cortex; GO:0030673:axolemma; GO:0031982:vesicle; GO:0005925:focal adhesion; GO:0043197:dendritic spine; GO:0005794:Golgi apparatus; GO:0045211:postsynaptic membrane; GO:0045202:synapse; GO:0000790:nuclear chromatin.


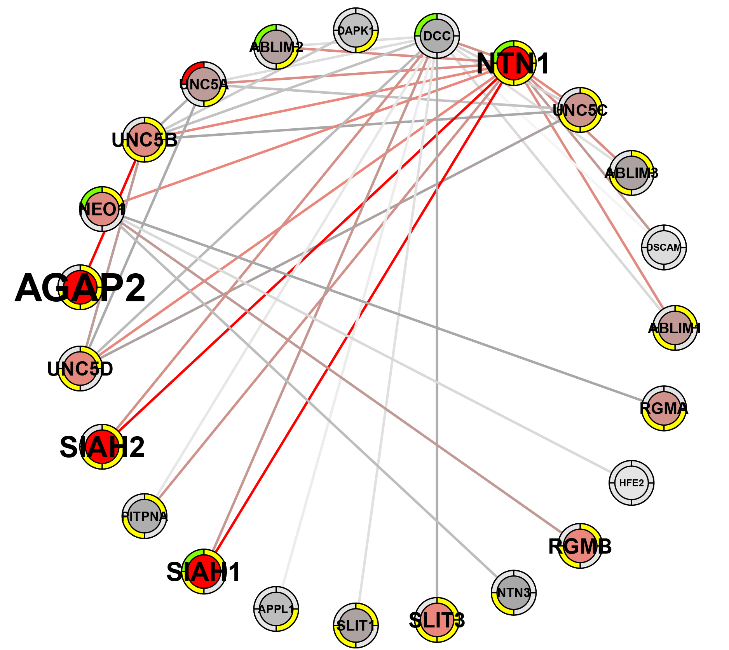


**B**


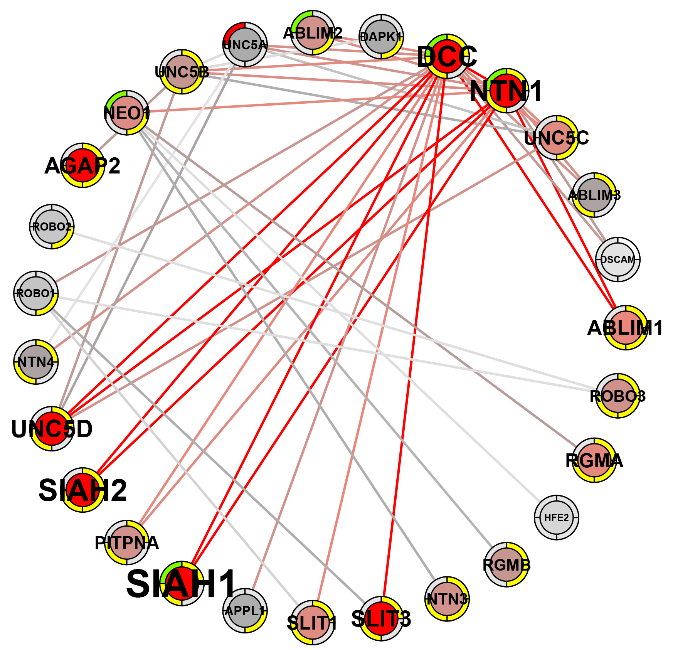


**A**


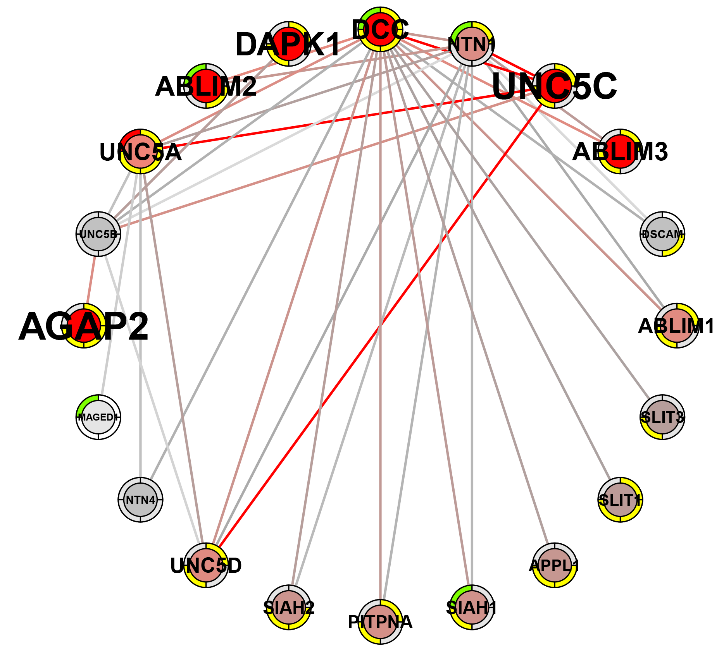


**D**


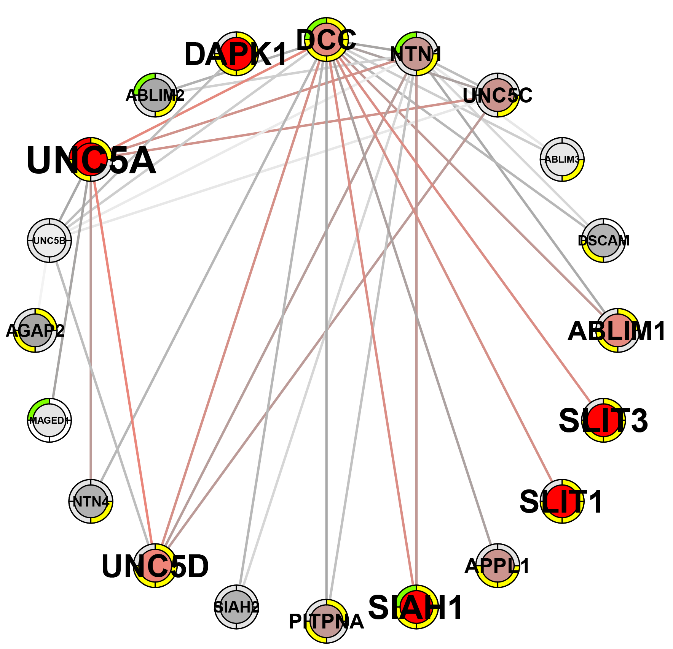


**C**


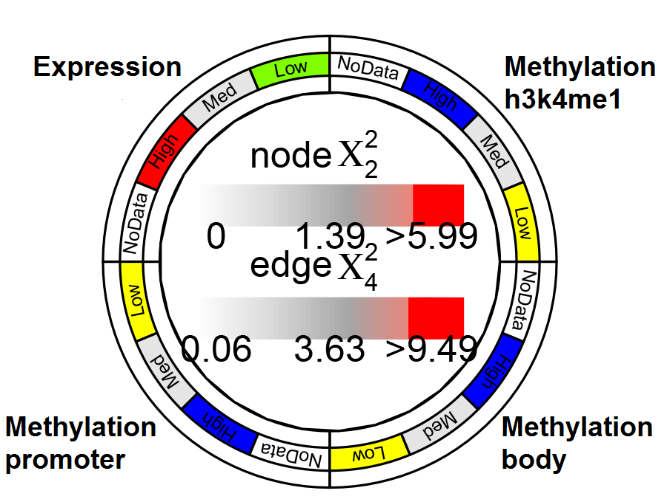


**Figure S8.** Forced expiratory volume in one second (FEV_1_; **A;** N = 27 genes), forced vital capacity (FVC; **B;** N = 23 genes), FEV_1_/FVC ratio (**C;** N = 20 genes) and chronic obstructive pulmonary disease (COPD; **D;** N = 20 genes) co-methylation-expression networks built around NTN1, DCC, and UNC5D genes and involved in axon guidance and colorectal cancer in the discovery Generation Scotland: Scottish Family Health Survey (GS:SFHS) cohort data. Expression is displayed on the top left edge of each gene circle (upregulated: red; downregulated: green; grey: not significant; white: no data), and combined enhancers (histone H3 lysine 4 monomethylation, H3K4me1), body methylation and promoter are displayed on the bottom left and top right of each circle, respectively (hyper-methylated: dark blue; hypomethylated: yellow; grey: not significant; white: no data). The symbol text sizes and centre node colours are based on the total gene score (low (grey) to high (red)), and the edge colours are representative of the strength of the associations between the genes (low (grey) to high (red)). The module shows SIAH2 as a highly (red) to low (grey) scoring gene within the FEV_1_, FVC and COPD networks.


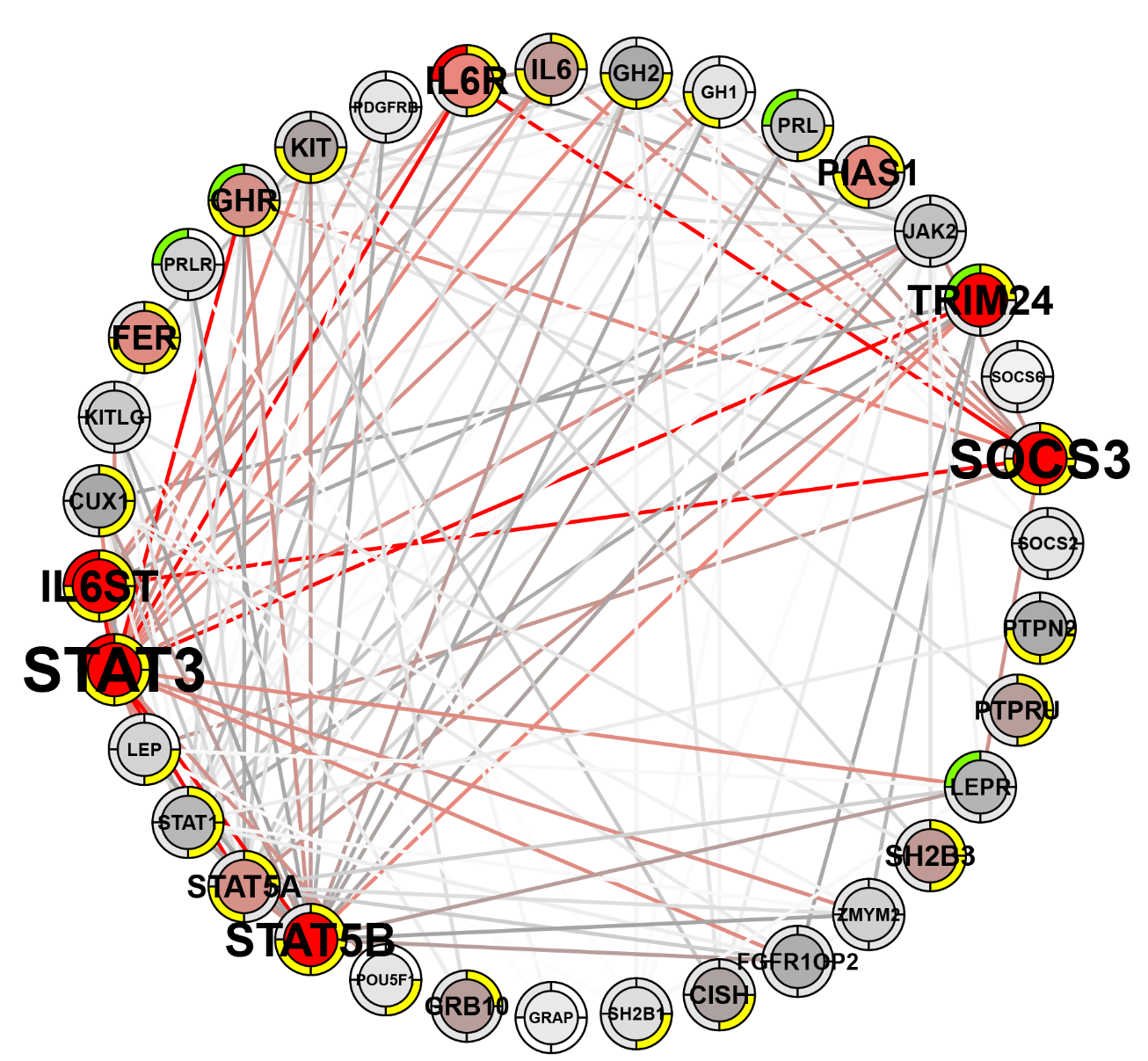


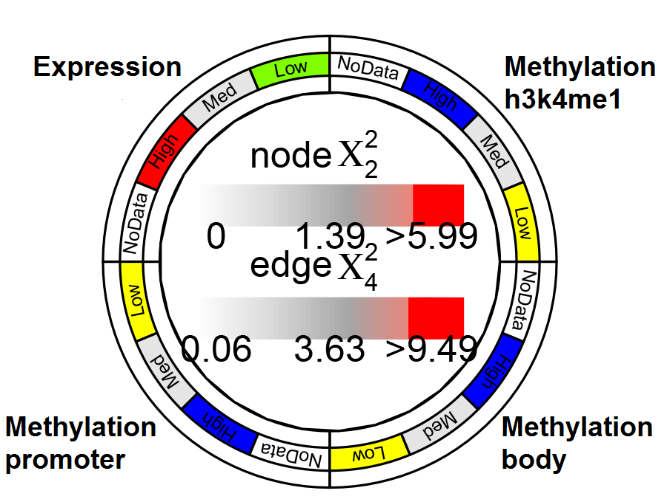


**Figure S9.** Forced expiratory volume in one second (FEV_1;_ N = 35 genes) co-methylation-expression network built around STAT3 gene and involved in cytokine-cytokine receptor interaction and JAK-STAT signalling pathway in the discovery Generation Scotland: Scottish Family Health Survey (GS:SFHS) cohort data. Expression is displayed on the top left edge of each gene circle (upregulated: red; downregulated: green; grey: not significant; white: no data), and combined enhancers (histone H3 lysine 4 monomethylation, H3K4me1), body methylation and promoter are displayed on the bottom left and top right of each circle, respectively (hypermethylated: dark blue; hypo methylated: yellow; grey: not significant; white: no data). The symbol text sizes and centre node colours are based on the total gene score (low (grey) to high (red)), and the edge colours are representative of the strength of the associations between the genes (low (grey) to high (red)). The module shows SOCS3 as a highly scoring gene (red) within the network.


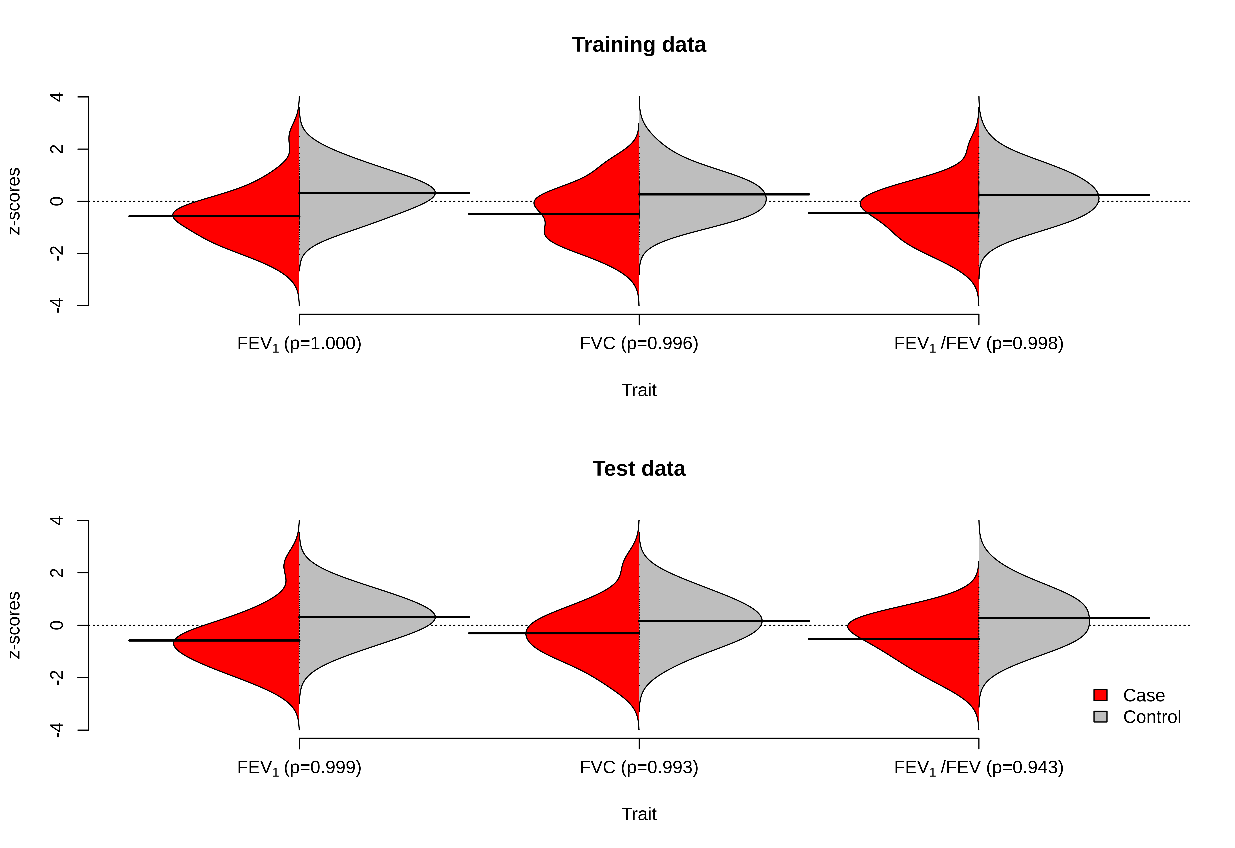


**A**

**B**


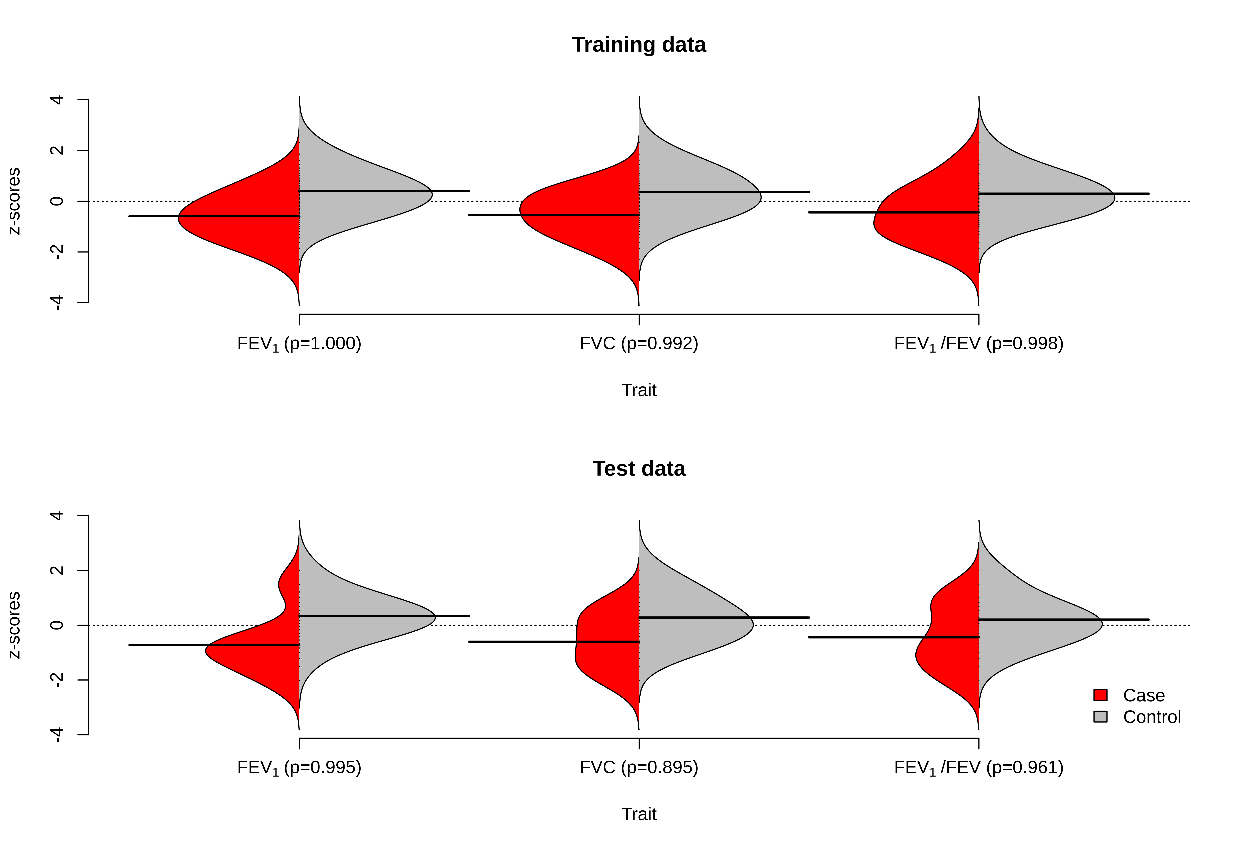


**B**

**Figure S10.** Distribution of z-scores (rank-transformed pre-corrected spirometry traits) in incident cases and controls across the training (N=95) and test (N=54) data sets (**A**), and the training (N=56) and test (N=30) data sets (**B**) in which individuals that had never smoked were removed from the Generation Scotland: Scottish Family Health Survey (GS:SFHS) cohort. FEV_1_, FVC and FEV_1_/FVC were pre-corrected for age, age^2^, sex, height, height^2^, and two smoking variables: smoking status and pack-years of smoking. FVC was pre-corrected for the effect of weight in addition to the above variables. The z-scores from cases are plotted to the left of the centre of each plot with a red distribution; z-scores from controls data are plotted to the right of the centre in each plot and shown with a grey distribution. The mean of each distribution is given as a long black line. The p-values are from the unpaired two-tailed Wilcoxon-test, which showed no significant difference between z-score distributions in incident cases and controls in the training and test data.

**Figure S11.** Receiver operating characteristic curves for the test data. Classification performance of the reduced model and full model differentially methylation sites for discrimination of COPD in the discovery Generation Scotland: Scottish Family Health Survey (GS:SFHS) cohort (**A**), and replication Lothian Birth Cohort of 1936 (**B**) data. Blue line: Receiver operating characteristic (ROC) curve for prediction of COPD, from the reduced model: sex, age, age^2^, height, height, height^2^, smoking status (never, former and current smoker) and pack-years of smoking. Red line: ROC curve for prediction of COPD, from the full model that added differentially methylated sites associated with respiratory function traits and COPD to the reduced model.


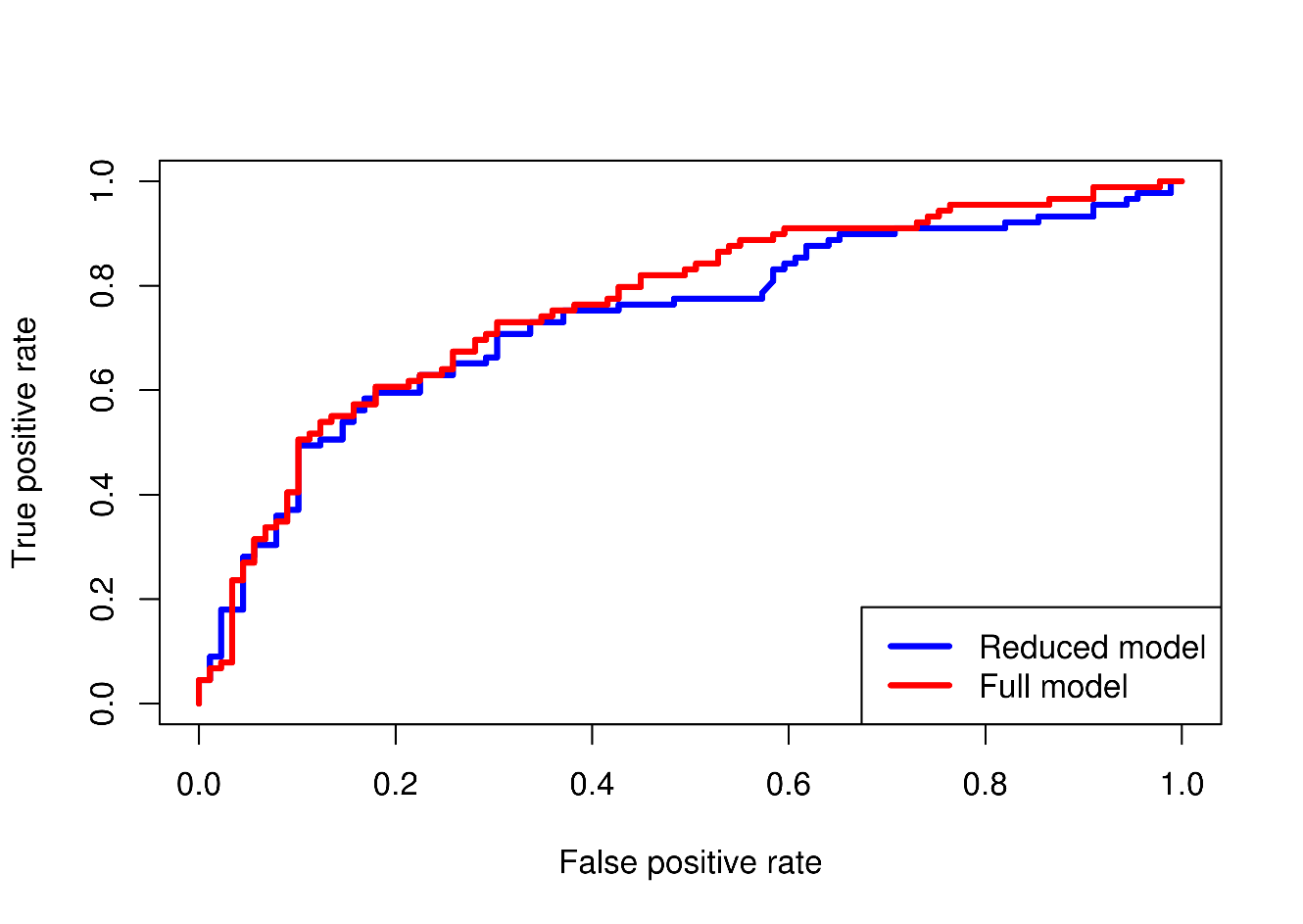

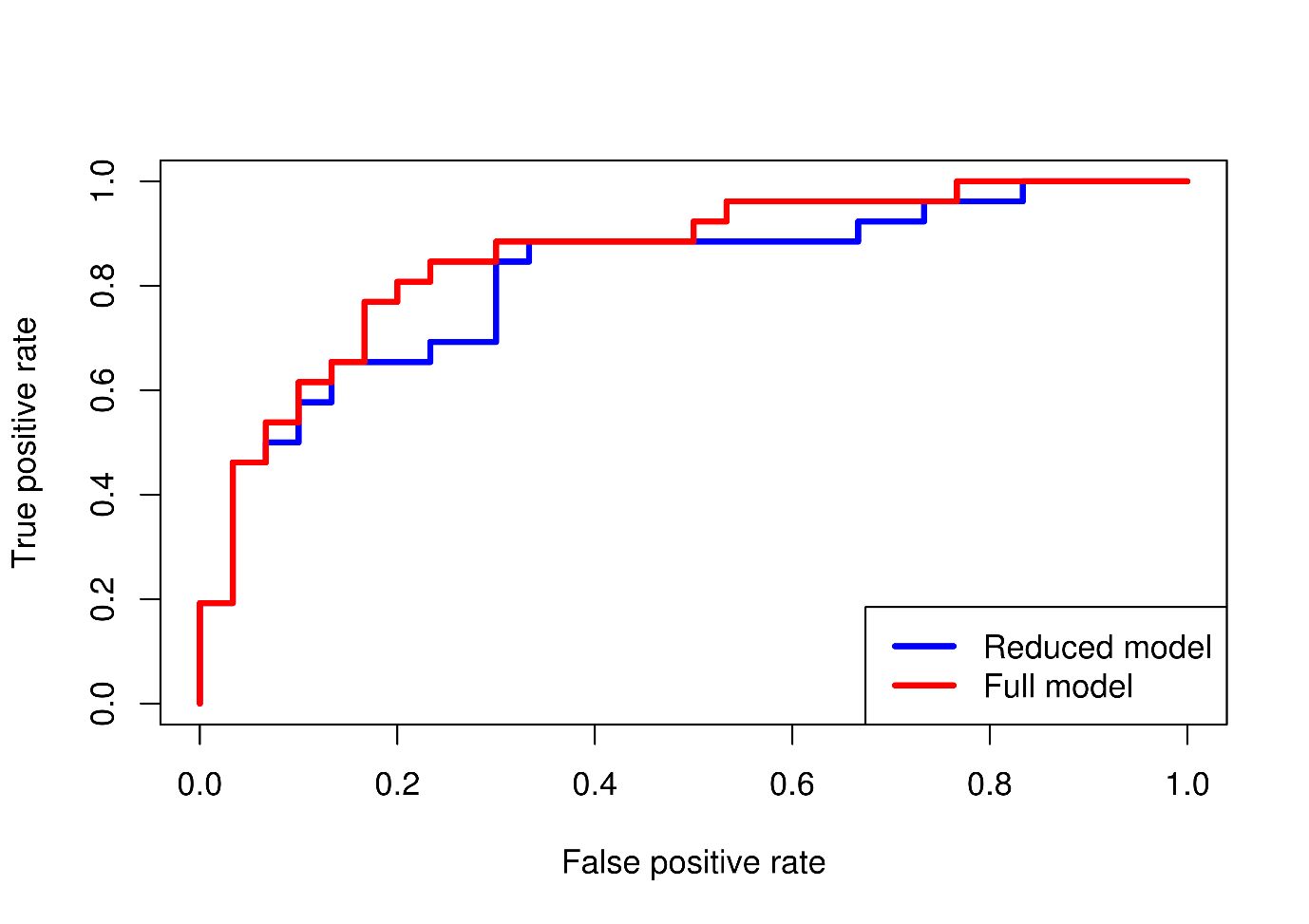


**A**

**B**


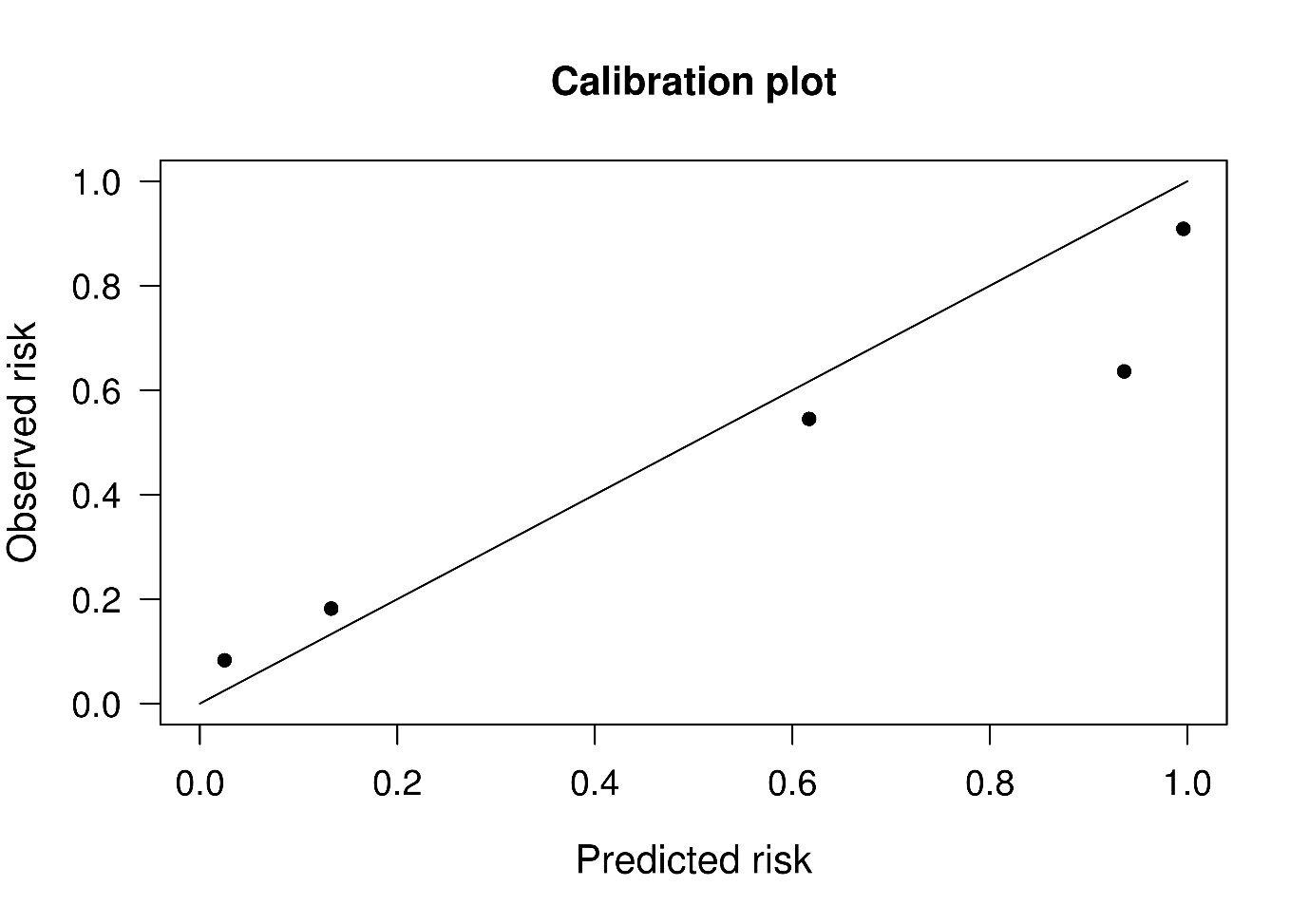


**Figure S12. Calibration plot of the predicted from the full** model (including: sex, age, age^2^, height, height, height^2^, smoking status [never, former and current smoker] and pack-years of smoking, and differentially methylated sites associated with respiratory function traits or COPD) **and observed risk of COPD of 25 incident COPD cases and 30 controls in the test data from the discovery** Generation Scotland: Scottish Family Health Study **(GS:SFHS) cohort.** Hosmer–Lemeshow goodness‐of‐fit p=0.000.

**
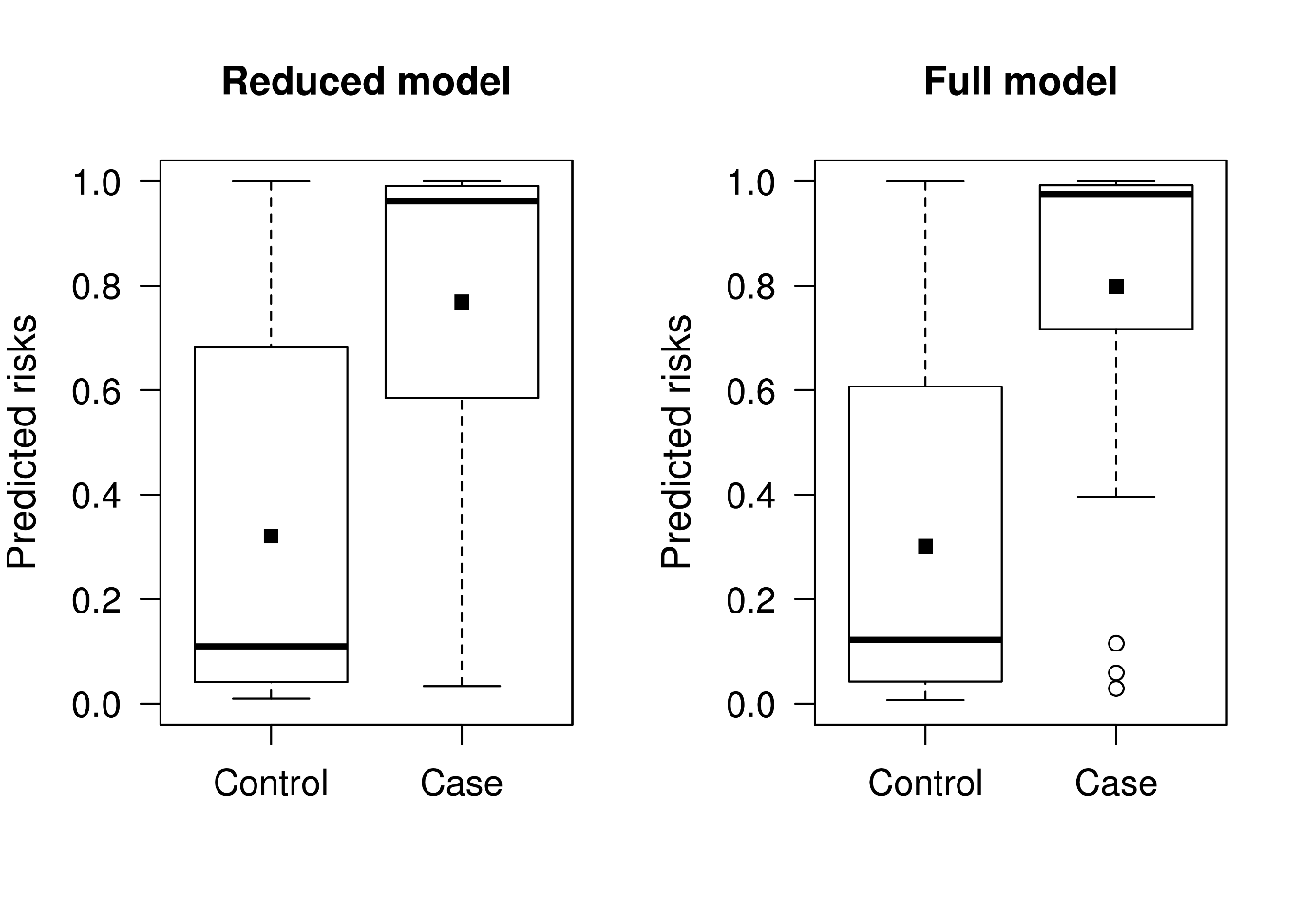
**

**Figure S13**. Box plots of predicted probabilities for COPD cases and controls from the reduced (including: sex, age, age^2^, height, height, height^2^, smoking status [never, former and current smoker] and pack-years of smoking) and full (which added differentially methylated sites associated with respiratory function traits or COPD to the reduced model) models built in the discovery Generation Scotland: Scottish Family Health Survey (GS:SFHS) cohort data. The discrimination slope is calculated, as the difference between the mean predicted probability for COPD cases and controls (solid squares indicate means). The difference between discrimination slopes is equivalent to the integrated discrimination index (IDI), which is equal to 0.049.

**
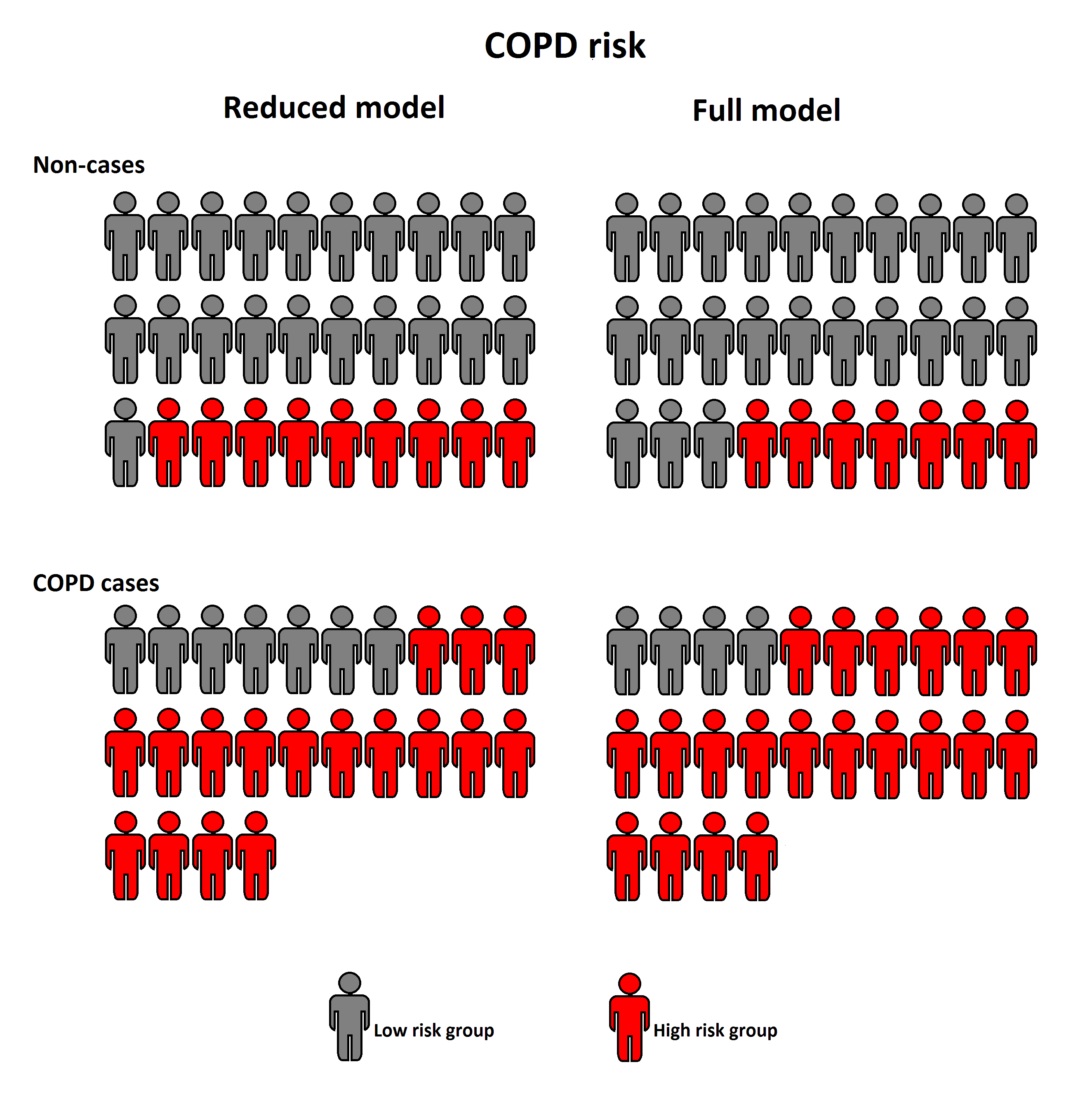
**

**Figure S14.** Predicted risk groups in the independent Generation Scotland: Scottish Family health study (GS:SFHS) test data (30 non-cases and 24 COPD cases) were defined as: low-risk group < 60% and higher-risk group ≥ 60%. The risk score from the reduced model built in the training data from the discovery GS:SFHS cohort contains information on sex, age, age^2^, height, height^2^, smoking status [never, former and current smoker] and pack-years of smoking. The risk score from the full model contains information from the reduced model and differentially methylated sites associated with respiratory function and prevalent chronic obstructive pulmonary disease (COPD) in the discovery GS:SFHS data. Grey non-cases and red COPD cases denote correctly classified individuals.

**
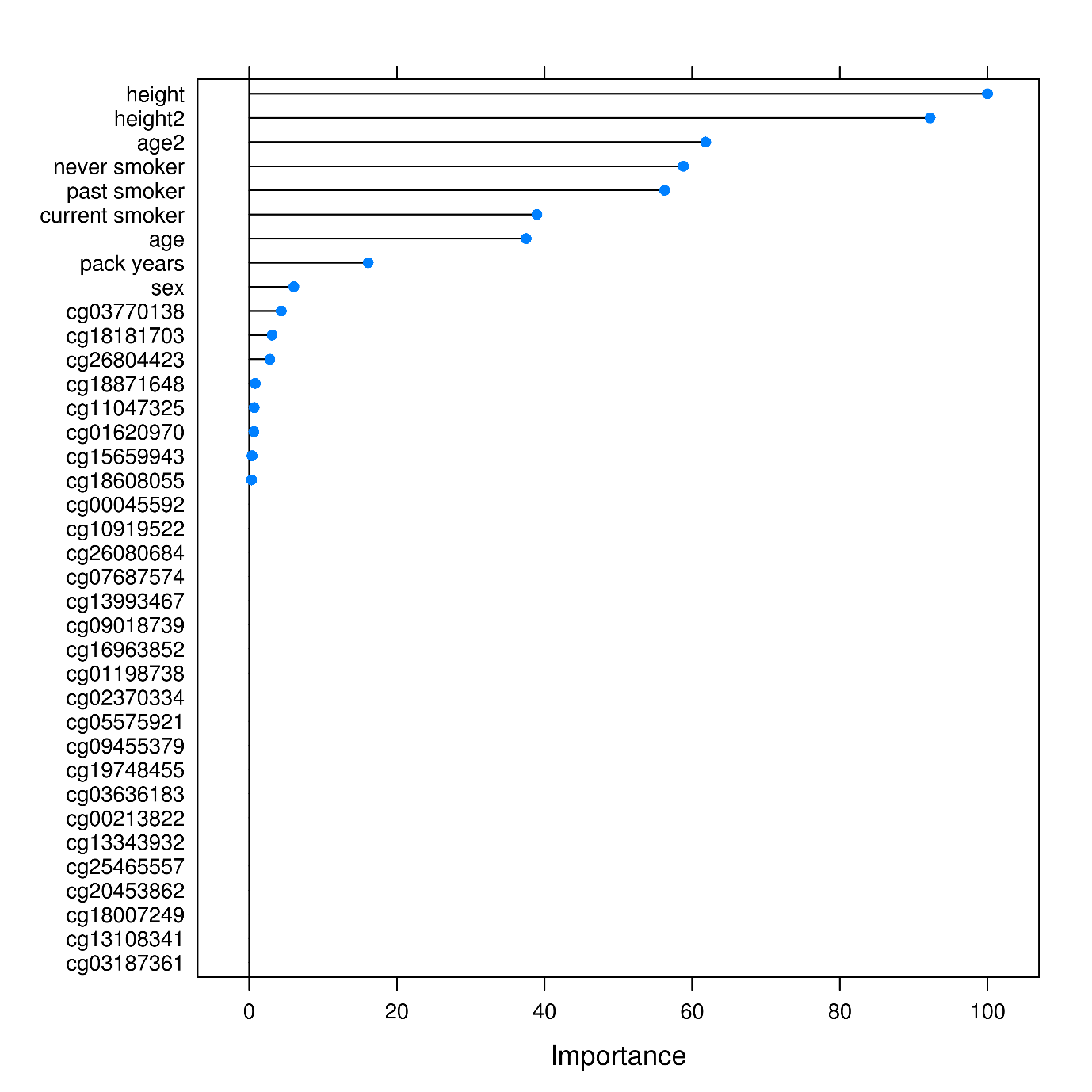
**

**Figure S15.** Variable importance plot for predictor variables from glmnet classifications used for predicting COPD risk in the independent test data from the discovery Generation Scotland: Scottish Family Health Survey (GS:SFHS) cohort data. The area under the ROC curve (AUC) was used as the ranking criterion. The importance value is proportional to the most important feature, which has an importance value of 100%. Higher values indicate variables that are more important to the classification.

**
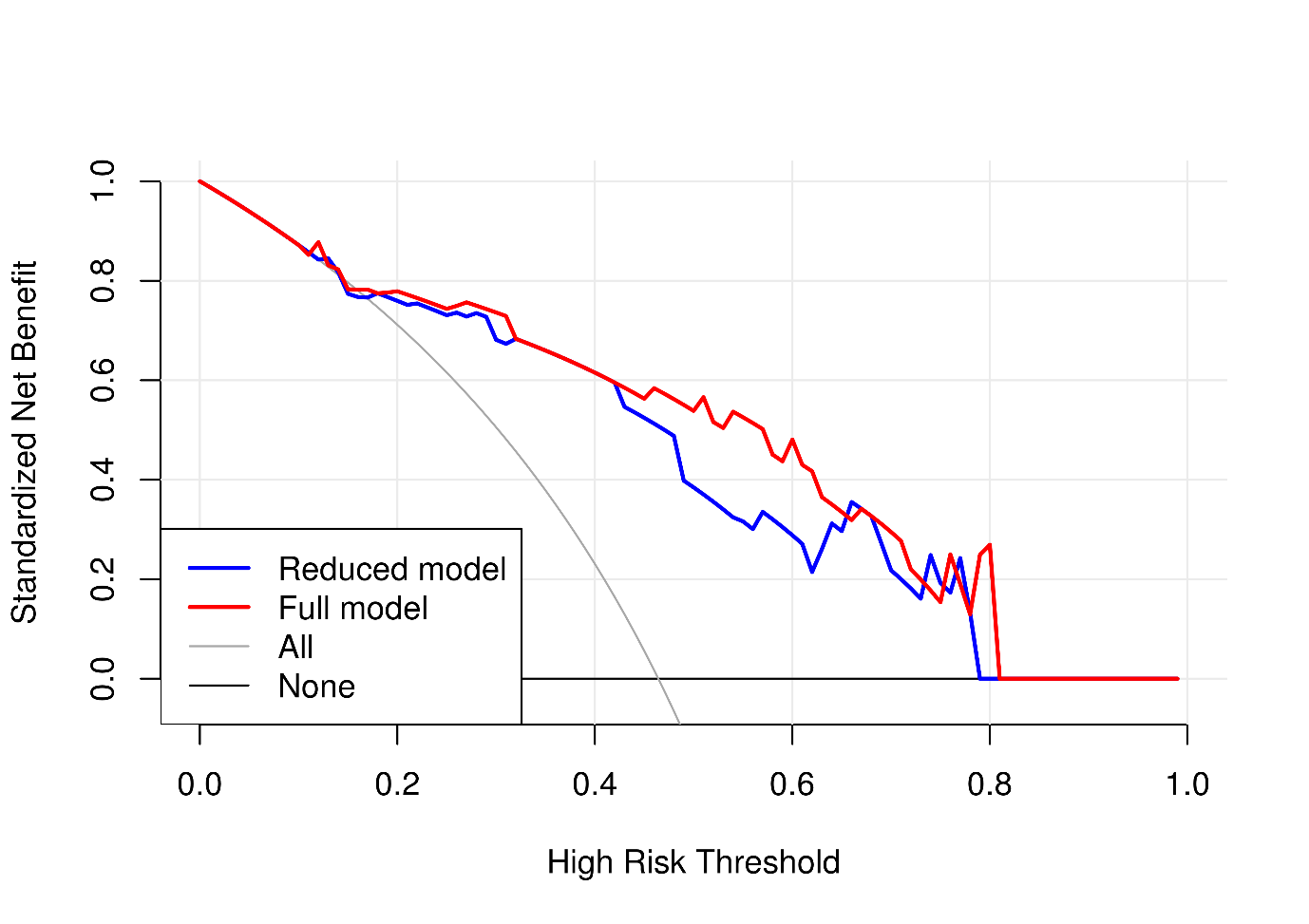
**

**Figure S16.** Decision curves for the reduced model (including sex, age, age^2^, height, height, height^2^, smoking status [never, former and current smoker] and pack-years of smoking) and full model (which added differentially methylated sites associated with respiratory function traits or COPD to the reduced model) to predict incident COPD in the independent test data from the discovery Generation Scotland: Scottish Family Health Study **(GS:SFHS) cohort**. The blue line is the net benefit (calculated by subtracting the proportions of false positive from the proportion of true positive, the former being weighted by the relative harms of false positive and false negative results) of using the reduced model, and the red line is the net benefit of the full model. The black and grey lines represent the net benefit of prognosing COPD ‘assuming none’ or ‘assuming all’ of the participants are at high-risk respectively.

**
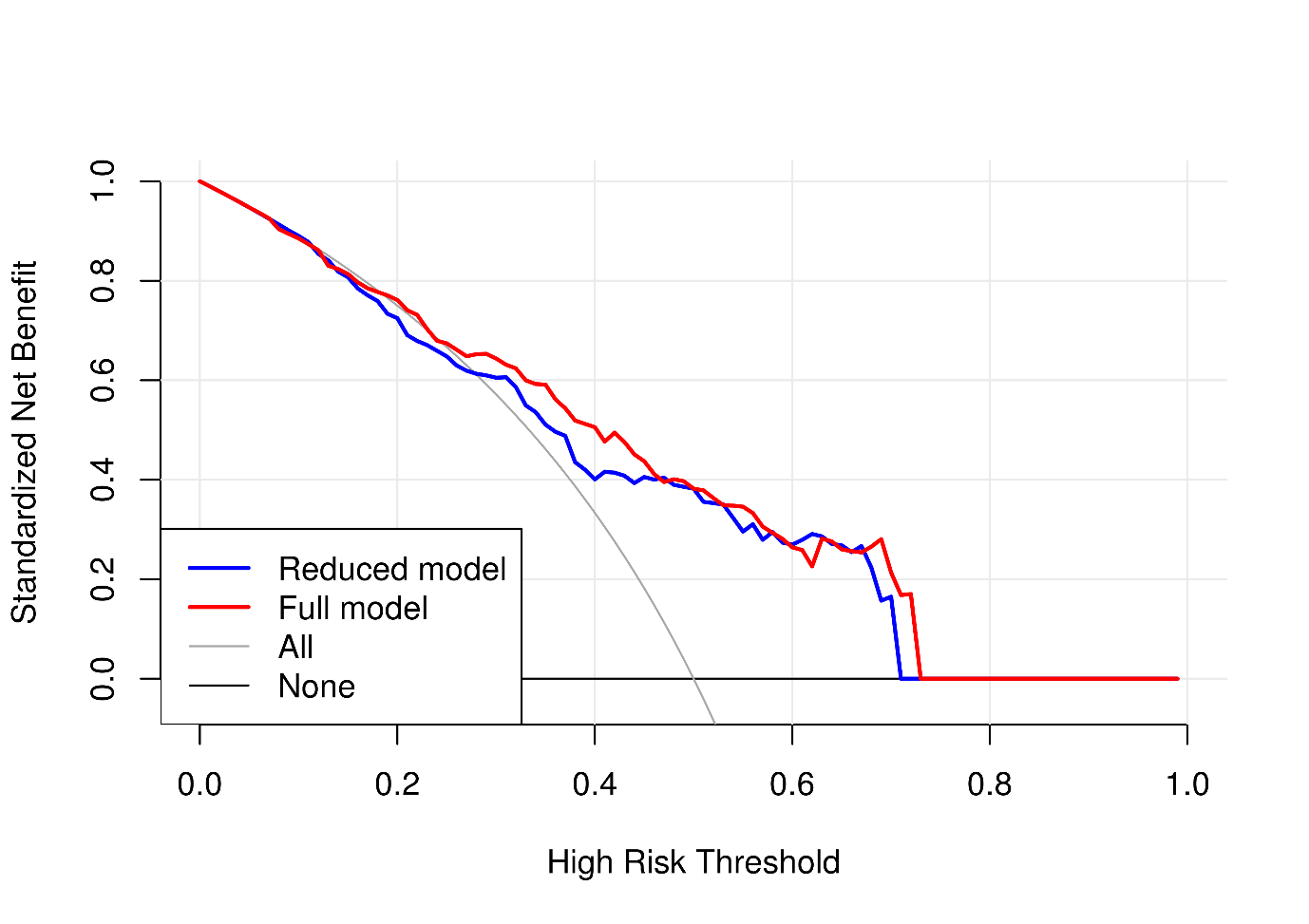
**

**Figure S17.** Decision curves for the reduced model (including sex, age, age^2^, height, height, height^2^, smoking status [never, former and current smoker] and pack-years of smoking) and full model (which added differentially methylated sites associated with respiratory function traits or COPD to the reduced model) built in the Generation Scotland: Scottish Family Health Study **(GS:SFHS) training data** to predict prevalent COPD in the independent data from the the Lothian Birth Cohort of 1936. The blue line is the net benefit (calculated by subtracting the proportions of false positive from the proportion of true positive, the former being weighted by the relative harms of false positive and false negative results) of using the reduced model, and the red line is the net benefit of the full model. The black and grey lines represent the net benefit of diagnosing COPD ‘assuming none’ or ‘assuming all’ of the participants are at high-risk respectively.


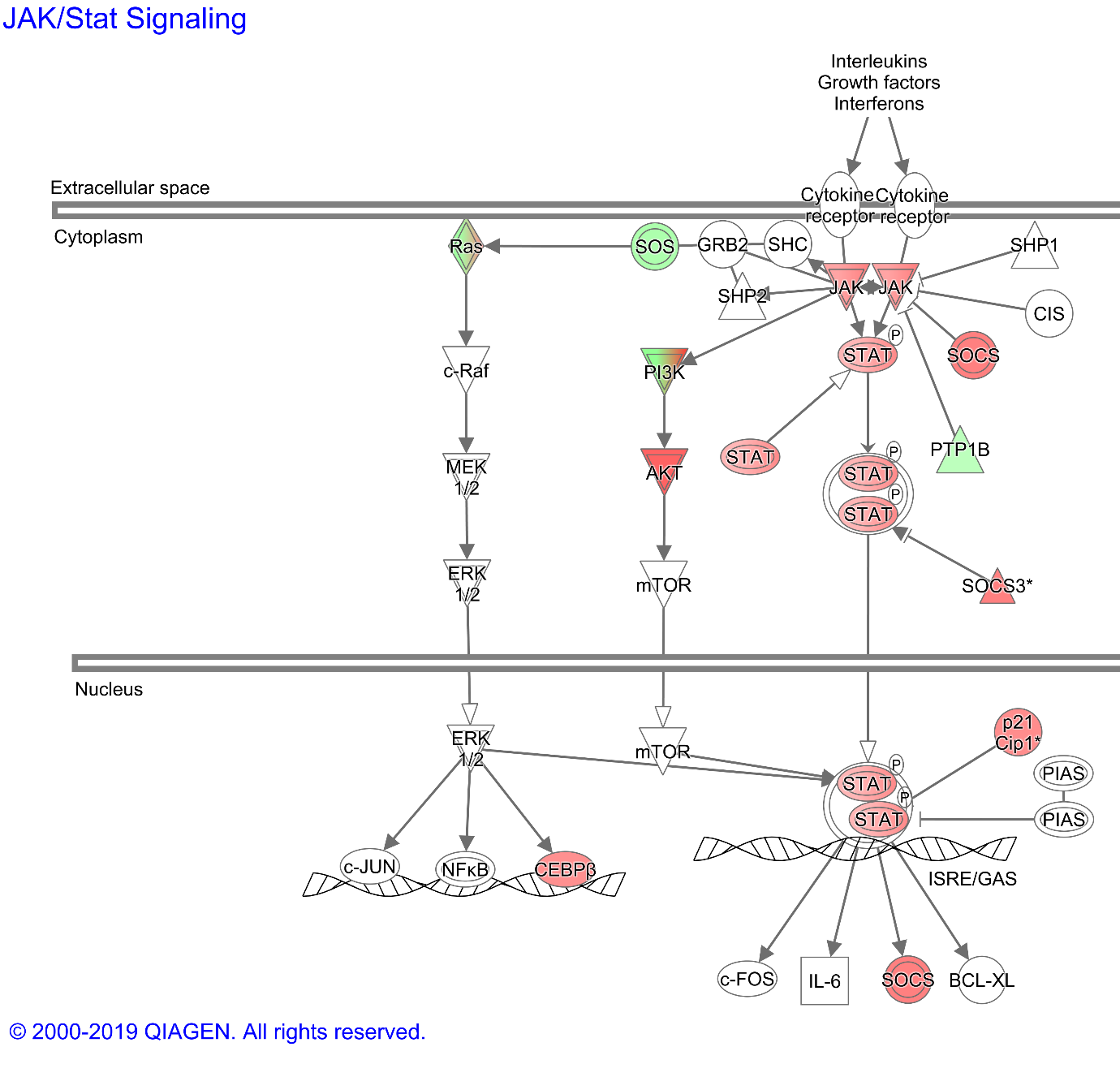


**Figure S18. Differentially methylated GpG sites associated with** forced expiratory volume in one second (FEV_1_**) enriched in genes within the JAK/Stat signalling pathway** from the discovery Generation Scotland: Scottish Family Health Survey (GS:SFHS) cohort data. DNA methylation probes were filtered at p<0.001. Relative changes in DNA methylation are depicted by graded shades of colour coding: red hypermethylation and green hypomethylation, grey no change. Direct interactions between molecules are depicted by solid lines. The genome-wide significant CpG sites (~3.6×10^−8^)^33^ in SOCS3 are shown as strongly (intense red colouring) hypermethylated within the pathway. Asterisks (*) indicate that multiple CpG sites in the dataset file map to a single gene in the pathway. This figure has been generated using Ingenuity Pathways Analysis (IPA; Ingenuity Systems, Inc., Cambridge, MA, USA).


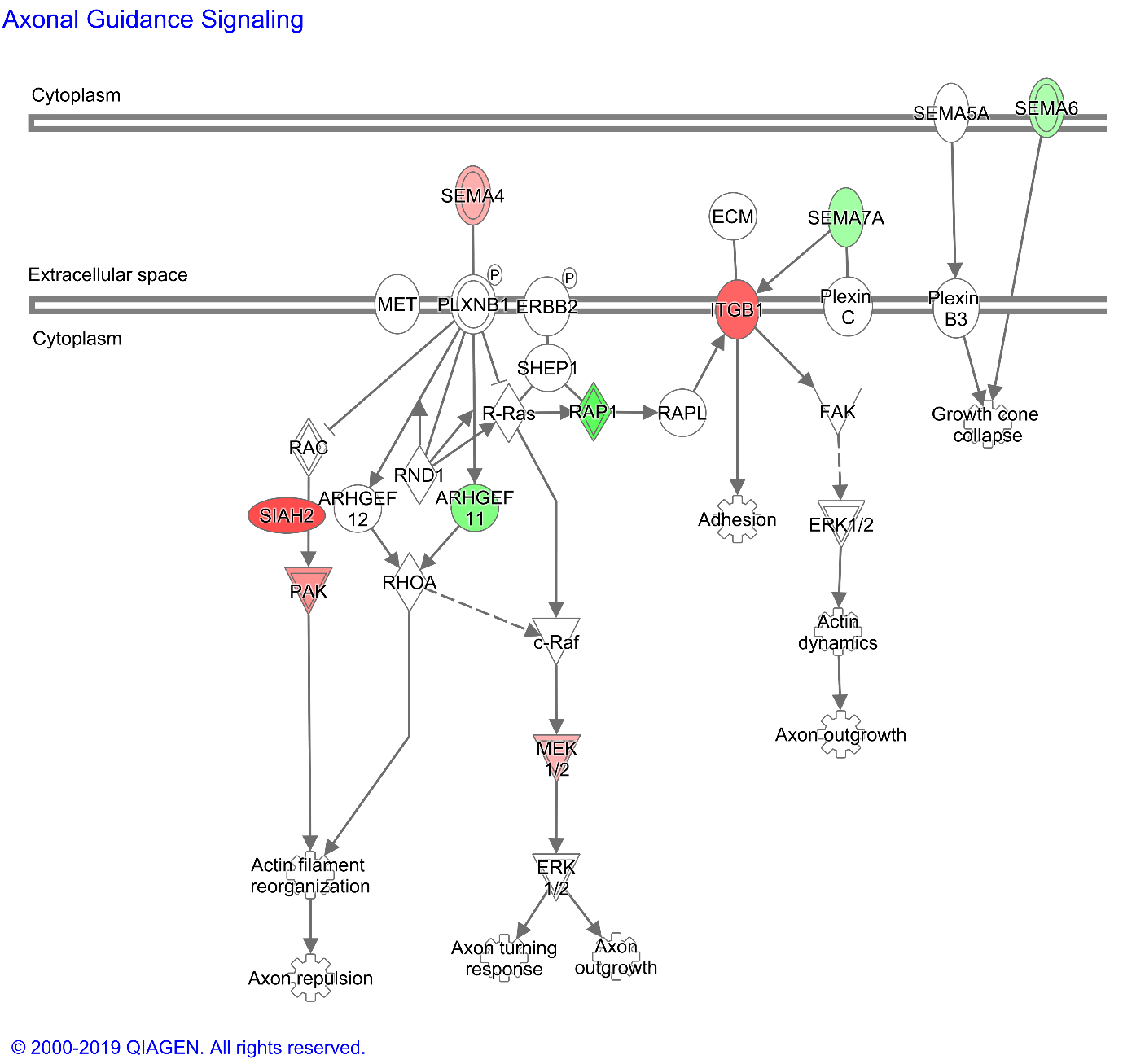


**Figure S19. Differentially methylated genes in the Axon Guidance signalling pathway associated with** forced vital capacity (FVC) ratio from the discovery Generation Scotland: Scottish Family Health Survey (GS:SFHS) cohort data. DNA methylation probes were filtered at p<0.001. Relative changes in DNA methylation are depicted by graded shades of colour coding: red hypermethylation and green hypomethylation, white no change or not applicable. Direct and indirect interactions between molecules are depicted by solid and dotted lines, respectively. The genome-wide significant CpG site (~3.6×10^−8^)^33^ in SIAH2 is hypermethylated (intense red colouring) within the pathway. Asterisks (*) indicate that multiple CpG sites in the dataset file map to a single gene in the pathway. This figure has been generated using Ingenuity Pathways Analysis (IPA; Ingenuity Systems, Inc., Cambridge, MA, USA).

**References**

1 Smith BH, Campbell A, Linksted P, *et al.* Cohort Profile: Generation Scotland: Scottish Family Health Study (GS:SFHS). The study, its participants and their potential for genetic research on health and illness. *Int J Epidemiol* 2013; **42**: 689–700.

2 Smith BH, Campbell H, Blackwood D, *et al.* Generation Scotland: the Scottish Family Health Study; a new resource for researching genes and heritability. *BMC Med Genet* 2006; **7**: 74.

3 Deary IJ, Gow AJ, Pattie A, Starr JM. Cohort profile: the Lothian Birth Cohorts of 1921 and 1936. *Int J Epidemiol* 2012; **41**: 1576–1584.

4 Taylor AM, Pattie A, Deary IJ. Cohort profile update: the lothian birth cohorts of 1921 and 1936. *Int J Epidemiol* 2018; published online March 12. DOI:10.1093/ije/dyy022.

5 Kerr SM, Campbell A, Murphy L, *et al.* Pedigree and genotyping quality analyses of over 10,000 DNA samples from the Generation Scotland: Scottish Family Health Study. *BMC Med Genet* 2013; **14**: 38.

6 Shah S, McRae AF, Marioni RE, *et al.* Genetic and environmental exposures constrain epigenetic drift over the human life course. *Genome Res* 2014; **24**: 1725–1733.

7 Navrady LB, Wolters MK, MacIntyre DJ, *et al.* Cohort Profile: Stratifying Resilience and Depression Longitudinally (STRADL): a questionnaire follow-up of Generation Scotland: Scottish Family Health Study (GS:SFHS). *Int J Epidemiol* 2018; **47**: 13–14g.

8 Team RC. R: A language and environment for statistical computing. 2013.

9 Aryee MJ, Jaffe AE, Corrada-Bravo H, *et al.* Minfi: a flexible and comprehensive Bioconductor package for the analysis of Infinium DNA methylation microarrays. *Bioinformatics* 2014; **30**: 1363–1369.

10 Fortin J-P, Fertig E, Hansen K. shinyMethyl: interactive quality control of Illumina 450k DNA methylation arrays in R. [version 2; referees: 2 approved]. *F1000Res* 2014; **3**: 175.

11 Schalkwyk LC, Pidsley R, Wong CCY, Toulemeimat N. wateRmelon: Illumina 450 methylation array normalization and metrics. .

12 Morrow JD, Cho MH, Hersh CP, *et al.* DNA methylation profiling in human lung tissue identifies genes associated with COPD. *Epigenetics* 2016; **11**: 1–10.

13 Qiu W, Baccarelli A, Carey VJ, *et al.* Variable DNA methylation is associated with chronic obstructive pulmonary disease and lung function. *Am J Respir Crit Care Med* 2012; **185**: 373–381.

14 Du P, Zhang X, Huang C-C, *et al.* Comparison of Beta-value and M-value methods for quantifying methylation levels by microarray analysis. *BMC Bioinformatics* 2010; **11**: 587.

15 1000 Genomes Project Consortium, Abecasis GR, Auton A, *et al.* An integrated map of genetic variation from 1,092 human genomes. *Nature* 2012; **491**: 56–65.

16 McCartney DL, Walker RM, Morris SW, McIntosh AM, Porteous DJ, Evans KL. Identification of polymorphic and off-target probe binding sites on the Illumina Infinium MethylationEPIC BeadChip. *Genom Data* 2016; **9**: 22–24.

17 Zhou W, Laird PW, Shen H. Comprehensive characterization, annotation and innovative use of Infinium DNA methylation BeadChip probes. *Nucleic Acids Res* 2017; **45**: e22.

18 Jaffe AE, Irizarry RA. Accounting for cellular heterogeneity is critical in epigenome-wide association studies. *Genome Biol* 2014; **15**: R31.

19 Houseman EA, Accomando WP, Koestler DC, *et al.* DNA methylation arrays as surrogate measures of cell mixture distribution. *BMC Bioinformatics* 2012; **13**: 86.

20 Kerr SM, Campbell A, Marten J, *et al.* Electronic health record and genome-wide genetic data in Generation Scotland participants. [version 1; referees: 2 approved, 1 approved with reservations]. *Wellcome Open Res* 2017; **2**: 85.

21 Yang J, Lee SH, Goddard ME, Visscher PM. GCTA: a tool for genome-wide complex trait analysis. *Am J Hum Genet* 2011; **88**: 76–82.

22 Canela-Xandri O, Law A, Gray A, Woolliams JA, Tenesa A. A new tool called DISSECT for analysing large genomic data sets using a Big Data approach. *Nat Commun* 2015; **6**: 10162.

23 Marioni RE, Shah S, McRae AF, *et al.* The epigenetic clock is correlated with physical and cognitive fitness in the Lothian Birth Cohort 1936. *Int J Epidemiol* 2015; **44**: 1388–1396.

24 Soler Artigas M, Wain LV, Repapi E, *et al.* Effect of five genetic variants associated with lung function on the risk of chronic obstructive lung disease, and their joint effects on lung function. *Am J Respir Crit Care Med* 2011; **184**: 786–795.

25 Jackson VE, Ntalla I, Sayers I, *et al.* Exome-wide analysis of rare coding variation identifies novel associations with COPD and airflow limitation in MOCS3, IFIT3 and SERPINA12. *Thorax* 2016; **71**: 501–509.

26 Howard DM, Adams MJ, Clarke T-K, *et al.* Haplotype-based association analysis of general cognitive ability in Generation Scotland, the English Longitudinal Study of Ageing, and UK Biobank. [version 1; referees: 2 approved]. *Wellcome Open Res* 2017; **2**: 61.

27 Hankinson JL, Odencrantz JR, Fedan KB. Spirometric reference values from a sample of the general U.S. population. *Am J Respir Crit Care Med* 1999; **159**: 179–187.

28 GOLD. Pocket Guide to COPD Diagnosis, Management, and Prevention – 2017. goldcopd.org, 2017.

29 Repapi E, Sayers I, Wain LV, *et al.* Genome-wide association study identifies five loci associated with lung function. *Nat Genet* 2010; **42**: 36–44.

30 Aulchenko YS, Ripke S, Isaacs A, van Duijn CM. GenABEL: an R library for genome-wide association analysis. *Bioinformatics* 2007; **23**: 1294–1296.

31 Lehne B, Drong AW, Loh M, *et al.* A coherent approach for analysis of the Illumina HumanMethylation450 BeadChip improves data quality and performance in epigenome-wide association studies. *Genome Biol* 2015; **16**: 37.

32 Ritchie ME, Phipson B, Wu D, *et al.* limma powers differential expression analyses for RNA-sequencing and microarray studies. *Nucleic Acids Res* 2015; **43**: e47.

33 Saffari A, Silver MJ, Zavattari P, *et al.* Estimation of a significance threshold for epigenome-wide association studies. *Genet Epidemiol* 2018; **42**: 20–33.

34 Bell JT, Pai AA, Pickrell JK, *et al.* DNA methylation patterns associate with genetic and gene expression variation in HapMap cell lines. *Genome Biol* 2011; **12**: R10.

35 Martin TC, Yet I, Tsai P-C, Bell JT. coMET: visualisation of regional epigenome-wide association scan results and DNA co-methylation patterns. *BMC Bioinformatics* 2015; **16**: 131.

36 Lozano R, Naghavi M, Foreman K, *et al.* Global and regional mortality from 235 causes of death for 20 age groups in 1990 and 2010: a systematic analysis for the Global Burden of Disease Study 2010. *Lancet* 2012; **380**: 2095–2128.

37 Guida F, Sandanger TM, Castagné R, *et al.* Dynamics of smoking-induced genome-wide methylation changes with time since smoking cessation. *Hum Mol Genet* 2015; **24**: 2349–2359.

38 Teschendorff AE, Yang Z, Wong A, *et al.* Correlation of Smoking-Associated DNA Methylation Changes in Buccal Cells With DNA Methylation Changes in Epithelial Cancer. *JAMA Oncol* 2015; **1**: 476–485.

39 Qiu W, Wan E, Morrow J, *et al.* The impact of genetic variation and cigarette smoke on DNA methylation in current and former smokers from the COPDGene study. *Epigenetics* 2015; **10**: 1064–1073.

40 Hillemacher T, Frieling H, Moskau S, *et al.* Global DNA methylation is influenced by smoking behaviour. *Eur Neuropsychopharmacol* 2008; **18**: 295–298.

41 Bell JT, Tsai P-C, Yang T-P, *et al.* Epigenome-wide scans identify differentially methylated regions for age and age-related phenotypes in a healthy ageing population. *PLoS Genet* 2012; **8**: e1002629.

42 Lee MK, Hong Y, Kim S-Y, Kim WJ, London SJ. Epigenome-wide association study of chronic obstructive pulmonary disease and lung function in Koreans. *Epigenomics* 2017; **9**: 971–984.

43 de Vries M, van der Plaat DA, Vonk JM, Boezen HM. No association between DNA methylation and COPD in never and current smokers. *BMJ Open Respir Res* 2018; **5**: e000282.

44 Lange P, Celli B, Agustí A, *et al.* Lung-Function Trajectories Leading to Chronic Obstructive Pulmonary Disease. *N Engl J Med* 2015; **373**: 111–122.

45 van Oostrom SH, Engelfriet PM, Verschuren WMM, *et al.* Aging-related trajectories of lung function in the general population-The Doetinchem Cohort Study. *PLoS One* 2018; **13**: e0197250.

46 Allinson JP, Hardy R, Donaldson GC, Shaheen SO, Kuh D, Wedzicha JA. Combined Impact of Smoking and Early-Life Exposures on Adult Lung Function Trajectories. *Am J Respir Crit Care Med* 2017; **196**: 1021–1030.

47 Rajkumar P, Pattabi K, Vadivoo S, *et al.* A cross-sectional study on prevalence of chronic obstructive pulmonary disease (COPD) in India: rationale and methods. *BMJ Open* 2017; **7**: e015211.

48 Magnus MC, Henderson J, Tilling K, Howe LD, Fraser A. Independent and combined associations of maternal and own smoking with adult lung function and COPD. *Int J Epidemiol* 2018; **47**: 1855–1864.

49 Rasam SA, Vanjare NV. Static lung volume should be used to confirm restrictive lung disease. *Int J Chron Obstruct Pulmon Dis* 2016; **11**: 2157–2158.

50 Mooe T, Stenfors N. The Prevalence of COPD in Individuals with Acute Coronary Syndrome: A Spirometry-Based Screening Study. *COPD* 2015; **12**: 453–461.

51 Ahn RS, Garner C. A Case Study of Fixed-Effects and Random-Effects Meta-Analysis Models for Genome-Wide Association Studies in Celiac Disease. *Hum Hered* 2015; **80**: 51–61.

52 Higgins JPT, Thompson SG. Quantifying heterogeneity in a meta-analysis. *Stat Med* 2002; **21**: 1539–1558.

53 Huang DW, Sherman BT, Lempicki RA. Systematic and integrative analysis of large gene lists using DAVID bioinformatics resources. *Nat Protoc* 2009; **4**: 44–57.

54 Benjamini Y, Hochberg Y. Controlling the False Discovery Rate: A Practical and Powerful Approach to Multiple Testing. *Journal of the Royal Statistical Society Series B (Methodological)* 1995; **57**: 289–300.

55 Brandsma C-A, van den Berge M, Postma DS, *et al.* A large lung gene expression study identifying fibulin-5 as a novel player in tissue repair in COPD. *Thorax* 2015; **70**: 21–32.

56 Wijetunga NA, Johnston AD, Maekawa R, *et al.* SMITE: an R/Bioconductor package that identifies network modules by integrating genomic and epigenomic information. *BMC Bioinformatics* 2017; **18**: 41.

57 Leavey K, Wilson SL, Bainbridge SA, Robinson WP, Cox BJ. Epigenetic regulation of placental gene expression in transcriptional subtypes of preeclampsia. *Clin Epigenetics* 2018; **10**: 28.

58 Croft D, Mundo AF, Haw R, *et al.* The Reactome pathway knowledgebase. *Nucleic Acids Res* 2014; **42**: D472–7.

59 Bar-Joseph Z, Gerber GK, Lee TI, *et al.* Computational discovery of gene modules and regulatory networks. *Nat Biotechnol* 2003; **21**: 1337–1342.

60 Donaldson GC, Wedzicha JA. COPD exacerbations .1: Epidemiology. *Thorax* 2006; **61**: 164–168.

61 Pesce G. Mortality rates for chronic lower respiratory diseases in Italy from 1979 to 2010: an age-period-cohort analysis. *ERJ Open Research* 2016; **2**. DOI:10.1183/23120541.00093-2015.

62 Croft JB, Wheaton AG, Liu Y, *et al.* Urban-Rural County and State Differences in Chronic Obstructive Pulmonary Disease - United States, 2015. *MMWR Morb Mortal Wkly Rep* 2018; **67**: 205–211.

63 Davies NM, Taylor GMJ, Taylor AE, *et al.* The effects of prescribing varenicline on two-year health outcomes: an observational cohort study using electronic medical records. *Addiction* 2018; **113**: 1105–1116.

64 Liu Y, Sun J, Gou Y, *et al.* A Multicity Analysis of the Short-Term Effects of Air Pollution on the Chronic Obstructive Pulmonary Disease Hospital Admissions in Shandong, China. *Int J Environ Res Public Health* 2018; **15**: 774.

65 Mo Z, Fu Q, Zhang L, *et al.* Acute effects of air pollution on respiratory disease mortalities and outpatients in Southeastern China. *Sci Rep* 2018; **8**: 3461.

66 Petersen JD, Siersma VD, Christensen R dePont, Storsveen MM, Nielsen CT, Waldorff FB. The risk of fall accidents for home dwellers with dementia-A register- and population-based case-control study. *Alzheimers Dement (Amst)* 2018; **10**: 421–428.

67 Poder TG, Carrier N, Bélanger M, *et al.* Eosinophil counts in first COPD hospitalizations: a 1-year cost analysis in Quebec, Canada. *Int J Chron Obstruct Pulmon Dis* 2018; **13**: 3065–3076.

68 Salimi F, Morgan G, Rolfe M, *et al.* Long-term exposure to low concentrations of air pollutants and hospitalisation for respiratory diseases: A prospective cohort study in Australia. *Environ Int* 2018; **121**: 415–420.

69 Ulrik CS, Lophaven SN, Andersen ZJ, Sørensen TI, Baker JL. BMI at school age and incident asthma admissions in early adulthood: a prospective study of 310,211 children. *Clin Epidemiol* 2018; **10**: 605–612.

70 Zaigham S, Johnson L, Wollmer P, Engström G. Measures of low lung function and the prediction of incident COPD events and acute coronary events. *Respir Med* 2018; **144**: 68–73.

71 Wittbrodt ET, Millette LA, Evans KA, Bonafede M, Tkacz J, Ferguson GT. Differences in health care outcomes between postdischarge COPD patients treated with inhaled corticosteroid/long-acting β2-agonist via dry-powder inhalers and pressurized metered-dose inhalers. *Int J Chron Obstruct Pulmon Dis* 2019; **14**: 101–114.

72 Branco P, Torgo L, Ribeiro RP. A survey of predictive modeling on imbalanced domains. *ACM Comput Surv* 2016; **49**: 1–50.

73 Daskalaki S, Kopanas I, Avouris N. Evaluation of classifiers for an uneven class distribution problem. *Applied Artificial Intelligence* 2006; **20**: 381–417.

74 Forman G, Scholz M. Apples-to-apples in cross-validation studies. *SIGKDD Explor Newsl* 2010; **12**: 49.

75 Chawla NV, Japkowicz N, Kotcz A. Editorial: special issue on learning from imbalanced data sets. *SIGKDD Explor Newsl* 2004; **6**: 1.

76 Li J, Fong S, Wong RK, Chu VW. Adaptive multi-objective swarm fusion for imbalanced data classification. *Information Fusion* 2018; **39**: 1–24.

77 García V, Mollineda RA, Sánchez JS. A bias correction function for classification performance assessment in two-class imbalanced problems. *Knowledge-Based Systems* 2014; **59**: 66–74.

78 Wilk JB, Shrine NRG, Loehr LR, *et al.* Genome-wide association studies identify CHRNA5/3 and HTR4 in the development of airflow obstruction. *Am J Respir Crit Care Med* 2012; **186**: 622–632.

79 Wain LV, Shrine N, Artigas MS, *et al.* Genome-wide association analyses for lung function and chronic obstructive pulmonary disease identify new loci and potential druggable targets. *Nat Genet* 2017; **49**: 416–425.

80 Li X, Ortega VE, Ampleford EJ, *et al.* Genome-wide association study of lung function and clinical implication in heavy smokers. *BMC Med Genet* 2018; **19**: 134.

81 Youden WJ. Index for rating diagnostic tests. *Cancer* 1950; **3**: 32–35.

82 Cook NR. Clinically relevant measures of fit? A note of caution. *Am J Epidemiol* 2012; **176**: 488–491.

83 Zaiou M, Bakillah A. Epigenetic Regulation of ATP-Binding Cassette Protein A1 (ABCA1) Gene Expression: A New Era to Alleviate Atherosclerotic Cardiovascular Disease. *Diseases* 2018; **6**. DOI:10.3390/diseases6020034.

84 Sun Y-H, He L, Yan M-Y, *et al.* Overexpression of GLP-1 receptors suppresses proliferation and cytokine release by airway smooth muscle cells of patients with chronic obstructive pulmonary disease via activation of ABCA1. *Mol Med Rep* 2017; **16**: 929–936.

85 Chen J-H, Zheng Y-L, Xu C-Q, *et al.* Valproic acid (VPA) enhances cisplatin sensitivity of non-small cell lung cancer cells via HDAC2 mediated down regulation of ABCA1. *Biol Chem* 2017; **398**: 785–792.

86 Oram J. Tangier disease and ABCA1. *Biochimica et Biophysica Acta (BBA) - Molecular and Cell Biology of Lipids* 2000; **1529**: 321–330.

87 Timmins JM, Lee J-Y, Boudyguina E, *et al.* Targeted inactivation of hepatic Abca1 causes profound hypoalphalipoproteinemia and kidney hypercatabolism of apoA-I. *J Clin Invest* 2005; **115**: 1333–1342.

88 Fehér Á, Giricz Z, Juhász A, Pákáski M, Janka Z, Kálmán J. ABCA1 rs2230805 and rs2230806 common gene variants are associated with Alzheimer’s disease. *Neurosci Lett* 2018; **664**: 79–83.

89 Bates SR, Tao J-Q, Collins HL, Francone OL, Rothblat GH. Pulmonary abnormalities due to ABCA1 deficiency in mice. *Am J Physiol Lung Cell Mol Physiol* 2005; **289**: L980–9.

90 van der Deen M, de Vries EGE, Timens W, Scheper RJ, Timmer-Bosscha H, Postma DS. ATP-binding cassette (ABC) transporters in normal and pathological lung. *Respir Res* 2005; **6**: 59.

91 Dai C, Yao X, Vaisman B, *et al.* ATP-binding cassette transporter 1 attenuates ovalbumin-induced neutrophilic airway inflammation. *Am J Respir Cell Mol Biol* 2014; **51**: 626–636.

92 Sonett J, Goldklang M, Sklepkiewicz P, *et al.* A critical role for ABC transporters in persistent lung inflammation in the development of emphysema after smoke exposure. *FASEB J* 2018; : fj201701381.

93 Willer CJ, Sanna S, Jackson AU, *et al.* Newly identified loci that influence lipid concentrations and risk of coronary artery disease. *Nat Genet* 2008; **40**: 161–169.

94 Edmondson AC, Braund PS, Stylianou IM, *et al.* Dense genotyping of candidate gene loci identifies variants associated with high-density lipoprotein cholesterol. *Circ Cardiovasc Genet* 2011; **4**: 145–155.

95 Hautefort A, Chesné J, Preussner J, *et al.* Pulmonary endothelial cell DNA methylation signature in pulmonary arterial hypertension. *Oncotarget* 2017; **8**: 52995–53016.

96 Dayeh T, Tuomi T, Almgren P, *et al.* DNA methylation of loci within ABCG1 and PHOSPHO1 in blood DNA is associated with future type 2 diabetes risk. *Epigenetics* 2016; **11**: 482–488.

97 Guay S-P, Légaré C, Houde A-A, Mathieu P, Bossé Y, Bouchard L. Acetylsalicylic acid, aging and coronary artery disease are associated with ABCA1 DNA methylation in men. *Clin Epigenetics* 2014; **6**: 14.

98 Fernández-Sanlés A, Sayols-Baixeras S, Subirana I, Degano IR, Elosua R. Association between DNA methylation and coronary heart disease or other atherosclerotic events: A systematic review. *Atherosclerosis* 2017; **263**: 325–333.

99 Ghaznavi H, Mahmoodi K, Soltanpour MS. A preliminary study of the association between the ABCA1 gene promoter DNA methylation and coronary artery disease risk. *Mol Biol Res Commun* 2018; **7**: 59–65.

100 Haase H, Podzuweit T, Lutsch G, *et al.* Signaling from beta-adrenoceptor to L-type calcium channel: identification of a novel cardiac protein kinase A target possessing similarities to AHNAK. *FASEB J* 1999; **13**: 2161–2172.

101 Gentil BJ, Delphin C, Mbele GO, *et al.* The giant protein AHNAK is a specific target for the calcium- and zinc-binding S100B protein: potential implications for Ca2+ homeostasis regulation by S100B. *J Biol Chem* 2001; **276**: 23253–23261.

102 Hohaus A, Person V, Behlke J, Schaper J, Morano I, Haase H. The carboxyl-terminal region of ahnak provides a link between cardiac L-type Ca2+ channels and the actin-based cytoskeleton. *FASEB J* 2002; **16**: 1205–1216.

103 Haase H. Ahnak, a new player in beta-adrenergic regulation of the cardiac L-type Ca2+ channel. *Cardiovasc Res* 2007; **73**: 19–25.

104 Shin JH, Kim IY, Kim YN, *et al.* Obesity Resistance and Enhanced Insulin Sensitivity in Ahnak-/- Mice Fed a High Fat Diet Are Related to Impaired Adipogenesis and Increased Energy Expenditure. *PLoS One* 2015; **10**: e0139720.

105 Nedeljkovic I, Terzikhan N, Vonk JM, *et al.* A Genome-Wide Linkage Study for Chronic Obstructive Pulmonary Disease in a Dutch Genetic Isolate Identifies Novel Rare Candidate Variants. *Front Genet* 2018; **9**: 133.

106 Awasthi S, Maity T, Oyler BL, *et al.* Quantitative targeted proteomic analysis of potential markers of tyrosine kinase inhibitor (TKI) sensitivity in EGFR mutated lung adenocarcinoma. *J Proteomics* 2018; **189**: 48–59.

107 Park JW, Kim IY, Choi JW, *et al.* AHNAK loss in mice promotes type II pneumocyte hyperplasia and lung tumor development. *Mol Cancer Res* 2018; **16**: 1287–1298.

108 Ungvári I, Hullám G, Antal P, *et al.* Evaluation of a partial genome screening of two asthma susceptibility regions using bayesian network based bayesian multilevel analysis of relevance. *PLoS One* 2012; **7**: e33573.

109 Fonseca AL, Kugelberg J, Starker LF, *et al.* Comprehensive DNA methylation analysis of benign and malignant adrenocortical tumors. *Genes Chromosomes Cancer* 2012; **51**: 949–960.

110 Kasai A, Hiramatsu N, Hayakawa K, Yao J, Maeda S, Kitamura M. High levels of dioxin-like potential in cigarette smoke evidenced by in vitro and in vivo biosensing. *Cancer Res* 2006; **66**: 7143–7150.

111 Zudaire E, Cuesta N, Murty V, *et al.* The aryl hydrocarbon receptor repressor is a putative tumor suppressor gene in multiple human cancers. *J Clin Invest* 2008; **118**: 640–650.

112 Zhang Y, Elgizouli M, Schöttker B, Holleczek B, Nieters A, Brenner H. Smoking-associated DNA methylation markers predict lung cancer incidence. *Clin Epigenetics* 2016; **8**: 127.

113 Park SL, Patel YM, Loo LWM, *et al.* Association of internal smoking dose with blood DNA methylation in three racial/ethnic populations. *Clin Epigenetics* 2018; **10**: 110.

114 Richmond RC, Suderman M, Langdon R, Relton CL, Davey Smith G. DNA methylation as a marker for prenatal smoke exposure in adults. *Int J Epidemiol* 2018; **47**: 1120–1130.

115 Bojesen SE, Timpson N, Relton C, Davey Smith G, Nordestgaard BG. AHRR (cg05575921) hypomethylation marks smoking behaviour, morbidity and mortality. *Thorax* 2017; **72**: 646–653.

116 Kodal JB, Kobylecki CJ, Vedel-Krogh S, Nordestgaard BG, Bojesen SE. AHRR hypomethylation, lung function, lung function decline and respiratory symptoms. *Eur Respir J* 2018; **51**. DOI:10.1183/13993003.01512-2017.

117 Baglietto L, Ponzi E, Haycock P, *et al.* DNA methylation changes measured in pre-diagnostic peripheral blood samples are associated with smoking and lung cancer risk. *Int J Cancer* 2017; **140**: 50–61.

118 Alhamdow A, Lindh C, Hagberg J, *et al.* DNA methylation of the cancer-related genes F2RL3 and AHRR is associated with occupational exposure to polycyclic aromatic hydrocarbons. *Carcinogenesis* 2018; **39**: 869–878.

119 Agardh E, Lundstig A, Perfilyev A, *et al.* Genome-wide analysis of DNA methylation in subjects with type 1 diabetes identifies epigenetic modifications associated with proliferative diabetic retinopathy. *BMC Med* 2015; **13**: 182.

120 Zhang Y, Schöttker B, Florath I, *et al.* Smoking-Associated DNA Methylation Biomarkers and Their Predictive Value for All-Cause and Cardiovascular Mortality. *Environ Health Perspect* 2016; **124**: 67–74.

121 Safran M, Dalah I, Alexander J, *et al.* GeneCards Version 3: the human gene integrator. *Database (Oxford)* 2010; **2010**: baq020.

122 Li Y, Rong G, Kang H. Taxotere-induced elevated expression of IL8 in carcinoma-associated fibroblasts of breast invasive ductal cancer. *Oncol Lett* 2017; **13**: 1856–1860.

123 Gao X, Gào X, Zhang Y, Breitling LP, Schöttker B, Brenner H. Associations of self-reported smoking, cotinine levels and epigenetic smoking indicators with oxidative stress among older adults: a population-based study. *Eur J Epidemiol* 2017; **32**: 443–456.

124 Walaszczyk E, Luijten M, Spijkerman AMW, *et al.* DNA methylation markers associated with type 2 diabetes, fasting glucose and HbA1c levels: a systematic review and replication in a case-control sample of the Lifelines study. *Diabetologia* 2018; **61**: 354–368.

125 Yates LL, Schnatwinkel C, Murdoch JN, *et al.* The PCP genes Celsr1 and Vangl2 are required for normal lung branching morphogenesis. *Hum Mol Genet* 2010; **19**: 2251–2267.

126 Curtin JA, Quint E, Tsipouri V, *et al.* Mutation of Celsr1 disrupts planar polarity of inner ear hair cells and causes severe neural tube defects in the mouse. *Curr Biol* 2003; **13**: 1129–1133.

127 Lei Y, Zhu H, Yang W, Ross ME, Shaw GM, Finnell RH. Identification of novel CELSR1 mutations in spina bifida. *PLoS One* 2014; **9**: e92207.

128 Hardin M, Cho MH, Sharma S, *et al.* Sex-Based Genetic Association Study Identifies CELSR1 as a Possible Chronic Obstructive Pulmonary Disease Risk Locus among Women. *Am J Respir Cell Mol Biol* 2017; **56**: 332–341.

129 McDonald M-LN, Won S, Mattheisen M, *et al.* Body mass index change in gastrointestinal cancer and chronic obstructive pulmonary disease is associated with Dedicator of Cytokinesis 1. *J Cachexia Sarcopenia Muscle* 2017; **8**: 428–436.

130 Villanueva A, Portela A, Sayols S, *et al.* DNA methylation-based prognosis and epidrivers in hepatocellular carcinoma. *Hepatology* 2015; **61**: 1945–1956.

131 Zuko A, Bouyain S, van der Zwaag B, Burbach JPH. Contactins: structural aspects in relation to developmental functions in brain disease. *Adv Protein Chem Struct Biol* 2011; **84**: 143–180.

132 Miura S, Shibata H, Furuya H, *et al.* The contactin 4 gene locus at 3p26 is a candidate gene of SCA16. *Neurology* 2006; **67**: 1236–1241.

133 Fernandez T, Morgan T, Davis N, *et al.* Disruption of contactin 4 (CNTN4) results in developmental delay and other features of 3p deletion syndrome. *Am J Hum Genet* 2004; **74**: 1286–1293.

134 Clark SL, Aberg KA, Nerella S, *et al.* Combined whole methylome and genomewide association study implicates CNTN4 in alcohol use. *Alcohol Clin Exp Res* 2015; **39**: 1396–1405.

135 Kim Y-H, Lee HC, Kim S-Y, *et al.* Epigenomic analysis of aberrantly methylated genes in colorectal cancer identifies genes commonly affected by epigenetic alterations. *Ann Surg Oncol* 2011; **18**: 2338–2347.

136 Wu X, Rauch TA, Zhong X, *et al.* CpG island hypermethylation in human astrocytomas. *Cancer Res* 2010; **70**: 2718–2727.

137 Alvarez-Garcia O, Fisch KM, Wineinger NE, *et al.* Increased DNA methylation and reduced expression of transcription factors in human osteoarthritis cartilage. *Arthritis Rheumatol* 2016; **68**: 1876–1886.

138 Xu K, Zhang X, Wang Z, Hu Y, Sinha R. Epigenome-wide association analysis revealed that SOCS3 methylation influences the effect of cumulative stress on obesity. *Biol Psychol* 2018; **131**: 63–71.

139 Geremek M, Ziętkiewicz E, Bruinenberg M, *et al.* Ciliary genes are down-regulated in bronchial tissue of primary ciliary dyskinesia patients. *PLoS One* 2014; **9**: e88216.

140 Dizier M-H, Nadif R, Margaritte-Jeannin P, *et al.* Interaction between the DNAH9 gene and early smoke exposure in bronchial hyperresponsiveness. *Eur Respir J* 2016; **47**: 1072–1081.

141 Kusakabe M, Kutomi T, Watanabe K, *et al.* Identification of G0S2 as a gene frequently methylated in squamous lung cancer by combination of in silico and experimental approaches. *Int J Cancer* 2010; **126**: 1895–1902.

142 Zhang Y, Yang R, Burwinkel B, Breitling LP, Brenner H. F2RL3 methylation as a biomarker of current and lifetime smoking exposures. *Environ Health Perspect* 2014; **122**: 131–137.

143 Li S, Wong EM, Bui M, *et al.* Causal effect of smoking on DNA methylation in peripheral blood: a twin and family study. *Clin Epigenetics* 2018; **10**: 18.

144 Zhang Y, Schöttker B, Ordóñez-Mena J, *et al.* F2RL3 methylation, lung cancer incidence and mortality. *Int J Cancer* 2015; **137**: 1739–1748.

145 Fasanelli F, Baglietto L, Ponzi E, *et al.* Hypomethylation of smoking-related genes is associated with future lung cancer in four prospective cohorts. *Nat Commun* 2015; **6**: 10192.

146 Wahl A, Kasela S, Carnero-Montoro E, *et al.* IgG glycosylation and DNA methylation are interconnected with smoking. *Biochim Biophys Acta* 2018; **1862**: 637–648.

147 Breitling LP, Salzmann K, Rothenbacher D, Burwinkel B, Brenner H. Smoking, F2RL3 methylation, and prognosis in stable coronary heart disease. *Eur Heart J* 2012; **33**: 2841–2848.

148 Breitling LP. Current genetics and epigenetics of smoking/tobacco-related cardiovascular disease. *Arterioscler Thromb Vasc Biol* 2013; **33**: 1468–1472.

149 Meng X, Lu X, Morris CA, Keating MT. A novel human gene FKBP6 is deleted in Williams syndrome. *Genomics* 1998; **52**: 130–137.

150 Singh V, Bansal SK, Singh R, Singh K. Autosomal genes in male infertility. In: Singh R, Singh K, eds. Male infertility: understanding, causes and treatment. Singapore: Springer Singapore, 2017: 231–252.

151 Ni B, Lin Y, Sun L, *et al.* Low-frequency germline variants across 6p22.2-6p21.33 are associated with non-obstructive azoospermia in Han Chinese men. *Hum Mol Genet* 2015; **24**: 5628–5636.

152 Brebi P, Maldonado L, Noordhuis MG, *et al.* Genome-wide methylation profiling reveals Zinc finger protein 516 (ZNF516) and FK-506-binding protein 6 (FKBP6) promoters frequently methylated in cervical neoplasia, associated with HPV status and ethnicity in a Chilean population. *Epigenetics* 2014; **9**: 308–317.

153 Pras E, Raz J, Yahalom V, *et al.* A Nonsense Mutation in the Glucosaminyl (*N* -acetyl) Transferase 2 Gene (*GCNT2* ): Association with Autosomal Recessive Congenital Cataracts. *Invest Ophthalmol Vis Sci* 2004; **45**: 1940.

154 Lessard S, Beaudoin M, Benkirane K, Lettre G. Comparison of DNA methylation profiles in human fetal and adult red blood cell progenitors. *Genome Med* 2015; **7**: 1.

155 Murakami M, Yoshimoto T, Nakabayashi K, *et al.* Integration of transcriptome and methylome analysis of aldosterone-producing adenomas. *Eur J Endocrinol* 2015; **173**: 185–195.

156 Nakamura K, Yamashita K, Sawaki H, *et al.* Aberrant methylation of GCNT2 is tightly related to lymph node metastasis of primary CRC. *Anticancer Res* 2015; **35**: 1411–1421.

157 Zhang H, Meng F, Wu S, *et al.* Engagement of I-branching {beta}-1, 6-N-acetylglucosaminyltransferase 2 in breast cancer metastasis and TGF-{beta} signaling. *Cancer Res* 2011; **71**: 4846–4856.

158 Gordon TP, Cavill D, Neufing P, Zhang YJ, Pietropaolo M. ICA69 autoantibodies in primary Sjögren’s syndrome. *Lupus* 2004; **13**: 483–484.

159 McRonald FE, Morris MR, Gentle D, *et al.* CpG methylation profiling in VHL related and VHL unrelated renal cell carcinoma. *Mol Cancer* 2009; **8**: 31.

160 Baarsma HA, Königshoff M. WNT-er is coming ’: WNT signalling in chronic lung diseases. *Thorax* 2017; **72**: 746–759.

161 Bochem AE, van Wijk DF, Holleboom AG, *et al.* ABCA1 mutation carriers with low high-density lipoprotein cholesterol are characterized by a larger atherosclerotic burden. *Eur Heart J* 2013; **34**: 286–291.

162 Brooks-Wilson A, Marcil M, Clee SM, *et al.* Mutations in ABC1 in Tangier disease and familial high-density lipoprotein deficiency. *Nat Genet* 1999; **22**: 336–345.

163 Wagh AD, Sharma M, Mahapatra J, Chatterjee A, Jain M, Addepalli V. Investigation into the Role of PI3K and JAK3 Kinase Inhibitors in Murine Models of Asthma. *Front Pharmacol* 2017; **8**: 82.

164 O’Shea JJ, Husa M, Li D, *et al.* Jak3 and the pathogenesis of severe combined immunodeficiency. *Mol Immunol* 2004; **41**: 727–737.

165 Korytina GF, Akhmadishina LZ, Kochetova OV, Aznabaeva YG, Zagidullin SZ, Victorova TV. Inflammatory and Immune Response Genes Polymorphisms are Associated with Susceptibility to Chronic Obstructive Pulmonary Disease in Tatars Population from Russia. *Biochem Genet* 2016; **54**: 388–412.

166 Bediaga NG, Acha-Sagredo A, Guerra I, *et al.* DNA methylation epigenotypes in breast cancer molecular subtypes. *Breast Cancer Res* 2010; **12**: R77.

167 López JI, Angulo JC, Martín A, *et al.* A DNA hypermethylation profile reveals new potential biomarkers for the evaluation of prognosis in urothelial bladder cancer. *APMIS* 2017; **125**: 787–796.

168 Cheng Y-L, Zhang X-H, Sun Y-W, *et al.* Genomewide DNA Methylation Responses in Patients with β-Thalassemia Treated with Yisui Shengxue Granules (). *Chin J Integr Med* 2018; published online May 3. DOI:10.1007/s11655-018-2777-9.

169 Hernandez-Vargas H, Gruffat H, Cros MP, *et al.* Viral driven epigenetic events alter the expression of cancer-related genes in Epstein-Barr-virus naturally infected Burkitt lymphoma cell lines. *Sci Rep* 2017; **7**: 5852.

170 Somineni HK, Kilaru V, Venkateswaran S, *et al.* Tu1796 - Epigenome-Wide Association Study Identified Shared Methylomic Contributions to Susceptibility and Progression in Pediatric Crohn’s Disease. *Gastroenterology* 2018; **154**: S–1022.

171 Weimann M, Grossmann A, Woodsmith J, *et al.* A Y2H-seq approach defines the human protein methyltransferase interactome. *Nat Methods* 2013; **10**: 339–342.

172 Davies SJ, Wise C, Venkatesh B, *et al.* Mapping of three translocation breakpoints associated with orofacial clefting within 6p24 and identification of new transcripts within the region. *Cytogenet Genome Res* 2004; **105**: 47–53.

173 Ohnishi T, Yamada K, Watanabe A, *et al.* Ablation of Mrds1/Ofcc1 induces hyper-γ-glutamyl transpeptidasemia without abnormal head development and schizophrenia-relevant behaviors in mice. *PLoS One* 2011; **6**: e29499.

174 Mattheisen M, Samuels JF, Wang Y, *et al.* Genome-wide association study in obsessive-compulsive disorder: results from the OCGAS. *Mol Psychiatry* 2015; **20**: 337–344.

175 Sundaram SK, Huq AM, Sun Z, *et al.* Exome sequencing of a pedigree with Tourette syndrome or chronic tic disorder. *Ann Neurol* 2011; **69**: 901–904.

176 Cukier HN, Dueker ND, Slifer SH, *et al.* Exome sequencing of extended families with autism reveals genes shared across neurodevelopmental and neuropsychiatric disorders. *Mol Autism* 2014; **5**: 1.

177 Synofzik M, Németh AH. Recessive ataxias. *Handb Clin Neurol* 2018; **155**: 73–89.

178 Lokk K, Vooder T, Kolde R, *et al.* Methylation markers of early-stage non-small cell lung cancer. *PLoS One* 2012; **7**: e39813.

179 Khor GH, Froemming GRA, Zain RB, Abraham MT, Thong KL. Screening of differential promoter hypermethylated genes in primary oral squamous cell carcinoma. *Asian Pac J Cancer Prev* 2014; **15**: 8957–8961.

180 Senga T, Iwamoto T, Kitamura T, Miyake Y, Hamaguchi M. JAK/STAT3-dependent activation of the RalGDS/Ral pathway in M1 mouse myeloid leukemia cells. *J Biol Chem* 2001; **276**: 32678–32681.

181 González-García A, Pritchard CA, Paterson HF, Mavria G, Stamp G, Marshall CJ. RalGDS is required for tumor formation in a model of skin carcinogenesis. *Cancer Cell* 2005; **7**: 219–226.

182 Phillips JM, Goodman JI. Identification of genes that may play critical roles in phenobarbital (PB)-induced liver tumorigenesis due to altered DNA methylation. *Toxicol Sci* 2008; **104**: 86–99.

183 El Kasmi KC, Smith AM, Williams L, *et al.* Cutting edge: A transcriptional repressor and corepressor induced by the STAT3-regulated anti-inflammatory signaling pathway. *J Immunol* 2007; **179**: 7215–7219.

184 Al Muftah WA, Al-Shafai M, Zaghlool SB, *et al.* Epigenetic associations of type 2 diabetes and BMI in an Arab population. *Clin Epigenetics* 2016; **8**: 13.

185 Humphries C, Kohli MA, Whitehead P, Mash DC, Pericak-Vance MA, Gilbert J. Alzheimer disease (AD) specific transcription, DNA methylation and splicing in twenty AD associated loci. *Mol Cell Neurosci* 2015; **67**: 37–45.

186 Fernández-Sanlés A, Sayols-Baixeras S, Curcio S, Subirana I, Marrugat J, Elosua R. DNA Methylation and Age-Independent Cardiovascular Risk, an Epigenome-Wide Approach: The REGICOR Study (REgistre GIroní del COR). *Arterioscler Thromb Vasc Biol* 2018; **38**: 645–652.

187 Hwang H-M, Heo C-K, Lee HJ, *et al.* Identification of anti-SF3B1 autoantibody as a diagnostic marker in patients with hepatocellular carcinoma. *J Transl Med* 2018; **16**: 177.

188 Papaemmanuil E, Cazzola M, Boultwood J, *et al.* Somatic SF3B1 mutation in myelodysplasia with ring sideroblasts. *N Engl J Med* 2011; **365**: 1384–1395.

189 Armstrong RN, Steeples V, Singh S, Sanchi A, Boultwood J, Pellagatti A. Splicing factor mutations in the myelodysplastic syndromes: target genes and therapeutic approaches. *Adv Biol Regul* 2018; **67**: 13–29.

190 Siebring-van Olst E, Blijlevens M, de Menezes RX, van der Meulen-Muileman IH, Smit EF, van Beusechem VW. A genome-wide siRNA screen for regulators of tumor suppressor p53 activity in human non-small cell lung cancer cells identifies components of the RNA splicing machinery as targets for anticancer treatment. *Mol Oncol* 2017; **11**: 534–551.

191 Qi J, Nakayama K, Gaitonde S, *et al.* The ubiquitin ligase Siah2 regulates tumorigenesis and metastasis by HIF-dependent and -independent pathways. *Proc Natl Acad Sci USA* 2008; **105**: 16713–16718.

192 Ban WH, Kang HH, Kim IK, *et al.* Clinical significance of nuclear factor erythroid 2-related factor 2 in patients with chronic obstructive pulmonary disease. *Korean J Intern Med* 2018; **33**: 745–752.

193 Cui W, Zhang Z, Zhang P, *et al.* Nrf2 attenuates inflammatory response in COPD/emphysema: Crosstalk with Wnt3a/β-catenin and AMPK pathways. *J Cell Mol Med* 2018; **22**: 3514–3525.

194 Moreno P, Lara-Chica M, Soler-Torronteras R, *et al.* The expression of the ubiquitin ligase SIAH2 (seven in absentia homolog 2) is increased in human lung cancer. *PLoS One* 2015; **10**: e0143376.

195 Hsi ED, Steinle R, Balasa B, *et al.* CS1, a potential new therapeutic antibody target for the treatment of multiple myeloma. *Clin Cancer Res* 2008; **14**: 2775–2784.

196 Tanaka T, Saito Y, Kokuho N, *et al.* Elotuzumab-induced interstitial lung disease: the first case report. *Jpn J Clin Oncol* 2018; **48**: 491–494.

197 Karampetsou MP, Comte D, Kis-Toth K, Kyttaris VC, Tsokos GC. Expression patterns of signaling lymphocytic activation molecule family members in peripheral blood mononuclear cell subsets in patients with systemic lupus erythematosus. *PLoS On*e 2017; **1**2: e0186073.

198 Mattoo H, Mahajan VS, Maehara T, *et al.* Clonal expansion of CD4(+) cytotoxic T lymphocytes in patients with IgG4-related disease. *J Allergy Clin Immunol* 2016; **138**: 825–838.

199 Friend R, Bhutani M, Voorhees PM, Usmani SZ. Clinical potential of SLAMF7 antibodies - focus on elotuzumab in multiple myeloma. *Drug Des Devel Ther* 2017; **11**: 893–900.

200 Bocker MT, Hellwig I, Breiling A, Eckstein V, Ho AD, Lyko F. Genome-wide promoter DNA methylation dynamics of human hematopoietic progenitor cells during differentiation and aging. *Blood* 2011; **117**: e182–9.

201 Smith JA, Zhao W, Wang X, *et al.* Neighborhood characteristics influence DNA methylation of genes involved in stress response and inflammation: The Multi-Ethnic Study of Atherosclerosis. *Epigenetics* 2017; **12**: 662–673.

202 Needham BL, Smith JA, Zhao W, *et al.* Life course socioeconomic status and DNA methylation in genes related to stress reactivity and inflammation: The multi-ethnic study of atherosclerosis. *Epigenetics* 2015; **10**: 958–969.

203 Galvan A, Frullanti E, Anderlini M, *et al.* Gene expression signature of non-involved lung tissue associated with survival in lung adenocarcinoma patients. *Carcinogenesis* 2013; **34**: 2767–2773.

204 Leyva-Leyva M, Sandoval A, Felix R, González-Ramírez R. Biochemical and Functional Interplay Between Ion Channels and the Components of the Dystrophin-Associated Glycoprotein Complex. *J Membr Biol* 2018; **251**: 1–16.

205 Li J, Jiao X, Zhang Q, Hejtmancik JF. Association and interaction of myopia with SNP markers rs13382811 and rs6469937 at ZFHX1B and SNTB1 in Han Chinese and European populations. *Mol Vis* 2017; **23**: 588–604.

206 Ligthart S, Vaez A, Hsu Y-H, *et al.* Bivariate genome-wide association study identifies novel pleiotropic loci for lipids and inflammation. *BMC Genomics* 2016; **17**: 443.

207 Chhabra D, Sharma S, Kho AT, *et al.* Fetal lung and placental methylation is associated with in utero nicotine exposure. *Epigenetics* 2014; **9**: 1473–1484.

208 Sánchez-Vega F, Gotea V, Petrykowska HM, *et al.* Recurrent patterns of DNA methylation in the ZNF154, CASP8, and VHL promoters across a wide spectrum of human solid epithelial tumors and cancer cell lines. *Epigenetics* 2013; **8**: 1355–1372.

209 Ding F-M, Liao R-M, Chen Y-Q, *et al.* Upregulation of SOCS3 in lung CD4+ T cells in a mouse model of chronic PA lung infection and suppression of Th17‑mediated neutrophil recruitment in exogenous SOCS3 transfer in vitro. *Mol Med Rep* 2017; **16**: 778–786.

210 Wang C, Ding H, Tang X, Li Z, Gan L. Effect of liuweibuqi capsules in pulmonary alveolar epithelial cells and COPD through JAK/STAT pathway. *Cell Physiol Biochem* 2017; **43**: 743–756.

211 Zafra MP, Cañas JA, Mazzeo C, *et al.* SOCS3 silencing attenuates eosinophil functions in asthma patients. *Int J Mol Sci* 2015; **16**: 5434–5451.

212 Sun W, Xiao B, Jia A, *et al.* MBD2-mediated Th17 differentiation in severe asthma is associated with impaired SOCS3 expression. *Exp Cell Res* 2018; **371**: 196–204.

213 Pastuszak-Lewandoska D, Domańska-Senderowska D, Antczak A, *et al.* The Expression Levels of IL-4/IL-13/STAT6 Signaling Pathway Genes and SOCS3 Could Help to Differentiate the Histopathological Subtypes of Non-Small Cell Lung Carcinoma. *Mol Diagn Ther* 2018; **22**: 621–629.

214 Zhang W, Jiang M, Chen J, *et al.* SOCS3 Suppression Promoted the Recruitment of CD11b+Gr-1-F4/80-MHCII- Early-Stage Myeloid-Derived Suppressor Cells and Accelerated Interleukin-6-Related Tumor Invasion via Affecting Myeloid Differentiation in Breast Cancer. *Front Immunol* 2018; **9**: 1699.

215 Jiang Z, Chen Z, Li L, Zhou W, Zhu L. Lack of SOCS3 increases LPS-induced murine acute lung injury through modulation of Ly6C(+) macrophages. *Respir Res* 2017; **18**: 217.

216 Tang L, Liu J, Zhu L, *et al.* Curcumin Inhibits Growth of Human NCI-H292 Lung Squamous Cell Carcinoma Cells by Increasing FOXA2 Expression. *Front Pharmacol* 2018; **9**: 60.

217 Fang Y, Ren X, Feng Z. Genetic correlation of SOCS3 polymorphisms with infantile asthma: an evidence based on a case-control study. *Int J Clin Exp Pathol* 2015; **8**: 9586–9591.

218 Kanai M, Akiyama M, Takahashi A, *et al.* Genetic analysis of quantitative traits in the Japanese population links cell types to complex human diseases. *Nat Genet* 2018; **50**: 390–400.

219 Liu F, Zhang H, Lu S, *et al.* Quantitative assessment of gene promoter methylation in non-small cell lung cancer using methylation-sensitive high-resolution melting. *Oncol Lett* 2018; **15**: 7639–7648.

220 Bouzid A, Smeti I, Dhouib L, *et al.* Down-expression of P2RX2, KCNQ5, ERBB3 and SOCS3 through DNA hypermethylation in elderly women with presbycusis. *Biomarkers* 2018; **23**: 347–356.

221 Ali O, Cerjak D, Kent JW, *et al.* Methylation of SOCS3 is inversely associated with metabolic syndrome in an epigenome-wide association study of obesity. *Epigenetics* 2016; **11**: 699–707.

222 Li S, Wong EM, Bui M, *et al.* Inference about causation between body mass index and DNA methylation in blood from a twin family study. *Int J Obes* 2018; published online May 17. DOI:10.1038/s41366-018-0103-4.

223 Chen H, Zhang C, Sheng Y, *et al.* Frequent SOCS3 and 3OST2 promoter methylation and their epigenetic regulation in endometrial carcinoma. *Am J Cancer Res* 2015; **5**: 180–190.

224 Goering W, Kloth M, Schulz WA. DNA methylation changes in prostate cancer. *Methods Mol Biol* 2012; **863**: 47–66.

225 Ohkura T, Taniguchi S, Yamada K, *et al.* Detection of the novel autoantibody (anti-UACA antibody) in patients with Graves’ disease. *Biochem Biophys Res Commun* 2004; **321**: 432–440.

226 Moravcikova E, Krepela E, Prochazka J, Rousalova I, Cermak J, Benkova K. Down-regulated expression of apoptosis-associated genes APIP and UACA in non-small cell lung carcinoma. *Int J Oncol* 2012; **40**: 2111–2121.

227 Zhao M, Liu S, Luo S, *et al.* DNA methylation and mRNA and microRNA expression of SLE CD4+ T cells correlate with disease phenotype. *J Autoimmun* 2014; **54**: 127–136.
